# Small-molecule inhibition of kinesin KIF18A reveals a mitotic vulnerability enriched in chromosomally unstable cancers

**DOI:** 10.1038/s43018-023-00699-5

**Published:** 2023-12-27

**Authors:** Marc Payton, Brian Belmontes, Kelly Hanestad, Jodi Moriguchi, Kui Chen, John D. McCarter, Grace Chung, Maria Stefania Ninniri, Jan Sun, Raffi Manoukian, Stuart Chambers, Seok-Man Ho, Robert J. M. Kurzeja, Katheryne Z. Edson, Upendra P. Dahal, Tian Wu, Sharon Wannberg, Pedro J. Beltran, Jude Canon, Andrew S. Boghossian, Matthew G. Rees, Melissa M. Ronan, Jennifer A. Roth, Sheroy Minocherhomji, Matthew P. Bourbeau, Jennifer R. Allen, Angela Coxon, Nuria A. Tamayo, Paul E. Hughes

**Affiliations:** 1 Oncology Research, Amgen Research, Thousand Oaks, CA USA; 2 Lead Discovery and Characterization, Amgen Research, Thousand Oaks, CA USA; 3 Cytometry Sciences, Amgen Research, Cambridge, MA USA; 4 Research Biomics, Amgen Research, San Francisco, CA USA; 5 Protein Technologies, Amgen Research, Thousand Oaks, CA USA; 6 PKDM, Amgen Research, Thousand Oaks, CA USA; 7 PKDM, Amgen Research, San Francisco, CA USA; 8 Pre-Pivotal Drug Product, Amgen Process Development, Thousand Oaks, CA USA; 9 Inflammation, Amgen Research, Thousand Oaks, CA USA; 10 Broad Institute of MIT and Harvard, Cambridge, MA USA; 11 Translational Safety and Bioanalytical Sciences, Amgen Research, Thousand Oaks, CA USA; 12 Medicinal Chemistry, Amgen Research, Thousand Oaks, CA USA

**Keywords:** Target identification, Cancer

## Abstract

Chromosomal instability (CIN) is a hallmark of cancer, caused by persistent errors in chromosome segregation during mitosis. Aggressive cancers like high-grade serous ovarian cancer (HGSOC) and triple-negative breast cancer (TNBC) have a high frequency of CIN and *TP53* mutations. Here, we show that inhibitors of the KIF18A motor protein activate the mitotic checkpoint and selectively kill chromosomally unstable cancer cells. Sensitivity to KIF18A inhibition is enriched in *TP53*-mutant HGSOC and TNBC cell lines with CIN features, including in a subset of *CCNE1*-amplified, CDK4–CDK6-inhibitor-resistant and *BRCA1*-altered cell line models. Our KIF18A inhibitors have minimal detrimental effects on human bone marrow cells in culture, distinct from other anti-mitotic agents. In mice, inhibition of KIF18A leads to robust anti-cancer effects with tumor regression observed in human HGSOC and TNBC models at well-tolerated doses. Collectively, our results provide a rational therapeutic strategy for selective targeting of CIN cancers via KIF18A inhibition.

## Abstract (web summary)

Payton et al. develop small-molecule inhibitors of kinesin KIF18A able to selectively kill cancer cells with high chromosomal instability and demonstrate that the inhibitors are therapeutically beneficial in cancer patient-derived in vivo models.

## Main

Targeting the mitotic spindle with small-molecule drugs is a validated strategy for treating human cancers; however, collateral damage to normal cells causes myelosuppression and neurotoxicity^1,2^. Microtubule (MT)-targeting agents (MTAs), such as paclitaxel, activate the spindle assembly checkpoint (SAC), leading to cell death either in mitosis or in the subsequent interphase. Recent evidence indicates that paclitaxel can eliminate breast cancer cells by inducing lethal multipolar cell division at pharmacologically active concentrations^3^. Clinical testing of anti-mitotic drugs targeting essential mitotic kinases and kinesins has been unsuccessful due to dose-limiting myelosuppression and a lack of efficacy^4^. Given these challenges, the following factors need consideration to effectively target a mitotic vulnerability in human cancer. First, the druggable gene dependency should be largely dispensable for normal somatic cell division; this will avoid toxicity to highly proliferative bone marrow cells^5^. Second, the druggable gene dependency should preferentially target cancer-specific mitotic defects, such as CIN^6–9^.

CIN is caused by persistent errors in chromosome segregation during mitosis. Factors that contribute to an erosion in mitotic fidelity include alterations in MT dynamics, MT–spindle attachment, aberrant SAC tuning, defects in sister chromatid cohesion and centrosome amplification^6^. In mammalian cells, centrosomes act as MT-organizing centers and contain a pair of barrel-shaped structures, known as centrioles, surrounded by a protein matrix termed the pericentriolar material (PCM)^10^. Cancer cells with extra centrosomes increase multipolar spindle intermediates favoring improper merotelic kinetochore–MT attachments, leading to lagging chromosomes; this provides evidence that extra centrosomes promote CIN^11^. Spindle multipolarity in cancer cells can be suppressed by coalescing centrosomes to form a pseudo-bipolar spindle. This centrosome-clustering mechanism requires kinesin motor proteins such as KIFC1; depletion of KIFC1 promotes multipolarity and loss of cell viability in cancer cells with extra centrosomes^12^. Genetic alterations in *TP53* and several cancer-associated genes (for example, *RB1*, *CCNE1* and *BRCA1*) have been linked to both CIN and centrosome dysregulation^13^. To further explore cancer-specific mitotic vulnerabilities associated with chromosomally unstable aneuploid cells, we focused our investigation on the kinesin motor protein KIF18A.

The kinesin 8 family of motor proteins is composed of KIF18A, KIF18B and KIF19A, which have distinct functions in cell division (KIF18A, KIF18B) and cilia length control (KIF19A)^14^. The KIF18A motor homodimer uses ATP hydrolysis to move processively along kinetochore–MT fibers, accumulating at plus-end MT tips during metaphase, and remains localized at the midzone through cytokinesis^14^. In cell line models, genetic depletion of *KIF18A* leads to increased chromosome oscillation and elongated mitotic spindles^15–17^. In mice, inactivation of *Kif18a* function by gene knockout (KO) leads to defects in male germ cell division, resulting in sterility. However, *Kif18a*^−^^/−^ mice are viable with no gross abnormalities outside of germ cell tissues^18^, suggesting that KIF18A is largely dispensable for somatic cell division. KIF18A expression peaks during mitosis and is elevated in normal tissues that are actively proliferating^16,19^. Overexpression of KIF18A has been reported in a subset of human cancer types (for example, breast and colorectal) and is associated with tumor aggressiveness^20^. A trio of recent reports provided compelling evidence that genetic perturbation of KIF18A selectively reduced the viability of CIN cancer cell lines^21–23^. Here, we examine KIF18A as a potential oncology therapeutic target. We report the discovery and characterization of potent and selective small-molecule inhibitors of KIF18A motor activity and demonstrate promising activity toward CIN cancers.

## Results

### Identification of KIF18A-dependent cancer cell lines

**Fig. 1:**
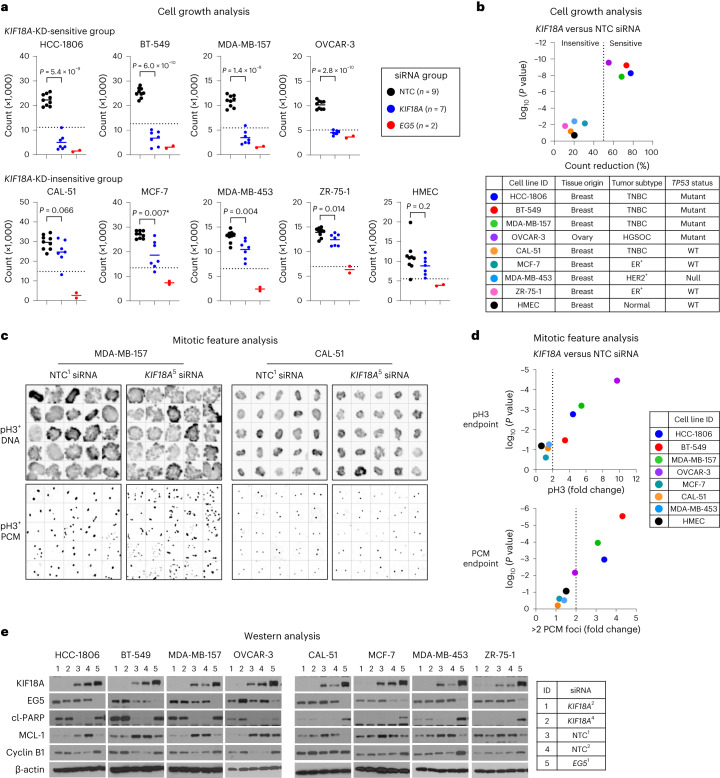
Characterization of *KIF18A* gene dependency in human cell lines. **a**, Cell growth analysis was performed on cell lines (*n* = 9) treated for 96 h with individual siRNA species for *KIF18A* (*n* = 7), *EG5* (*n* = 2) or NTC (*n* = 9). Scatterplots show cell counts for individual siRNA species with group means; the dotted line denotes 50% cell count reduction (*n* = 2 independent experiments in duplicate). Statistical significance was determined for *KIF18A* and NTC siRNA groups by unpaired two-tailed *t*-test (*Welch’s correction) and is shown as *P* values. **b**, Scatterplot shows cell count reduction (%) for each cell line and associated *P* values for *KIF18A* siRNA species relative to NTC siRNA species; the dotted line denotes 50% cell count reduction. Color-coded cell line identification (ID), tissue of origin, tumor subtype (TNBC, HGSOC, ER, human epidermal growth factor receptor 2 (HER2)) and *TP53* status are shown. **c**,**d**, Mitotic imaging analysis was performed on the cell line panel (*n* = 8) treated for 48 h with individual siRNA species for *KIF18A* (*n* = 5) or NTC (*n* = 4). Cells were stained to detect DNA, pH3 and PCM (pericentrin), and images were captured by laser scanning cytometry with a ×40 objective (*n* = 1 or 2 independent experiments in four to eight replicate wells). Color-coded cell line identification is shown. **c**, Representative images of MDA-MB-157 and CAL-51 pH3^+^ cells for NTC (siRNA ID = NTC_1) and *KIF18A* (siRNA ID = h*KIF18A*_5) siRNA species (5 × 5 square gallery). **d**, Scatterplots show cell line pH3^+^ and PCM foci count fold change and associated *P* values for *KIF18A* siRNA species relative to NTC siRNA species; the dotted line denotes twofold change. **e**, WBA was performed on cell lines (*n* = 8) treated for 48 h with individual siRNA species for *KIF18A* (*n* = 2), *EG5* (*n* = 1) or NTC (*n* = 2). Protein levels were determined for KIF18A, EG5, cl-PARP, MCL-1, cyclin B1 and β-actin (*n* = 1 experiment). Immunoblot protein size information is found in the source data. See supporting data (Extended Data Fig. 1 and Supplementary Tables 1, 2 and 7). Source data

**Extended Data Fig. 1:**
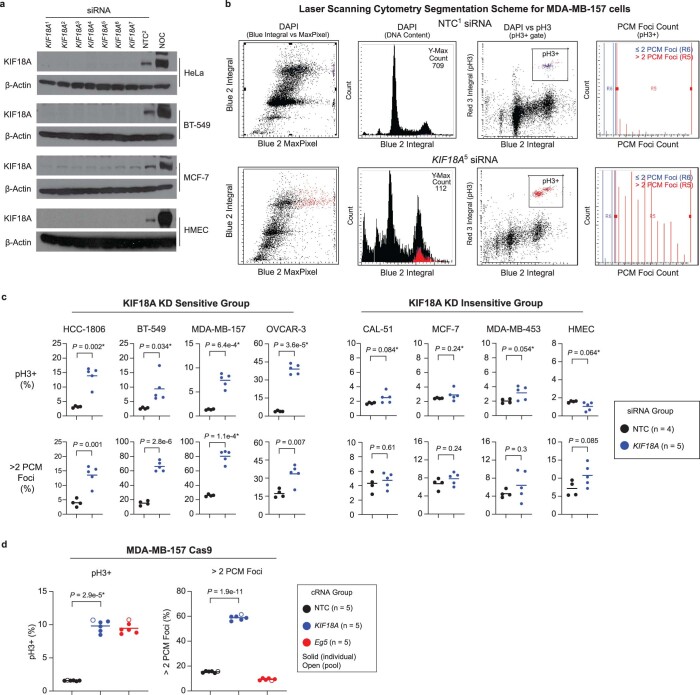
*KIF18A* gene perturbation effects on human cell lines. **a**, WBA was performed on HeLa, BT-549, MCF-7, and HMEC cell lines treated for 48 h with individual siRNA species for *KIF18A* (*n* = 7) or non-targeting control (NTC) (*n* = 1). HeLa cells were treated with nocodazole (NOC) as a mitotic control. Protein levels were determined for KIF18A and β-Actin (*n* = 1 experiment). **b** and **c**, Mitotic image analysis was performed by laser scanning cytometry (LSC). Cell lines were treated for 48 h with individual siRNA species for *KIF18A* (*n* = 5) or NTC (*n* = 4). Imaging data was collected for DNA, phospho-histone H3 (serine-10) (pH3), and PCM (pericentrin) channels with x40 objective. **b**, MDA-MB-157 cells treated with *KIF18A* siRNA (siRNA ID = h*KIF18A*_5) or NTC siRNA (siRNA ID = NTC_1), showing the LSC segmentation scheme (1) Integral versus Max Pixel (DAPI) to establish DNA content profiles, (2) Integral (pH3) versus Integral (DAPI) to gate pH3+ mitotic population, (3) pH3+ PCM foci count scored as >2 PCM foci (red, R5) or ≤ 2 PCM foci (blue, R6). **c**, Scatter plot of each cell line (*n* = 8) shows individual siRNA species for pH3+ and >2 PCM foci count percentages with group means and associated significance (*n* = 1 or 2 independent experiments in 4 to 8 replicate wells). Cell lines grouped as *KIF18A* knockdown (KD) sensitive or insensitive based on 96-h cell growth assay (see Fig. 1a). **d**, Mitotic image analysis was performed by ArrayScan VTi. MDA-MB-157 Cas9 cells were treated for 48 h with tracrRNA complexed to individual or pool crRNA species for *KIF18A* (*n* = 5), NTC (*n* = 5), or *Eg5* (*n* = 5). Imaging data was collected for DNA, pH3, and PCM channels with x20 objective. Scatter plots show individual and pool crRNA species for pH3 + , and >2 PCM foci count percentages with group means and associated significance (*n* = 2 independent experiments in triplicate). Statistical significance was determined for *KIF18A* and NTC siRNA or crRNA groups by unpaired two-tailed *t*-test (*Welch’s correction) and is shown as P values. Immunoblot protein size information is found in the source data. See supporting data (Supplementary Tables 1–2, 7). Source data

To determine the sensitivity and mitotic effects caused by *KIF18A* loss, we compared the impact of *KIF18A* knockdown (KD) versus non-targeting control (NTC) or essential mitotic kinesin *KIF11* (also known as *EG5*) small interfering RNA (siRNA) species on a panel of human breast and ovarian cancer cell lines as well as normal human mammary epithelial cells (HMECs) (Fig. 1a and Extended Data Fig. 1a). Any cell line that showed over 50% inhibition of cell growth for *KIF18A* siRNA species relative to NTC siRNA species was classified as ‘sensitive’ (Fig. 1a,b). The loss of *KIF18A* had a significant effect on the growth of *TP53*-mutant *CCNE1*-amplified cell lines (HCC-1806, MDA-MB-157, OVCAR-3) and retinoblastoma protein (Rb)-deficient BT-549 cells^24^. However, it had only a modest impact on the growth of *TP53*-wild-type (WT) or *TP53*-null cell lines and HMECs (Fig. 1b). *KIF18A*-KD sensitivity tracked with TNBC subtype (three of four cell lines) and whole-genome-doubling (WGD) positivity (three of four cell lines)^21^ (Fig. 1b and Supplementary Table 3).

To further understand the focal dependency on KIF18A, we examined the effects of *KIF18A* KD on the mitotic marker phosphorylated histone H3 on serine 10 (pH3) and the centrosome PCM marker pericentrin. *KIF18A* KD caused a significant increase in pH3 positivity and PCM focus count in sensitive cancer cell lines (Fig. 1c,d and Extended Data Fig. 1b,c). We next investigated whether using CRISPR to KO *KIF18A* would recapitulate the mitotic effects observed by *KIF18A* KD. Indeed, we observed near-perfect concordance in pH3 and PCM foci endpoints by *KIF18A* gene KD and KO in MDA-MB-157 cells (Extended Data Fig. 1c,d). To further study this differential dependency on KIF18A, we performed western blot analysis (WBA) on the cancer cell line panel after treatment with *KIF18A*, *EG5* or NTC siRNA. The depletion of *KIF18A* showed a trend toward increased cyclin B1 (G2M marker) and cl-PARP (apoptosis marker) protein levels as well as decreased MCL-1 (a pro-survival marker) protein levels in those cancer cell lines sensitive to *KIF18A* KD in our cell growth assay (Fig. 1e). Degradation of MCL-1 protein after SAC activation is known to act as a timer for death in mitosis^25,26^. Collectively, these data highlight the identification of breast and ovarian cancer cell lines with a heightened KIF18A dependency; these findings motivated us to perform a small-molecule screen to identify inhibitors of KIF18A motor activity.

### Discovery of potent and selective KIF18A inhibitors

**Fig. 2:**
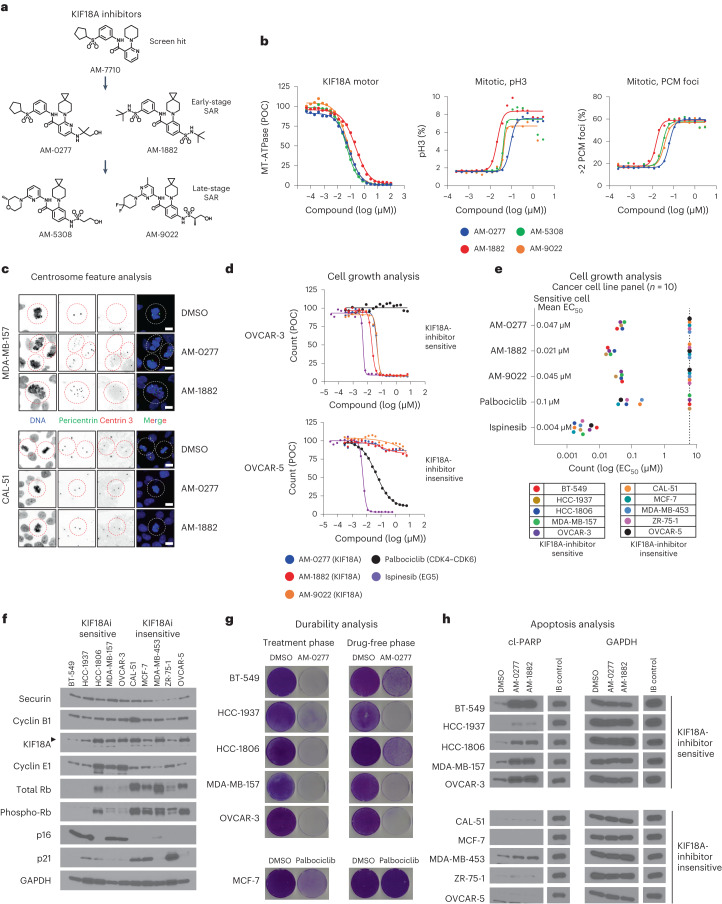
In vitro characterization of potent and selective KIF18A inhibitors. **a**, Structures of AM-7710-series analogs (AM-0277, AM-1882, AM-5308, AM-9022), denoted as early or late SAR stage. **b**, Graph (left) shows compound profiles in a KIF18A motor assay presented as MT-ATPase luminescence signal relative to the percentage of DMSO control (POC), as assessed by ADP-Glo (*n* = 2 or 4 independent experiments). Graphs (center, right) show compound profiles in a 24-h mitotic feature assay for pH3 and PCM foci in MDA-MB-157 cells (*n* = 2 independent experiments). **c**, Centrosome feature analysis in MDA-MB-157 and CAL-51 cells treated for 24 h with DMSO, AM-0277 (0.5 µM) or AM-1882 (0.05 µM) and stained to detect DNA, PCM (pericentrin) and centrioles (centrin 3). Representative images were captured with a ×60 objective (scale bars, 8 µm); dashed lines silhouette mitotic objects (*n* = 1 experiment). **d**,**e**, Cell growth analysis in a panel of cancer cell lines (*n* = 10) treated with DMSO, AM-0277, AM-1882, AM-9022, palbociclib or ispinesib. **d**, Representative concentration–response profiles are presented as count relative to the percentage of the DMSO control. **e**, Graph shows compound count EC_50_ values reported for each cell line and mean EC_50_ values for the sensitive cell lines. The dotted line indicates that >50% cell count reduction was not reached at 6 µM (*n* = 2 independent experiments in duplicate). **f**, WBA across the cancer cell lines (*n* = 10). Protein levels were determined for securin, cyclin B1, KIF18A, cyclin E1, total Rb, phospho-Rb (serine 807 and 811), p16, p21 and glyceraldehyde-3-phosphate dehydrogenase (GAPDH). The arrowhead indicates the KIF18A protein band (*n* = 1 experiment). KIF18A inhibitor (KIF18Ai). **g**, Durability analysis in KIF18A-inhibitor-sensitive cancer cell lines (*n* = 5); cells were treated for 6 d with DMSO or AM-0277 (0.5 µM). MCF-7 cells were treated with palbociclib (1 µM) as a cytostatic control. After the treatment phase (left), cells were replated in drug-free medium and cultured until the DMSO control reached confluence (right), dishes were stained with crystal violet, and images were captured with a digital scanner (*n* = 1 experiment). **h**, Western blot apoptosis analysis of cancer cell lines (*n* = 10) treated for 48 h with DMSO, AM-0277 (0.5 µM) or AM-1882 (0.1 µM). HCC-1806 cells were treated with ispinesib (0.05 µM) as an immunoblot (IB) control. Protein levels were determined for cl-PARP and GAPDH (*n* = 1 experiment). Immunoblot protein size information is found in the source data. See supporting data (Extended Data Figs. 2–4 and Supplementary Table 7). Source data

**Extended Data Fig. 2:**
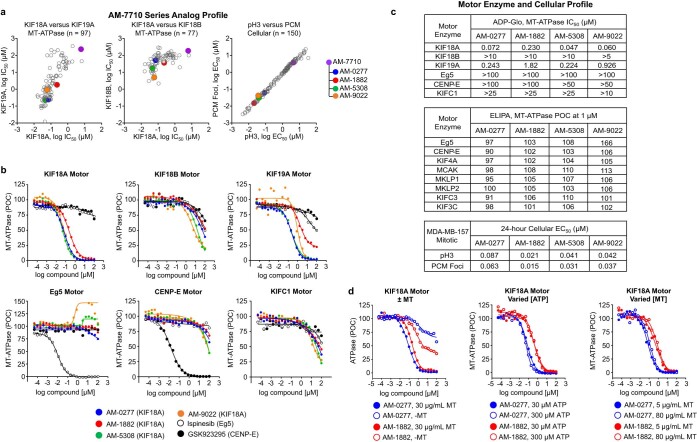
Discovery of potent and selective KIF18A inhibitors. **a**, AM-7710 series analogs were assessed in kinesin-8 microtubule (MT)-ATPase motor assays and 24-h mitotic image assay in MDA-MB-157 cells. Scatter plots show MT-ATPase IC_50_ values for KIF18A versus KIF19A motors (*n* = 97 analogs), KIF18A versus KIF18B motors (*n* = 77 analogs), or cellular EC_50_ values for pH3 versus PCM foci endpoints (*n* = 150 analogs) (*n* ≥ 1 experiment (s) per analog). **b** and **c**, KIF18A compounds were assessed in a panel of kinesin MT-ATPase motor assays and 24-h mitotic image assay in MDA-MB-157 cells. **b**, Graphs show compound profiles for each motor presented as MT-ATPase luminescence signal relative to the percentage of DMSO control (POC), as assessed by ADP-Glo (*n* = 2 or 4 independent experiments). Ispinesib and GSK923295 were included as motor inhibitor controls. **c**, Upper table shows compound MT-ATPase IC_50_ values for KIF18A, KIF18B, KIF19A, Eg5, CENP-E, and KIFC1 motors. Middle table shows compound (1 µM) MT-ATPase POC values for a panel of motors (*n* = 8), as assessed by enzyme-linked inorganic phosphate assay (ELIPA), results presented as a percentage of DMSO control (POC) (*n* = 1 or 2 independent experiments in duplicate). Lower table shows the compound cellular EC_50_ values for mitotic image assay (*n* = 2 independent experiments). **d**, AM-0277 and AM-1882 were assessed in KIF18A ATPase motor assays (± MTs) or varied ATP or MT concentrations, as assessed by ADP-Glo. Graphs show compound profiles in KIF18A motor assays presented as ATPase luminescence signal relative to the percentage of DMSO control (POC) (± MTs *n* = 2 independent experiments in duplicate, varied ATP, or MT concentrations *n* = 1 experiment). Source data

**Extended Data Fig. 3:**
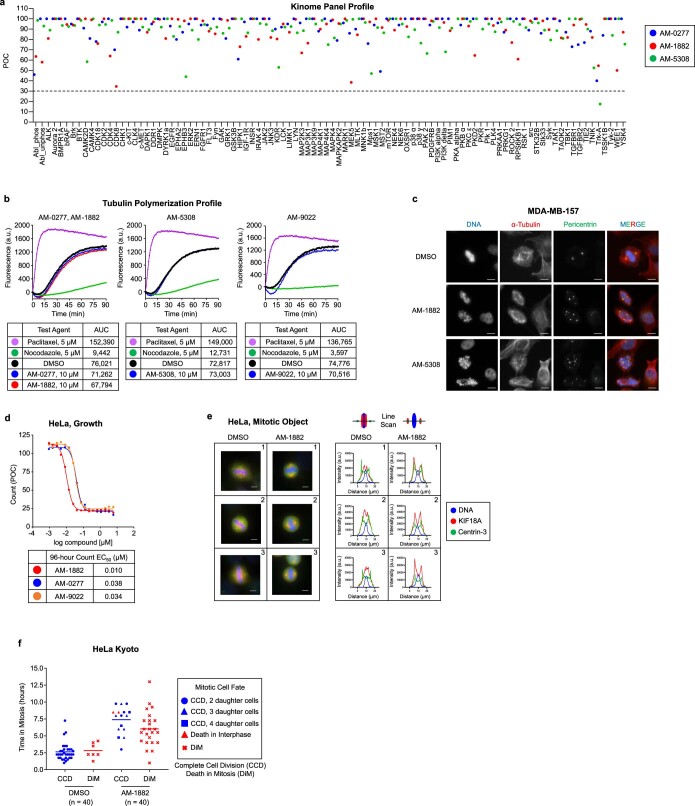
In vitro characterization of potent and selective KIF18A inhibitors. **a**, KIF18A compounds (1 µM) were assessed across a panel of kinases (*n* = 96) by a competitive binding assay. Graph presented as percentage of control (POC); dashed line indicates POC value of 30 (*n* = 1 experiment). **b**, Tubulin polymerization analysis. Profile of KIF18A compounds (10 µM) relative to DMSO, paclitaxel (5 µM, tubulin stabilizer), or nocodazole (5 µM, tubulin destabilizer). Graphs presented as tubulin fluorescence signal at 440 nm expressed as arbitrary units (a.u.) versus time (min) with area-under-the-curve (AUC) values (*n* = 1 or 3 independent experiments). **c**, Mitotic spindle image analysis. MDA-MB-157 cells were treated for 24 h with DMSO, AM-1882 (0.2 µM), or AM-5308 (0.5 µM) and stained to detect DNA, α-tubulin, and pericentrin. Representative images were captured with x40 objective (scale bar = 10 µm) (*n* = 1 experiment). **d**-**f**, KIF18A compound effects on HeLa cells. **d**, Cells were treated for 96 h with DMSO or KIF18A compounds at the indicated concentrations. KIF18A compound concentration-response profiles presented as count relative to the percentage of DMSO control (POC) with count EC_50_ values (*n* = 2 independent experiments in duplicate). **e**, KIF18A protein localization in cells after treatment for 6 hours with DMSO or AM-1882 (0.05 µM). Cells stained to detect DNA, KIF18A, and centrin-3. Representative images of mitotic cells (*n* = 3 per group) were captured with x60 objective (scale bar = 5 µm) and graphs of line scan measurements were obtained for DNA, KIF18A, and centrin-3 channels expressed as fluorescence intensity (a.u.) versus distance (µm) (*n* = 1 experiment). **f**, Mitotic cell fate imaging analysis. HeLa Kyoto cells co-expressing α-tubulin-EGFP and H2B-mCherry proteins released from a G1S block in media containing DMSO or AM-1882 (0.2 µM). Analysis was performed on cells entering the first mitosis (*n* = 40 cells per group). Graph presented as time in mitosis (h) versus mitotic cell fate, either complete cell division (CCD) or death in mitosis (DiM). Classified cells exiting mitosis by the number of daughter cells and cell death in early interphase (*n* = 1 experiment). Images were captured every 15 min for 48 h with x20 objective (see Supplementary Videos 1, 2). Source data

We screened a small-molecule library of diverse compounds for selective inhibitors of KIF18A MT-ATPase motor activity. Two compound hits were discovered that phenocopied the effects of *KIF18A* KD in cells; these hits are structurally distinct from the KIF18A inhibitor BTB-1 (refs. ^27,28^). Next, we initiated a medicinal chemistry campaign to optimize the promising hit, compound 3 (AM-7710)^28^; structure–activity relationship (SAR) efforts led to a quartet of promising series analogs representing early SAR leads (AM-0277, AM-1882) and late SAR leads (AM-5308, AM-9022) (Fig. 2a). All four compounds showed a significant improvement in KIF18A-inhibitory activity and cell potency relative to AM-7710 and exhibited good specificity against a panel of diverse kinesin motor proteins, except for the KIF19A motor (Fig. 2b and Extended Data Fig. 2a–c). The effects of inhibiting KIF19A motor activity (for example, cilia elongation) are distinct from the phenotypes caused by depletion of *KIF18A*, indicating that they will not confound our proof-of-concept studies^14,29^. In the absence of MTs, KIF18A inhibitors failed to block basal KIF18A motor activity (Extended Data Fig. 2d), indicating preferential inhibition of the MT–motor complex, similar to centromere protein E (CENP-E) and KIFC1 inhibitors^30,31^. However, the potency of the KIF18A inhibitors was unaffected by varying ATP and MT concentrations (Extended Data Fig. 2d). This supports our proposed model in which KIF18A inhibitors occupy an allosteric pocket formed by motor helices α4 and α6 close to the motor MT-binding surface^28^. To screen for potential off-target interactions with essential mitotic kinases, three of the KIF18A compounds were profiled against a large panel of kinases. The only binding interaction observed was between TRK-A kinase and AM-5308 tested at 1 µM (Extended Data Fig. 3a). We next investigated whether our KIF18A inhibitors affected tubulin polymerization in vitro in the absence of the KIF18A motor. All four KIF18A compounds had tubulin-polymerization profiles similar to that of the DMSO control and distinct from those of the paclitaxel (MT stabilizer) and nocodazole (MT destabilizer) controls (Extended Data Fig. 3b). These data suggest that our KIF18A inhibitors pose a lower risk of neurotoxicity, as they do not directly interfere with MT dynamics in vitro.

To examine centrosome integrity more closely, MDA-MB-157 and CAL-51 TNBC cells were treated with two KIF18A inhibitors and co-stained for PCM and centriole markers. In MDA-MB-157 cells, inhibiting KIF18A induced multiple PCM centers, most of which were acentriolar and supported spindle arrays (Fig. 2c and Extended Data Fig. 3c). We next examined how inhibiting KIF18A affects the mitotic progression of HeLa cells. We determined that HeLa cells were sensitive to KIF18A-inhibitor treatment and shifted the localization of KIF18A protein from the equatorial region sandwiching the metaphase plate toward the spindle polar region^15,16^ (Extended Data Fig. 3d,e). This localization of inhibited KIF18A protein is consistent with that observed in HeLa cells expressing KIF18A^R308K^ motor-dead protein^32^. Next, we conducted time-lapse imaging in synchronized HeLa Kyoto cells coexpressing chromatin and spindle fluorescent proteins (Supplementary Videos 1 and 2). Treatment with AM-1882 prolonged the duration of mitosis and induced cell death during mitosis or after cell division in interphase (Extended Data Fig. 3f).

**Extended Data Fig. 4:**
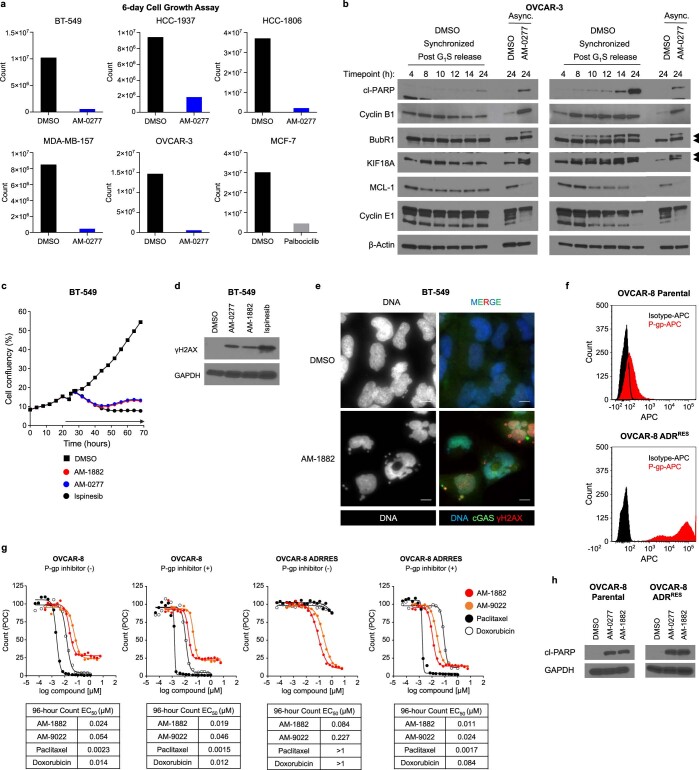
In vitro effects of KIF18A inhibitors on sensitive cancer cell lines. **a**, Six-day cell growth analysis. KIF18A inhibitor-sensitive cancer cell lines were treated with DMSO or AM-0277 (0.5 µM). MCF-7 cells were treated with palbociclib (1 µM) as a cytostatic control. Graphs presented as cell count from 100-mm dish (*n* = 1 experiment). b, WBA of synchronized OVCAR-3 cells released from a G1S block in growth media containing DMSO or AM-0277 (0.5 µM). Cell lysates were prepared at 4, 8, 10, 12, 14, 24 h post-release. Asynchronous cells were treated with DMSO or AM-0277 (0.5 µM) as a control. Protein levels were determined for cl-PARP, cyclin B1, BubR1, KIF18A, MCL-1, cyclin E1, and β-Actin. Arrowheads indicate BubR1 and KIF18A protein band doublets (*n* = 1 experiment). **c**-**e**, KIF18A compound effects on BT-549 cells. **c** and **d**, Cells were treated for 48 h with DMSO, AM-0277 (0.5 µM), or AM-1882 (0.1 µM). Ispinesib (0.05 µM) was included as cytotoxic control. **c**, Cell growth profile presented as cell confluency (%) versus time (h) were captured by live-cell imaging, arrow indicates treatment phase (*n* = 1 experiment in triplicate). **d**, WBA shows protein levels determined for phospho-γ-H2AX (serine-139) (γH2AX) and GAPDH (*n* = 1 experiment). **e**, Images of cells treated for 48 h with DMSO or AM-1882 (0.2 µM) and stained to detect DNA, γH2AX, and cGAS. Representative images were captured with x40 objective (scale bar = 10 µm) (*n* = 1 experiment). **f**-**h**, KIF18A compound effects on parental and ADRRES OVCAR-8 cells. **f**, Determined P-gp protein expression levels versus isotype antibody control by flow cytometry. **g**, Cell growth analysis of OVCAR-8 paired cell lines treated for 96 h with DMSO, AM-1882, or AM-9022 at the indicated concentrations with and without P-gp inhibitor at GF120918 at 1 µM). Paclitaxel and doxorubicin were included as controls. Graphs of compound concentration-response profiles presented as count relative to the percentage of DMSO control (POC) with count EC_50_ values (*n* = 2 independent experiments). **h**, WBA of OVCAR-8 paired cells treated for 48 h with DMSO, AM-0277 (0.5 µM), or AM-1882 (0.1 µM). Protein levels were determined for cl-PARP and GAPDH (*n* = 1 experiment). Immunoblot protein size information is found in the source data. See supporting data (Supplementary Table 7). Source data

Next, we profiled the antiproliferative effects of AM-0277, AM-1882 and AM-9022 on the same panel of cancer cell lines used for our *KIF18A*-KD studies (Fig. 1) and included two additional cell models (HCC-1937 TNBC, OVCAR-5 HGSOC) (Supplementary Table 3). Palbociclib, a CDK4–CDK6 inhibitor, was included as a cytostatic control with enriched sensitivity in Rb-proficient cancer cells^33^. Ispinesib, an EG5 motor inhibitor, was used as a cytotoxic control. The three KIF18A inhibitors showed a focal sensitivity profile across the cell line panel concordant with our *KIF18A*-KD studies and distinct from the palbociclib profile (Fig. 2d,e): the same five of ten cell lines were sensitive to AM-0277, AM-1882 and AM-9022 with mean half-maximum effective concentration (EC_50_) values of 0.047 µM, 0.021 µM and 0.045 µM, respectively. In the group of KIF18A-inhibitor-sensitive cell lines, there was a trend toward increased cyclin E1 and p16 protein levels and decreased total and phosphorylated Rb and p21 protein levels (Fig. 2f). There were no clear expression trends observed in mitotic proteins (securin, cyclin B1, KIF18A) between the two groups. To understand the durability of response after KIF18A-inhibitor treatment, the sensitive cell lines were treated with DMSO or AM-0277 in a 6-d cell growth assay, and the surviving cells were replated in drug-free growth medium and cultured for an additional 7–9 d. As expected, treatment with AM-0277 yielded a significant decrease in cell growth (Fig. 2g, left and Extended Data Fig. 4a). Cells previously exposed to AM-0277 exhibited a marked reduction in cell growth potential relative to the DMSO control (Fig. 2g, right), indicative of a durable growth defect. By contrast, MCF-7 cells previously exposed to palbociclib showed comparable cell growth potential relative to the DMSO control. We next examined how AM-0277 affects the timing of mitotic arrest and cell death in OVCAR-3 cells after release from a G1/S arrest. Inhibition of KIF18A led to cyclin B1 protein accumulation starting at 8 h; this was accompanied by a progressive increase in budding uninhibited by benzimidazole-related 1 (BUBR1) and KIF18A protein doublets, indicating sustained mitotic arrest^34,35^ (Extended Data Fig. 4b). KIF18A-inhibitor treatment showed a trend toward a gradual increase in cl-PARP protein levels, with a parallel decline in MCL-1 and cyclin E1 protein levels, particularly those of the lower-molecular weight isoform of cyclin E1 that affects mitotic progression^36^. The trend toward increased apoptosis measured by cl-PARP protein levels following KIF18A-inhibitor treatment aligned with cell line sensitivity in our cell growth assay (Fig. 2h,e). To probe the downstream consequences of inhibiting KIF18A, we investigated whether DNA damage and micronucleus (MN) formation occurred in BT-549 TNBC cells that lack Rb protein expression (Fig. 2h). Loss of Rb protein is reported to enrich for MN with ruptured membranes, exposing DNA to cytosolic cGAS, a key mediator of pro-inflammatory signaling following chromosome missegregation^37^. KIF18A-inhibitor treatment reduced BT-549 cell growth, increased DNA double-strand breaks as measured by phosphorylation of histone H2AX (γH2AX) protein and induced MN formation that stained positive for γH2AX and/or cGAS proteins (Extended Data Fig. 4c–e).

One mechanism of acquired resistance to chemotherapy in patients with HGSOC involves cellular drug efflux mediated by altered P-glycoprotein (P-gp) expression^38^. To study the impact of P-gp expression on KIF18A-inhibitor anti-cancer activity, we used paired parental and ADR^RES^ OVCAR-8 HGSOC cell lines^39^. First, we verified that ADR^RES^ cells overexpressed P-gp protein (Extended Data Fig. 4f). Next, we treated the paired lines with KIF18A inhibitors, with or without P-gp inhibitor (GF120918) in a cell growth assay (Extended Data Fig. 4g). Paclitaxel and doxorubicin were included as controls that are susceptible to P-gp-mediated efflux^38^. Unlike the chemotherapy controls, co-treatment with KIF18A inhibitor and GF120918 only moderately shifted cell potency (<10-fold) in ADR^RES^ cells. Moreover, KIF18A-inhibitor treatment induced apoptosis in both parental and ADR^RES^ cells (Extended Data Fig. 4h). We conclude that our KIF18A inhibitors remain active in P-gp-expressing HGSOC cells resistant to paclitaxel and doxorubicin.

### Effects of KIF18A inhibition on normal somatic cells

**Fig. 3:**
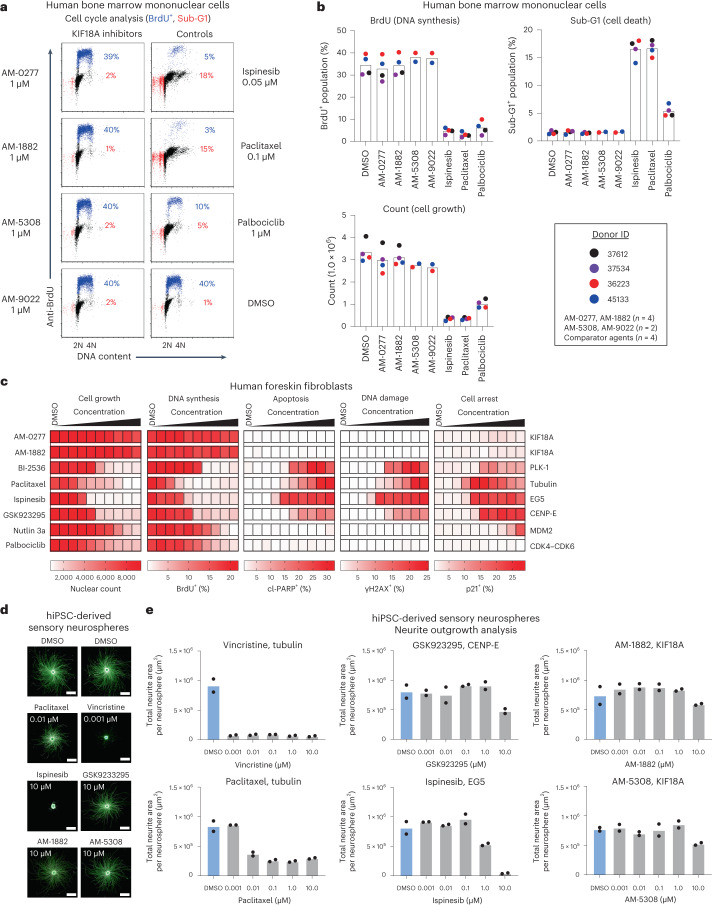
In vitro characterization of KIF18A-inhibitor effects on normal somatic cells. **a**,**b**, Cell cycle (48-h) and cell growth (96-h) analysis of human bone marrow mononuclear cells from unaffected donors (*n* = 2 or 4) treated with DMSO, KIF18A inhibitors (AM-1882, AM-0277, AM-5308, AM-9022 at 1 µM), ispinesib (0.05 µM), paclitaxel (0.1 µM) or palbociclib (1 µM). **a**, Cell cycle analysis; scatterplots show BrdU^+^ (blue) and sub-G1 (red) populations and percentages for donor 36223. **b**, Graphs show cell cycle (BrdU^+^ and sub-G1 population percentages) and cell growth (count) summaries for individual donors with group means (*n* = 2 or 4 independent experiments). **c**, Multiparametric image analysis of human foreskin fibroblast cells treated for 48 h with DMSO, AM-0277 and AM-1882 (KIF18A), BI-2536 (Polo Like Kinase 1, PLK-1), paclitaxel (tubulin), ispinesib (EG5), GSK923295 (CENP-E), nutlin 3a (Mouse Double Minute 2, MDM2) or palbociclib (CDK4–CDK6). Cells were stained to detect DNA, BrdU, cl-PARP, γH2AX and p21. Heatmaps show nuclear count (growth) and percentage of counts that stain positive for BrdU (DNA synthesis), cl-PARP (apoptosis), phosphorylated γH2AX (DNA damage) and p21 (cell arrest); scales are indicated below each heatmap (*n* = 1 experiment). **d**,**e**, Neurite outgrowth analysis of hiPSC-derived sensory neurospheres treated for 24 h with DMSO, vincristine, paclitaxel, ispinesib, GSK923295, AM-1882 or AM-5308 at the indicated concentrations. Neurospheres were stained to detect DNA (blue) and β3-tubulin protein (green). **d**, Representative images of neurite outgrowth (scale bars, 500 µm) captured with a ×20 objective. **e**, Concentration–response graphs presented as total neurite area (µm^2^) per neurosphere with group means (*n* = 2 independent experiments in duplicate or triplicate). See supporting data (Extended Data Fig. 5). Source data

**Extended Data Fig. 5:**
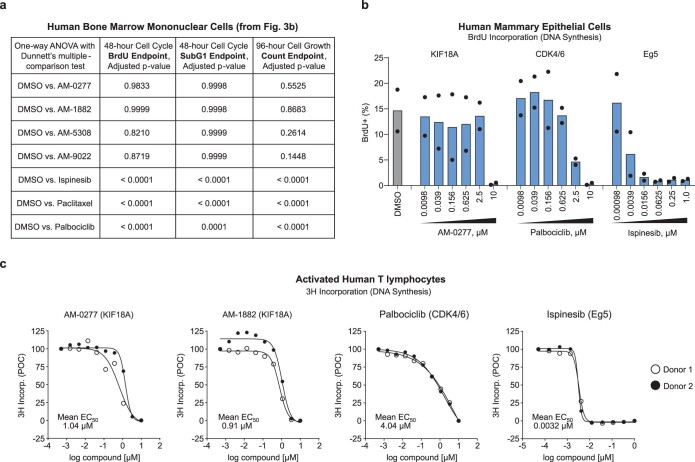
In vitro effects of KIF18A inhibitors on normal somatic cells. **a**, Cell-cycle (48-h) and cell growth (96-h) analysis of human bone marrow mononuclear cells from normal donors (*n* = 2 or 4) treated with DMSO, KIF18A compounds (AM-1882, AM-0277, AM-5308, AM-9022 at 1 µM), ispinesib (0.05 µM), paclitaxel (0.1 µM), or palbociclib (1 µM) (see Fig. 3b). Each donor assessed in independent experiments. Table shows statistical significance for treatment groups relative to DMSO control by One-way ANOVA with Dunnett’s multiplicity adjustment and is shown as P values. **b**, Human mammary epithelial cell (HMEC) BrdU incorporation analysis by flow cytometry. Cells were treated for 48 h with DMSO, AM-0277, ispinesib, or palbociclib at the indicated concentrations (*n* = 2 independent experiments). Graphs show concentration-response profiles presented as the percentage of BrdU+ cells for each treatment condition. **c**, Activated human T cells 3H-thymidine incorporation analysis. Cells from two normal donors were treated for 48 h with DMSO, AM-1882, AM-0277, ispinesib, or palbociclib at the indicated concentrations (*n* = 1 experiment in triplicate). Graphs show concentration-response profiles presented as 3H-thymidine incorporation relative to the percentage of DMSO control (POC) for each donor with mean EC_50_ values. Source data

Toxicity affecting normal cell division in tissues such as bone marrow has limited the clinical utility of small-molecule inhibitors targeting essential mitotic kinases and kinesins^4,5^. To address this concern, proliferating human bone marrow mononuclear cells from healthy donors were treated ex vivo with our KIF18A inhibitors at 1 µM. Cell cycle and cell growth analysis of human bone marrow mononuclear cells showed similar results between all four KIF18A inhibitors and the DMSO control, distinct from the three comparator agents (ispinesib, paclitaxel, palbociclib), which significantly reduced bone marrow cellularity (Fig. 3a,b and Extended Data Fig. 5a). We next examined the effects of KIF18A inhibitors on human foreskin fibroblast cells using a multiparametric imaging assay (endpoints: count, 5-bromodeoxyuridine (BrdU), cl-PARP, γH2AX, p21) and included a panel of comparator agents. Strikingly, KIF18A-inhibitor treatment over a broad concentration range had minimal effects on human foreskin fibroblast cells, suggesting that its motor activity is dispensable for cell division (Fig. 3c). By contrast, all four anti-mitotic agents (BI-2536, paclitaxel, ispinesib, GSK923295) showed potent cytotoxic effects. To extend our analysis to other normal cell types, we treated proliferating HMECs or activated human T lymphocytes with our KIF18A inhibitors in a BrdU- or 3H-thymidine-incorporation assay, respectively. KIF18A-inhibitor treatment showed inhibition of DNA synthesis in both normal cell types that was well above the effective concentration (>20-fold) in sensitive cancer cell lines (Extended Data Fig. 5b,c and Fig. 2e). To investigate the potential effects of KIF18A inhibition on sensory neuron features, we evaluated AM-1882 and AM-5308 in a neurite outgrowth assay using human induced pluripotent stem cell (hiPSC)-derived sensory neurospheres. We included motor inhibitors (ispinesib, GSK923295) and MTAs (vincristine, paclitaxel) as comparator agents. Notably, the KIF18A inhibitors had no effect on neurite outgrowth, except for a partial reduction at 10 µM (Fig. 3d,e). By contrast, treatment with the two MTAs potently inhibited neurite outgrowth. Collectively, these data support that our KIF18A inhibitors have a favorable in vitro profile against a panel of normal cell types, which is distinct from myelosuppressive and neurotoxic anti-mitotic agents. Nevertheless, higher micromolar concentrations of our KIF18A inhibitors may adversely affect normal cells.

### AM-1882 PRISM profile phenocopies *KIF18A* gene dependency

**Fig. 4:**
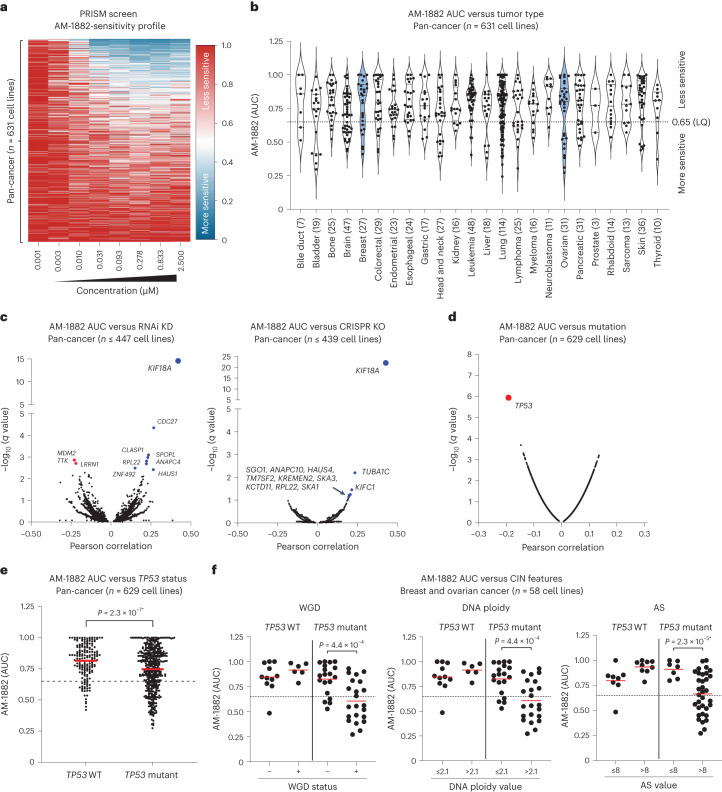
AM-1882 phenocopies *KIF18A* gene dependency across a panel of DNA-barcoded cancer cell lines. **a**–**f**, Pooled cancer cell lines were treated with AM-1882 in a 5-d cell growth assay; the relative abundance of unique barcodes estimates cell viability (*n* = 1 experiment in triplicate). AM-1882 AUC values were determined for cancer cell lines (*n* = 631). **a**, Heatmap of the AM-1882 concentration–response profile; viability values for AM-1882 are presented as fold change relative to the DMSO control; a scale is shown on the right, with viability scoring as more sensitive (blue) or less sensitive (red). **b**, Violin plots show AM-1882 AUC distribution by tumor type (*n* = 24); numbers of cell lines per type are denoted on the *x* axis. The dotted line indicates an AUC value of 0.65, representing the lower-quartile (LQ) cutoff. **c**, AM-1882 AUC versus genome-wide gene dependency scores from RNAi KD (*n* ≤ 447 cell lines) or CRISPR KO (*n* ≤ 439 cell lines). Volcano plots show Pearson correlation scores and *q* values for 10,000 genes. *KIF18A* gene KD or KO was scored as the top-ranked correlation with AM-1882 sensitivity; other genes with positive or negative correlations with AM-1882 sensitivity are denoted in blue or red, respectively. **d**,**e**, AM-1882 AUC versus gene mutation (*n* = 629 cell lines). **d**, Volcano plot shows Pearson correlation scores and *P* values for 10,000 genes. *TP53* gene mutation was scored as the top-ranked correlation with AM-1882 sensitivity. **e**, Scatterplot shows AM-1882 AUC versus *TP53*-WT or *TP53*-mutant group with mean AUC values for each group. The dashed line indicates an AUC value of 0.65. Statistical significance was determined for the *TP53*-WT group relative to the *TP53*-mutant group by unpaired two-tailed *t*-test (*Welch’s correction) and is shown as *P* values. **f**, AM-1882 AUC versus CIN features (WGD, DNA ploidy, AS) in breast and ovarian cancer cell lines (*n* = 58). Scatterplots show AM-1882 AUC versus *TP53* status plus CIN features with mean AUC values for each group. Dashed lines indicate an AUC value of 0.65. Statistical significance was determined for CIN features in the *TP53*-mutant group by unpaired two-tailed *t*-test (*Welch’s correction) and is shown as *P* values. See supporting data (Extended Data Fig. 6 and Supplementary Tables 3–5). Source data

**Extended Data Fig. 6:**
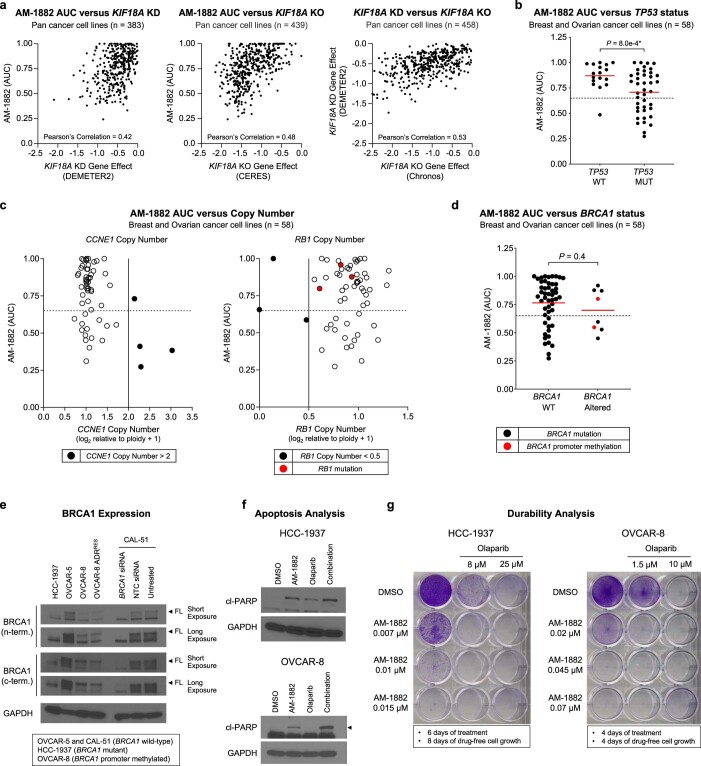
AM-1882 PRISM association analysis and combinability potential of KIF18A and PARP inhibition. **a**-**d**, Pooled cancer cell lines treated with AM-1882 in a 5-d cell growth assay; the relative abundance of unique barcodes estimate cell viability (*n* = 1 experiment in triplicate). AM-1882 area-under-the-curve (AUC) values were determined for cancer cell lines (*n* = 631). **a**, Scatterplots show the pan-cancer AM-1882 AUC distribution versus *KIF18A* KD or KO Gene Effect scores and Pearson correlation coefficient values. *KIF18A* KD versus KO Gene Effect scores were included as a reference. **b**-**d**, AM-1882 PRISM association analysis in breast and ovarian cancer cell lines (*n* = 58). The dotted line indicates an AUC value of 0.65. **b**, Scatterplot shows AM-1882 AUC versus *TP53* status grouped by wildtype (WT) or mutant (MUT) cell lines with mean AUC indicated for each group. **c**, Scatterplots show AM-1882 AUC versus *CCNE1* or *RB1* copy number. Solid line indicates either a log2 copy number value of 2 (*CCNE1*) or 0.5 (*RB1*), black circle indicates a copy number >2 or < 0.5, respectively. Red circle indicates *RB1* mutant cell lines. **d**, Scatterplot shows AM-1882 AUC versus *BRCA1* status, cell lines grouped by WT or altered (MUT as black circle, promoter methylated (PM) as red circle) with mean AUC values indicated for each group. **b** and **d**, Statistical significance was determined for the *BRCA1* groups by unpaired two-tailed *t*-test (*Welch’s correction) and is shown as P values. **e**, WBA to detect full-length (FL) BRCA1 protein in HCC-1937 (*BRCA1* MUT), OVCAR-5 (*BRCA1* WT), OVCAR-8 (*BRCA1* PM), and CAL-51 (*BRCA1* WT) cell lines. CAL-51 cells were treated for 48 h with pooled *BRCA1* or NTC siRNAs as knockdown controls. Protein levels were determined for BRCA1 using n-terminal or c-terminal antibodies and GAPDH. Arrowhead indicates FL BRCA1 protein band (*n* = 1 experiment). **f** and **g**, BRCA1-altered cell lines were treated with AM-1882 alone, olaparib alone, or in combination. **f**, WBA to detect apoptosis. HCC-1937 cells were treated for 48 h with DMSO, AM-1882 (0.01 µM), olaparib (20 µM), or in combination. OVCAR-8 cells were treated with DMSO, AM-1882 (0.03 µM), olaparib (5 µM), or in combination. Protein levels were determined for cl-PARP and GAPDH. **g**, Durability analysis after drug withdrawal. HCC-1937 or OVCAR-8 cells were treated for 6 d or 4 d, respectively, with DMSO, AM-1882, olaparib, or in a 3 × 2 combination matrix at the indicated concentrations. After the treatment phase, cell were re-plated in drug-free growth media and cultured until DMSO control reached confluence, plates were stained with crystal violet, and images were captured by digital scanner (*n* = 1 experiment). Immunoblot protein size information is found in the source data. See supporting data (Supplementary Tables 1, 3, 5, 7). Source data

To investigate the antiproliferative effects of our KIF18A inhibitors more broadly, we conducted a PRISM (profiling relative inhibition simultaneously in mixtures) screen with AM-1882 on a large panel of DNA-barcoded cancer cell lines, using a 5-d cell growth assay^40,41^. AM-1882 exhibited a focal cytotoxic sensitivity profile across the panel of cancer cell lines (*n* = 631) (Fig. 4a), and the effective concentration range aligned with the results obtained in the 96-h cell growth assay (Fig. 2d,e). Next, we plotted AM-1882 sensitivity by tumor type based on the area under the curve (AUC) (Fig. 4b). Cell lines with an AUC ≤ 0.65, representing the lower quartile, were defined as ‘more sensitive’ (Supplementary Table 3). We compared AM-1882 AUC values with genome-wide genetic dependency scores (CRISPR KO, RNA interference (RNAi) KD) for the cancer cell line panel. The top-ranked positive correlation with AM-1882 AUC was *KIF18A* gene-KO and -KD, with Pearson correlation scores of 0.48 (*q* value = 9.6 × 10^−23^) and 0.42 (*q* value = 2.8 × 10^−15^), respectively (Fig. 4c and Extended Data Fig. 6a). No other genes were as highly correlated as *KIF18A*, confirming the target specificity of AM-1882. We next compared AM-1882 AUC values with gene mutation status. The top-ranked correlated gene was *TP53* with a Pearson score of −0.19 (*P* value = 1.1 × 10^−6^, *q* value = 0.021), where 28% (126 of 448) of *TP53*-mutant and 14% (26 of 181) of *TP53*-WT cell lines had AM-1882 AUC ≤ 0.65 (Fig. 4d,e). Our pan-cancer association analysis did not reveal enrichment of AM-1882 sensitivity with other cancer gene alterations (for example, *CCNE1*, *RB1* or *BRCA1*).

To better understand the activity of AM-1882 in breast and ovarian cancer cell lines (*n* = 58), we performed association analysis on a subset of features using the same AUC sensitivity cutoff of ≤0.65. Based on this scoring criteria, AM-1882 sensitivity was enriched in *TP53*-mutant serous ovarian cancer (55%, six of 11) and estrogen receptor (ER)-negative breast cancer (44%, seven of 16) subtypes (Supplementary Table 3). By contrast, none of the endometrioid ovarian cancer (0%, 0 of six) or ER-positive breast cancer (0%, 0 of ten) subtypes were sensitive to AM-1882 treatment. Like the pan-cancer analysis, AM-1882 sensitivity was significantly enriched in *TP53*-mutant (39%, 16 of 41) relative to *TP53*-WT (6%, one of 17) cell lines (*P* value = 8.0 × 10^−4^; Extended Data Fig. 6b). Next, we divided the cell lines by CIN features (WGD, ploidy, aneuploidy score (AS)) and further subdivided them based on *TP53* status. AM-1882 sensitivity was enriched in the *TP53*-mutant cell lines that were WGD^+^ with >2.1 ploidy (55%, 12 of 22 sensitive) relative to WGD^−^ with ≤2.1 ploidy (21%, 4 of 19 sensitive) (*P* value = 4.4 × 10^−4^; Fig. 4f). In addition, AM-1882 sensitivity was enriched in *TP53*-mutant cell lines with AS > 8 (47%, 16 of 34 sensitive) relative to AS ≤ 8 (0%, 0 of seven sensitive) (*P* value = 2.3 × 10^−5^). Next, we compared AM-1882 sensitivity with RNA expression levels and found that the strongest positively and negatively correlated genes were *LRRC28* and *RGS20*, respectively (Supplementary Table 4). However, gene set enrichment analysis of the top hits did not reveal overrepresented genes associated with mitosis, cell cycle or the centrosome. Lastly, we evaluated *CCNE1*, *RB1* and *BRCA1* gene alterations known to be enriched in HGSOC and TNBC tumors^42,43^. AM-1882 showed lower AUC values (≤0.65) in three of four *CCNE1*-amplified, one of six *RB1*-deleted or -mutated and four of eight *BRCA1*-altered (mutation or promoter methylation) cell lines (Extended Data Fig. 6c,d and Supplementary Table 5). We observed reduced full-length breast cancer 1 (BRCA1) protein levels in HCC-1937 and OVCAR-8 cells, likely due to *BRCA1* gene mutation and promoter methylation, respectively^44^ (Extended Data Fig. 6e and Supplementary Table 5). Notably, treatment with AM-1882 in combination with the PARP inhibitor olaparib enhanced apoptosis and suppression of cell growth in HCC-1937 and OVCAR-8 cells (Extended Data Fig. 6f,g). Collectively, these data suggest that AM-1882 faithfully phenocopies *KIF18A* gene-KO and -KD dependencies across cancer cell lines. Sensitivity to KIF18A inhibition is enriched in *TP53*-mutant breast and ovarian cancer cell lines with CIN features. Furthermore, AM-1882 is active in a subset of cancer cell lines with *CCNE1*, *BRCA1* and *RB1* gene alterations and can enhance apoptosis when combined with PARP inhibition.

### Characterization of KIF18A inhibition in vivo

**Extended Data Fig. 7:**
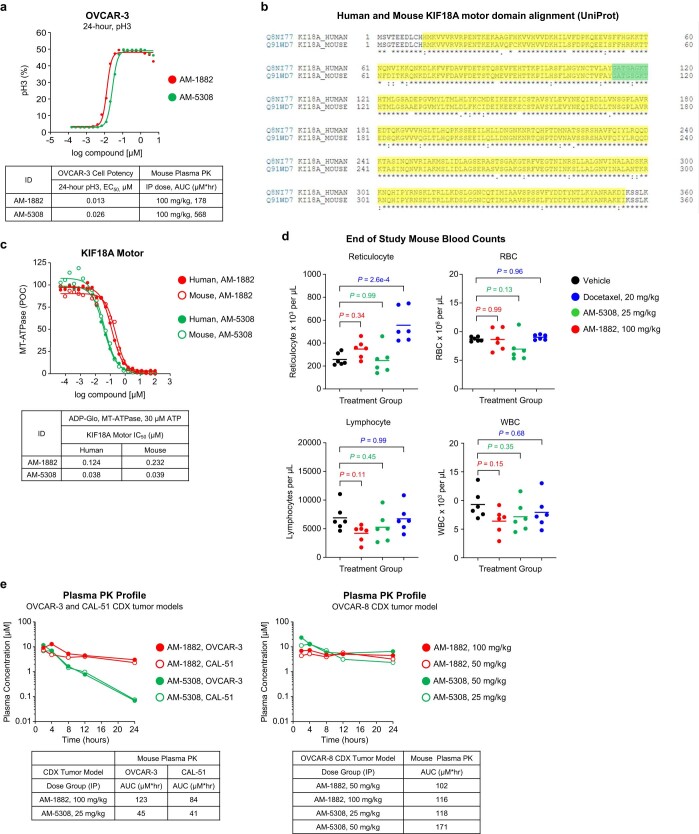
Characterization of intraperitoneal candidates AM-1882 and AM-5308. **a**, Mitotic pH3 and pharmacokinetic (PK) analysis. OVCAR-3 cells were treated for 24 h with DMSO, AM-1882, or AM-5308 at indicated concentrations. Cells were stained to detect DNA and pH3. Graph of compound concentration-response profiles presented as pH3+ percentage and EC_50_ values (*n* = 3 independent experiments in triplicate). Mouse plasma PK area-under-the-curve (AUC) values (µM·h) were determined after a single intraperitoneal (i.p.) dose of AM-1882 (100 mg per kg) or AM-5308 (100 mg per kg) (*n* = 3 mice per group). **b**, Amino acid sequence alignment of human and mouse KIF18A motor domains. Protein sequences Q8NI77 (human) and Q91WD7 (mouse), motor domain, and nucleotide binding site are highlighted in yellow and green, respectively. **c**, Human and mouse KIF18A motor MT-ATPase assays performed for AM-1882 and AM-5308, as assessed by ADP-Glo. Graph of compound concentration-response profiles presented as MT-ATPase luminescence signal relative to the percentage of DMSO control (POC) and IC_50_ values (*n* = 2 independent experiments). **d**, End of study mouse blood counts from OVCAR-3 CDX tumor model. Mice were administered vehicle, AM-1882 (100 mg per kg), or AM-5308 (25 mg per kg) daily for 18 d. Mice were administered docetaxel (20 mg per kg) once weekly as a positive control. Blood samples were collected after the last dose (*n* = 6 mice per group). Scatterplots show individual and mean blood counts for reticulocytes, red blood cells (RBC), lymphocytes, and white blood cells (WBC). Statistical significance was determined for treatment groups relative to vehicle by One-way ANOVA with Dunnett’s multiplicity adjustment and is shown as P values. **e**, End of study mouse PK profiles from OVCAR-3, CAL-51, and OVCAR-8 CDX tumor models. Scatterplots show mouse PK profiles presented as mean plasma concentration versus time (*n* = 2 mice per time point) with AUC values (µM·h). Source data

To examine the effects of KIF18A inhibition in vivo, we selected AM-1882 and AM-5308 based on their acceptable plasma exposures achieved by intraperitoneal (i.p.) dosing in rodents and their double-digit nanomolar potency in the OVCAR-3 pH3 mitotic marker assay (Extended Data Fig. 7a). We confirmed that AM-1882 and AM-5308 have similar inhibitory effects on the motor activity of human and mouse KIF18A, which share 90% amino acid identity in their motor domains (Extended Data Fig. 7b,c). This allows us to evaluate KIF18A-inhibitor tolerability in mice.

**Fig. 5:**
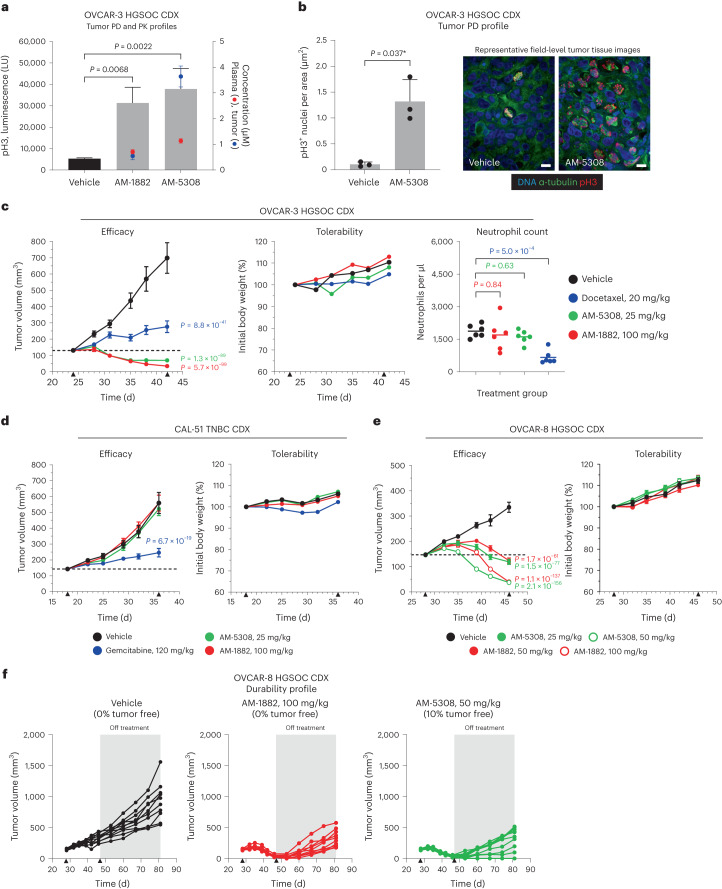
Characterization of KIF18A inhibition in vivo. **a**, PD and PK profiles of two KIF18A compounds in the OVCAR-3 HGSOC CDX tumor model. Mice were administered a single i.p. dose of vehicle, AM-1882 (100 mg per kg) or AM-5308 (50 mg per kg). Tumor and blood were collected 24 h after treatment and analyzed for pH3 signal in tumor and compound PK in tumor and plasma. Graph shows pH3 luminescence signal (LU) with mean + s.d. (bar, left axis) and tumor (blue) and plasma (red) concentrations with mean ± s.d. (right axis) for each treatment group (*n* = 3 mice per group). Statistical significance was determined for treatment groups relative to the vehicle by one-way ANOVA with Dunnett’s multiplicity adjustment and is shown as *P* values. **b**, PD imaging analysis of AM-5308 in the OVCAR-3 HGSOC CDX tumor model. Mice were administered an i.p. dose of vehicle or AM-5308 (25 mg per kg) for 2 d. Tumors were collected 24 h after treatment. Tumors were stained to detect DNA, pH3 and α-tubulin; representative images were captured with a ×60 objective (scale bars, 12 µm). Graph shows pH3^+^ nucleus count per tissue area with mean + s.d. from three image fields per tumor (*n* = 3 mice per group). Statistical significance was determined for AM-5308 relative to the vehicle by unpaired two-tailed *t*-test (*Welch’s correction) and is shown as *P* values. **c**,**d**, AM-1882 and AM-5308 efficacy and tolerability analysis in OVCAR-3 HGSOC (**c**) and CAL-51 TNBC (**d**) CDX tumor models. Mice were administered an i.p. dose of vehicle, AM-1882 (100 mg per kg) or AM-5308 (25 mg per kg) daily for 18 d. Mice were administered an i.p. dose of docetaxel (20 mg per kg) once weekly (**c**) or gemcitabine (120 mg per kg) twice weekly (**d**) as positive controls. Graphs show tumor volume and mouse body weight measurements as mean ± s.e.m. versus time (d) (*n* = 10 mice per group); dashed line indicates 100% TGI or tumor stasis. Treatment start and stop (▴) are indicated on the *x* axis. **c**, Graph shows end-of-study neutrophil counts from the OVCAR-3 study (*n* = 6 mice per group). Statistical significance was determined for neutrophil counts for treatment groups relative to the vehicle by one-way ANOVA with Dunnett’s multiplicity adjustment and is shown as *P* values. **e**,**f**, AM-1882 and AM-5308 efficacy, tolerability and durability analysis in the OVCAR-8 HGSOC CDX tumor model. Mice were administered an i.p. dose of vehicle, AM-1882 (50 or 100 mg per kg) or AM-5308 (25 or 50 mg per kg) daily for 18 d. **e**, Graphs show tumor volume and body weight measurements as mean ± s.e.m. versus time (d) (*n* = 10 mice per group); the dashed line indicates 100% TGI. Treatment start and stop (▴) are indicated on the *x* axis. **f**, After the cessation of treatment (shaded gray area), tumor durability analysis was performed until day 81. Graphs show tumor volume measurements versus time (d) for individual mice. Mice with no measurable tumor are indicated as tumor free. Statistical significance was determined for tumor efficacy for treatment groups relative to the vehicle by a linear mixed-effect analysis model with Dunnett’s multiplicity adjustment and is shown as *P* values. See supporting data (Extended Data Fig. 7). Source data

To investigate the pharmacodynamic (PD) effects of our KIF18A inhibitors, mice with established OVCAR-3 cell line-derived xenograft (CDX) tumors were administered vehicle, AM-1882 at 100 mg per kg or AM-5308 at 50 mg per kg. Tumor and blood samples were collected 24 h after treatment for pH3 PD and pharmacokinetic (PK) analysis. AM-1882 and AM-5308 increased pH3 mitotic marker levels in OVCAR-3 tumors by 5.9-fold (*P* value = 0.0068) and 7.1-fold (*P* value = 0.0022), respectively (Fig. 5a). AM-5308 exposure was higher in the tumor relative to plasma, whereas AM-1882 exposure was similar in both. Next, we selected AM-5308 for tumor PD assessment by imaging. Mice with established OVCAR-3 tumors were administered vehicle or AM-5308 at 25 mg per kg for 2 d. AM-5308 increased pH3 mitotic marker counts in OVCAR-3 tumors by 12.7-fold (*P* value = 0.037), with evidence of abnormal mitotic cell features (Fig. 5b).

To establish whether the robust PD effects observed with our KIF18A inhibitors would result in efficacy, mice with established OVCAR-3 tumors were administered vehicle, AM-1882 at 100 mg per kg or AM-5308 at 25 mg per kg daily for 18 d. As a positive control, mice were administered docetaxel at 20 mg per kg once weekly. AM-1882 and AM-5308 inhibited tumor growth (*P* values ≤ 1.3 × 10^−89^) with evidence of tumor regression (TR; 73% and 46%, respectively) (Fig. 5c). Treatment with docetaxel resulted in 74% tumor growth inhibition (TGI) (*P* value = 8.8 × 10^−41^). Our KIF18A inhibitors were well tolerated by the mice with no changes in body weight or blood counts (Fig. 5c and Extended Data Fig. 7d). By contrast, docetaxel decreased neutrophil counts (*P* value = 5.0 × 10^−4^). At the end of the study, plasma AUC values for AM-1882 and AM-5308 were 123 and 45 µM·h, respectively (Extended Data Fig. 7e). Next, we evaluated the efficacy of our KIF18A inhibitors in the near-diploid CIN^−^ CAL-51 CDX tumor model using the same dose and schedule. As a positive control, mice were administered gemcitabine at 120 mg per kg twice weekly. In contrast to the CIN^+^ OVCAR-3 tumor model, the KIF18A inhibitors showed no effect on CAL-51 tumor growth, while gemcitabine was efficacious with 75% TGI (*P* value = 6.7 × 10^−19^; Fig. 5d). KIF18A inhibitors were well tolerated by mice and had comparable plasma exposures in both studies (Fig. 5d and Extended Data Fig. 7e).

To further examine the in vivo activity of our KIF18A inhibitors, we evaluated the OVCAR-8 CDX tumor model. Mice with established tumors were administered vehicle, AM-1882 at 50 or 100 mg per kg or AM-5308 at 25 or 50 mg per kg daily for 18 d. AM-1882 and AM-5308 inhibited tumor growth (*P* values ≤ 1.7 × 10^−61^) with evidence of TR (16% or 73% TR and 19% or 75% TR, respectively) (Fig. 5e). As before, inhibition of KIF18A was well tolerated by the mice (Fig. 5e). The plasma AUC value for AM-5308 at 25 mg per kg was 2.6-fold higher in the OVCAR-8 study, while AM-1882 showed similar exposures across CDX studies (Extended Data Fig. 7e). After treatment cessation, we monitored the mice to determine the durability of treatment and the timing of tumor regrowth. The OVCAR-8 tumors that regressed on KIF18A-inhibitor treatment showed a delayed resumption in growth, except for in one animal in the AM-5308 group (Fig. 5f). Collectively, these data demonstrate that i.p.-administered KIF18A inhibitors have robust anti-cancer activity at well-tolerated doses in mice.

### Oral candidate AM-9022 induces TR in vivo

**Extended Data Fig. 8:**
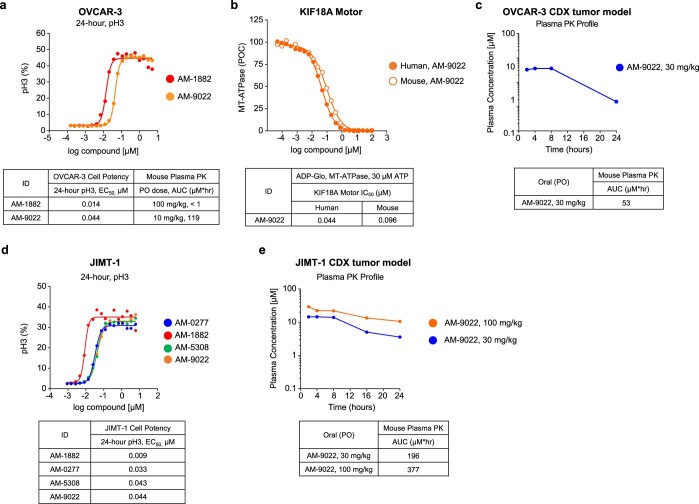
Characterization of oral candidate AM-9022. **a**, Mitotic pH3 and pharmacokinetic (PK) analysis. OVCAR-3 cells were treated for 24 h with DMSO, AM-1882, or AM-9022 at indicated concentrations. Cells were stained to detect DNA and pH3. Graphs of compound concentration-response profiles presented as pH3 percentage and EC_50_ values (*n* = 2 independent experiments in duplicate). Mouse plasma PK area-under-the-curve (AUC) values (µM·h) were determined after a single oral (p.o.) dose of AM-1882 (100 mg per kg) or AM-9022 (10 mg per kg) (*n* = 3 mice per group). **b**, Human and mouse KIF18A motor MT-ATPase assays performed for AM-9022, as assessed by ADP-Glo. Graph of AM-9022 concentration-response profiles presented as MT-ATPase luminescence signal relative to percentage of DMSO control (POC) and IC_50_ values (*n* = 2 independent experiments). **c**, End of study mouse plasma PK profile from OVCAR-3 CDX tumor model. Mice were administered a p.o. dose of AM-9022 (30 mg per kg) for 18 d. Scatterplot shows the mouse PK profile presented as mean plasma concentration versus time (*n* = 2 mice per time point) and AUC values (µM·h). **d**, Mitotic pH3 imaging analysis. JIMT-1 cells were treated for 24 h with DMSO, AM-0277, AM-1882, AM-5308, or AM-9022 at the indicated concentrations. Cells were stained to detect DNA and pH3. Graphs of compound concentration-response profiles presented as pH3 percentage and EC_50_ values (*n* = 2 independent experiments in duplicate). **e**, End of study mouse plasma PK profile from JIMT-1 CDX tumor model. Mice were administered a p.o. dose of AM-9022 (30 or 100 mg per kg) for 21 d. Scatterplot shows the mouse PK profile presented as mean plasma concentration versus time (*n* = 2 mice per time point) and AUC values (µM·h). Source data

We next sought to optimize our lead-series PK properties for favorable oral bioavailability and low clearance. Oral candidate AM-9022 dosed at 10 mg per kg achieved high plasma exposure in mice and showed double-digit nanomolar potency in the OVCAR-3 pH3 mitotic marker assay (Extended Data Fig. 8a). AM-9022 had similar inhibitory effects on the motor activity of human and mouse KIF18A (Extended Data Fig. 8b).

**Fig. 6:**
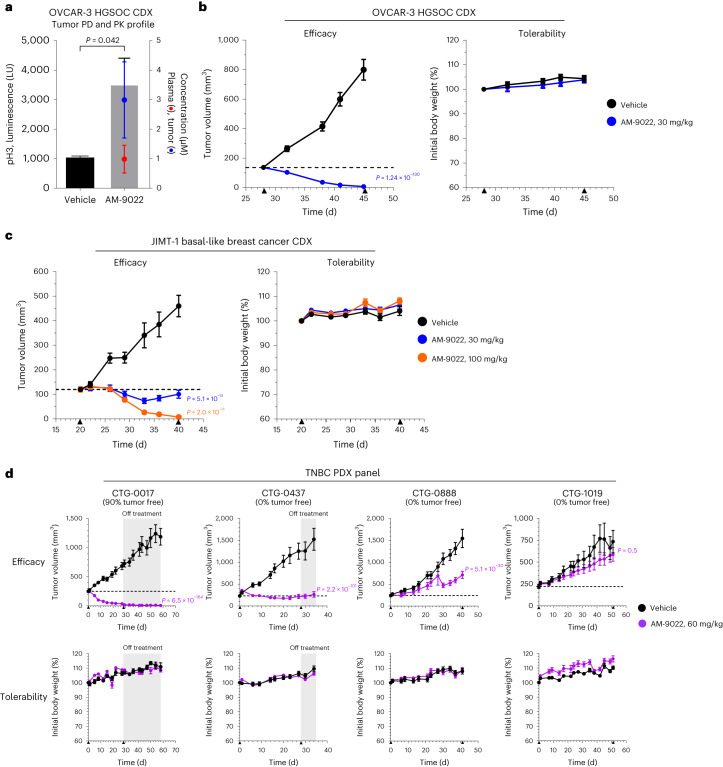
Oral candidate AM-9022 induces TR in vivo. **a**, PD and PK profiles of AM-9022 in the OVCAR-3 HGSOC CDX tumor model. Mice were administered a single oral (p.o.) dose of vehicle or AM-9022 (30 mg per kg). Tumor and blood were collected 24 h after treatment and analyzed for pH3 signal in tumor and compound PK in tumor and plasma. Graph shows pH3 luminescence signal with mean + s.d. (bar, left axis) and tumor (blue) and plasma (red) concentrations with mean ± s.d. (right axis) for each treatment group (*n* = 3 mice per group). Statistical significance was determined for AM-9022 relative to the vehicle by unpaired two-tailed *t*-test (Welch’s correction) and is shown as *P* values. **b**, AM-9022 efficacy and tolerability analysis in the OVCAR-3 HGSOC CDX tumor model. Mice were administered a p.o. dose of vehicle or AM-9022 (30 mg per kg) daily for 18 d. Graphs show tumor volume and mouse body weight measurements as mean ± s.e.m. versus time (d) (*n* = 10 mice per group); the dashed line indicates 100% TGI. Treatment start and stop (▴) are indicated on the *x* axis. **c**, AM-9022 efficacy and tolerability analysis in the JIMT-1 basal-like breast cancer CDX tumor model. Mice were administered a p.o. dose of vehicle or AM-9022 (30 or 100 mg per kg) daily for 21 d. Graphs show tumor volume and mouse body weight measurements as mean ± s.e.m. versus time (d) (*n* = 10 mice per group); the dashed line indicates 100% TGI. Treatment start and stop (▴) are indicated on the *x* axis. **d**, AM-9022 efficacy and tolerability analysis in four low-passage TNBC PDX tumor models (CTG-0017, CTG-0437, CTG-0888, CTG-1019). Mice were administered a p.o. dose of vehicle or AM-9022 (60 mg per kg) daily for ≥28 consecutive days. Graphs show tumor volume (top) and mouse body weight (bottom) measurements as mean ± s.e.m. versus time (d) (*n* = 10 mice per group); dashed lines indicate 100% TGI. Treatment start and stop (▴) are indicated on the *x* axis. After the cessation of treatment (shaded gray area), tumor durability analysis was performed for CTG-0017 and CTG-0437 models until day 58 and day 34, respectively. Mice with no measurable tumor are indicated as tumor free. Statistical significance was determined for tumor efficacy for the treatment group(s) relative to the vehicle by the linear mixed-effect analysis model with Dunnett’s multiplicity adjustment and is shown as *P* values. See supporting data (Extended Data Fig. 8 and Supplementary Table 6). Source data

To investigate the PD effects of our oral candidate, mice with established OVCAR-3 CDX tumors were administered vehicle or AM-9022 at 30 mg per kg. Tumor and blood samples were collected 24 h after treatment for pH3 PD and PK analysis. AM-9022 increased pH3 mitotic marker levels in OVCAR-3 tumors by 3.4-fold (*P* value = 0.042), with higher compound exposure in tumors relative to plasma (Fig. 6a). To evaluate the efficacy and tolerability of our oral candidate, mice with established OVCAR-3 tumors were administered vehicle or AM-9022 at 30 mg per kg daily for 18 d. Promisingly, AM-9022 inhibited tumor growth (*P* values = 1.24 × 10^−130^), with evidence of TR (95% TR, six of ten tumor-free mice) and no body weight loss (Fig. 6b). At the end of the study, the plasma AUC value for AM-9022 was 53 µM ·h (Extended Data Fig. 8c).

We next examined the activity of AM-9022 in the JIMT-1 breast cancer CDX tumor model. In vitro studies confirmed that our KIF18A inhibitors were active against JIMT-1 cells (Supplementary Table 3 and Extended Data Fig. 8d). Mice with established JIMT-1 tumors were administered vehicle or AM-9022 at 30 and 100 mg per kg daily for 21 d. AM-9022 administered at 30 and 100 mg per kg inhibited tumor growth (*P* values ≤ 5.1 × 10^−13^) with evidence of TR (16% and 94% TR, respectively) at well-tolerated doses (Fig. 6c). At the end of the study, the plasma AUC value for AM-9022 at 30 mg per kg was 3.7-fold higher in the JIMT-1 model relative to the OVCAR-3 model (Extended Data Fig. 8c,e).

Finally, we evaluated the activity of AM-9022 in four low-passage TNBC patient-derived xenograft (PDX) tumor models (CTG-0017, CTG-0437, CTG-0888, CTG-1019) that harbored *TP53* mutations plus a subset of relevant cancer gene alterations (Supplementary Table 6). Mice with established PDX tumors were administered vehicle or AM-9022 at 60 mg per kg daily for ≥28 d. The duration of treatment beyond 28 d depended on the level of anti-cancer activity and whether the tumors reached a predetermined size cutoff. We included an observation phase after treatment cessation for CTG-0017 and CTG-0437 models to assess durability and tumor regrowth. The pattern of sensitivity to AM-9022 treatment varied across the TNBC PDX panel (Fig. 6d). Notably, AM-9022 inhibited tumor growth in the CTG-0017 model (*P* value = 6.5 × 10^−164^) with evidence of TR (83% TR), resulting in 90% tumor-free mice by day 58. AM-9022 inhibited tumor growth in the CTG-0437 model (101% TGI, *P* value = 2.2 × 10^−101^). AM-9022 was less effective in the CTG-0888 model (63% TGI, *P* value = 5.1 × 10^−31^) and had no anti-cancer effects in the CTG-1019 model (Fig. 6d). As before, inhibition of KIF18A activity with AM-9022 was well tolerated by the mice (Fig. 6d). Together, these data show that the oral candidate AM-9022 has significant anti-cancer effects in five of six human breast and ovarian tumor models at well-tolerated doses, resulting in TR or stasis in OVCAR-3, JIMT-1, CTG-0017 and CTG-0437 models.

## Discussion

Attempts to improve anti-mitotic drugs through the development of small-molecule inhibitors of essential mitotic kinases and kinesins have failed in clinical testing due to dose-limiting myelosuppression and a lack of efficacy^4,5^. To overcome this formidable barrier, we set out to discover targetable mitotic enzymes that selectively kill cancer cells with CIN features while largely sparing normal cells.

Here, we provide evidence that selective inhibition of the KIF18A motor has promising preclinical activity across a subset of human cancer models. We discovered a class of potent and selective KIF18A inhibitors that preferentially block MT-ATPase motor activity and are non-competitive with ATP and MTs. Our KIF18A inhibitors faithfully phenocopy the mitotic and viability effects observed with *KIF18A* KD in a panel of breast and ovarian cancer cell lines. Importantly, the sensitivity profile of AM-1882 aligns with *KIF18A*-KD and -KO gene dependency scores across cancer cell lines, suggesting that the mode of action of AM-1882 is chiefly driven by inhibition of KIF18A. Cancer cell lines with CIN features show preferential sensitivity to inhibition of KIF18A, leading to SAC activation, multipolarity, centrosome fragmentation and apoptosis. Additionally, inhibiting KIF18A results in phosphorylation of γH2AX and the formation of cGAS-positive MN in BT-549 cells. This provides a strong case for investigating the effects of KIF18A inhibition on pro-inflammatory signaling in CIN cancers^45^. Our KIF18A inhibitors have minimal detrimental effects on human normal somatic cells at concentrations that are well above the range required to kill cancer cells, distinct from other anti-mitotic agents. While KIF18A may have a role in some normal cell lineages or progenitor cell populations, the viability of *Kif18a*-KO mice suggests that its function is non-essential in most somatic cell types. In vivo, our KIF18A inhibitors showed significant PD and anti-cancer effects in multiple *TP53*-mutant CIN^+^ tumor models but had no anti-cancer effect on the *TP53*-WT CIN^−^ tumor model. The oral candidate AM-9022 demonstrated impressive TR or stasis in both CDX and PDX models. Equally pertinent, our KIF18A inhibitors were well tolerated by the mice, with no evidence of body weight loss. This favorable tolerability profile in mice suggests that KIF18A inhibitors may serve as a good combination partner for other therapies, such as PARP inhibitors.

*TP53* mutations were enriched in cancer cell lines sensitive to KIF18A inhibition, which suggests that inactivation of the p53 pathway may remove the barrier that guards against CIN cell expansion^46^. Our study found that a subset of breast and ovarian cancer cell lines with *CCNE1* amplification were sensitive to KIF18A inhibition but resistant to CDK4–CDK6 inhibition. This suggests that Rb pathway status may have utility as a predictive biomarker. We identified *BRCA1*-altered cell lines sensitive to KIF18A inhibition that showed enhanced apoptosis in combination with the PARP inhibitor olaparib. Understanding the impact of homologous recombination repair pathway status may help to guide further KIF18A-inhibitor studies in homologous recombination-proficient and -deficient settings^47^. Genetic alterations in *CCNE1* and *BRCA1* are mutually exclusive in HGSOC and TNBC tumors^42,43^, indicating that they represent distinct patient populations potentially targetable with a KIF18A inhibitor. Our KIF18A-inhibitor cellular profile is supported by a trio of recent studies showing CIN features as the common link discriminating *KIF18A* gene dependency^21–23^. Notably, the study by Ganem and colleagues^21^ showed that genetic loss of *KIF18A* reduced cell viability in WGD^+^ cancer cells. We extended this hypothesis to show that KIF18A-inhibitor sensitivity was significantly enriched in *TP53*-mutant breast and ovarian cancer cell lines with CIN features (WGD, ploidy, AS). A combination of these aforementioned features in conjunction with emerging CIN signatures may enrich for specific types of CIN tumors^48,49^.

In summary, we describe here the discovery and characterization of KIF18A inhibitors with promising anti-cancer activity. Our KIF18A inhibitors will serve as excellent tools to further explore CIN as a cancer-specific vulnerability and to address outstanding questions concerning KIF18A biology, SAC signaling and biomarker-discovery efforts. Lastly, from a therapeutic perspective, this study provides the framework to explore the potential of KIF18A inhibitors for targeting CIN-driven human cancers.

## Methods

### Ethics statement

All animal experimental protocols were approved by the Amgen Animal Care and Use Committee and were conducted in accordance with the guidelines set by the Association for Assessment and Accreditation of Laboratory Animal Care. Mice were housed in an environmentally controlled room (temperature, 23 ± 2 °C; relative humidity, 50% ± 20%) on a 12-h light–dark cycle. Mice were offered commercial rodent chow and water ad libitum. Mice with a tumor size exceeding 2,000 mm^3^ were removed from the study and euthanized.

### Cell lines

Please see the Nature Portfolio Reporting Summary linked to this article for cell line source and authentication information. Cell lines were cultured at 37 °C in an atmosphere of 5% CO_2_. The DNA-barcoded cancer cell line panel was established by the Broad Institute^40,41^. The cancer cell line used for in vivo studies tested negative for mycoplasma. Cancer cell line feature information is from public sources (https://depmap.org/portal, https://www.cbioportal.org, https://cellmodelpassports.sanger.ac.uk, https://tp53.isb-cgc.org/), the DepMap Consortium portal and published reports^50–53^.

### Compound sources

KIF18A compounds (AM-0277, AM-1882, AM-5308, AM-9022) were synthesized by Amgen. The following compounds were purchased as follows: AM-7710 (Enamine), BI-2536 (Jubilant Biosys), docetaxel (Accord Healthcare), doxorubicin (Sigma-Aldrich), gemcitabine (Zydus Hospira), GF120918 (Sigma-Aldrich), GSK923295 (Selleck), ispinesib (Selleck), nocodazole (Sigma-Aldrich), nutlin 3a (Cayman Chemical), olaparib (AstaTech), paclitaxel (Sigma-Aldrich), palbociclib (Sigma-Aldrich) and vincristine (Tocris).

### Statistics and reproducibility

Group analysis was performed using Student’s *t*-test or one-way ANOVA when homogeneity and normality assumptions held with Welch’s correction in case of lack of homogeneity. Additional statistical method information is provided in In vivo pharmacology. No statistical methodologies were used to predetermine the sample size. In vivo studies were performed using standard sample sizes for tumor PD (*n* = 3 mice per group) and tumor efficacy (*n* = 10 mice per group)^54^. Studies conducted by Amgen were unblinded. When possible, higher-throughput assays were repeated at least twice in independent experiments with similar results. The PRISM screen, western blotting and a subset of lower-throughput assays were performed once. Experimental run information is detailed in the figure legends, the Methods and the Source Data. Unless otherwise specified, all graphing, curve fitting (four-parameter non-linear regression equation) and statistical significance testing were performed using GraphPad Prism version 7.05+ software (GraphPad Software).

### Kinesin motor assays

#### ADP-Glo motor assays

Motor activity was assessed with the ADP-Glo luminescence assay (Promega) using assay conditions as described previously^28^. Recombinant truncated motor proteins were expressed and purified by Amgen (hKIF18A (1–467, 4 nM), mKIF18A (1–467, 4 nM), hKIF19A (1–463, 32 or 100 nM), hKIF18B (1–436, 8 nM), hKIFC1 (266–673, 4 nM)) or procured from Cytoskeleton (hEG5, 4 nM; hCENP-E, 8 nM). Compounds were assessed with 30 µM ATP and 30 µg ml^−1^ MTs and the motor protein concentrations indicated above; data are from two or four independent experiments. KIF18A compounds were assessed with or without MTs (0 or 30 µg ml^−1^) with hKIF18A (160 nM); data are from two independent experiments in duplicate. KIF18A compounds were assessed with 30 or 300 µM ATP and 5 or 80 µg ml^−1^ MTs for hKIF18A (4 nM); data are from one experiment. AM-7710-series analog MT-ATPase IC_50_ values were obtained from the Genedata Screener datastore at Amgen (Extended Data Fig. 2a).

#### Enzyme-linked inorganic phosphate motor assays

KIF18A compounds (1 µM) were assessed against a panel of motor proteins (hCENP-E, hEG5, hKIFC3, hKIF3C, human chromokinesin, hMCAK, hMKLP1, hMKLP2) using an enzyme-linked inorganic phosphate assay according to the manufacturer’s protocol (Cytoskeleton) and as described previously^28^. Data are from one or two independent experiments in duplicate or triplicate. Studies were conducted by Cytoskeleton.

### Kinome binding assay

KIF18A compounds (1 µM) were assessed against a panel of kinases (*n* = 96) using a competition binding assay as described previously^28,55^. Data are from one experiment. Studies were conducted by Eurofins DiscoverX.

### Tubulin-polymerization assays

The fluorescence-based tubulin-polymerization assay was performed according to the manufacturer’s protocol (Cytoskeleton) with DMSO, KIF18A compounds (10 µM), paclitaxel (5 µM) and nocodazole (5 µM). Tubulin polymerization was measured using the SpectraMax M5 plate reader (Molecular Devices) set to detect at 440 nm with one measurement per minute for 90 min at 37 °C. Data are from one or three independent experiments. Data are graphed as mean fluorescence intensity versus time with corresponding AUC values.

### Imaging assays

Please see the Nature Portfolio Reporting Summary linked to this article for antibody information. Unless otherwise specified, cells were fixed in 4% formaldehyde (Thermo Scientific).

#### Cell growth assays (small interfering RNA)

Cell lines (*n* = 9) were seeded in 96-well imaging plates (Corning). The next day, cells were treated for 96 h with 10 nM individual siRNA species (*KIF18A* (*n* = 7), *EG5* (*n* = 2), NTC (*n* = 9); details are in Supplementary Table 1) and 0.3 µl Lipofectamine RNAiMAX (Invitrogen) according to the manufacturer’s protocol. Data are from two independent experiments in duplicate. Fixed cells were washed with PBS and stained with Hoechst 33342 (Invitrogen) in wash buffer (PBS, 1% BSA, 0.2% Triton X-100 (Sigma)). Plates were imaged with an ArrayScan VTI HCS Reader (Thermo Scientific) with a ×10 objective. The total number of nuclear objects (mean object area ± 3 s.d. based on the DMSO control) was counted from the same number of fields per well. Data were graphed for individual siRNA species, and group means are given for cell counts. Statistical significance was determined for *KIF18A* versus NTC siRNA groups by unpaired two-tailed *t*-test at a significance level of 0.05 with Welch’s correction as appropriate.

#### Cell growth assays (compound)

Cell growth assays were performed as described previously^28^. Cell lines (*n* = 11) were treated for 96 h with DMSO or AM-0277, AM-1882, AM-9022 and palbociclib (maximum concentration of 6 µM) or ispinesib (maximum concentration of 0.6 µM) over a 19-point or 17-point concentration range. Data are from two independent experiments in duplicate. Imaging data were collected from the same number of fields per well. The count POC value was computed using the formula (count POC = (compound-treated nuclear count) ÷ (DMSO-treated nuclear count) × 100). If the maximal response was <50% at 6 µM, the cell line was considered insensitive. A mean count EC_50_ value was determined for the sensitive cell line group. For OVCAR-8 and OVCAR-8 ADR^RES^ cell growth analysis, cells were treated for 96 h with AM-1882 and AM-9022 (maximum concentration of 6 µM) or paclitaxel and doxorubicin (maximum concentration of 1 µM) over a 19-point concentration range, with or without P-gp-inhibitor GF120918 (1 µM). OVCAR-8 cell lines were evaluated with or without P-gp inhibitor in two independent experiments. Data were graphed as a concentration–response profile with corresponding count EC_50_ values.

#### Mitotic assays (small interfering RNA)

Cell lines (*n* = 8) were seeded in 96-well imaging plates. The next day, cells were treated for 48 h with 10 nM individual siRNA species (*KIF18A*, *n* = 5; NTC, *n* = 4; details are in Supplementary Table 1) and 0.3 µl Lipofectamine RNAiMax according to the manufacturer’s protocol. The assay was performed with four to eight replicate wells per siRNA. Data are from two independent experiments (MDA-MB-157, CAL-51, OVCAR-3) or one experiment (BT-549, HCC-1806, MCF-7, MDA-MB-453, HMEC). Fixed cells were washed twice and incubated in wash buffer with horse serum (four drops per 10 ml) (Vector Labs) overnight at 4 °C. Cells were stained in wash buffer with anti-pH3 (05-806, Millipore) and anti-pericentrin (Ab4448, Abcam) antibodies for 2 h at room temperature. Cells were washed twice and stained with secondary antibodies (anti-mouse IgG Alexa Fluor 568 (A-11004, Invitrogen), anti-rabbit IgG Alexa Fluor 488 (A11034, Invitrogen)) for 1 h at room temperature. Cells were washed twice and counterstained with 6-diamidino-2-phenylindole (DAPI; Calbiochem). Plates were imaged with an iCys laser scanning cytometer with a ×40 objective running iGeneration version 7 software (CompuCyte). The segmentation scheme included (1) integral versus maximum pixel (DAPI) to establish DNA content profiles, (2) integral (pH3) versus integral (DAPI) to gate on the pH3^+^ mitotic cell population and (3) a histogram of pH3^+^ PCM focus count (pericentrin) scored as ≤2 PCM foci (blue, R6 region) or >2 PCM foci (red, R5 region) (Extended Data Fig. 1b). A representative 5 × 5 image gallery was generated for MDA-MB-157 and CAL-51 pH3^+^ cells showing DNA and PCM staining features for *KIF18A* (siRNA ID = h*KIF18A*_5) and NTC (siRNA ID = NTC_1) siRNA species. Data were graphed for individual siRNA species, and group means are given for pH3^+^ and >2 PCM focus count percentages. Statistical significance was determined for *KIF18A* versus NTC siRNA groups by unpaired two-tailed *t*-test at a significance level of 0.05 with Welch’s correction as appropriate.

#### Mitotic assay (CRISPR RNA)

MDA-MB-157 Cas9 cells were seeded in 96-well imaging plates. The next day, cells were treated for 48 h with 25 nM tracrRNA plus 25 nM individual CRISPR RNA (crRNA) species (*KIF18A*, *n* = 5; *EG5*, *n* = 5; NTC, *n* = 5; details are in Supplementary Table 2) and 0.2 µl DharmaFECT 1 according to the manufacturer’s protocol (Dharmacon). Cells were also treated with matched pooled crRNA species. Data are from two independent experiments in triplicate. Cells were fixed and stained as described above with the following modifications. Cells were stained with secondary antibodies (anti-mouse IgG Alexa Fluor 647 (A-21235, Invitrogen), anti-rabbit IgG Alexa Fluor 488 (A11034, Invitrogen)) and counterstained with Hoechst 33342. Plates were imaged with an ArrayScan VTI HCS Reader with a ×20 objective. The segmentation scheme included (1) compute nuclear counts (mean object area ± 3 s.d. of NTC), (2) compute pH3^+^ count and (3) compute PCM foci for each pH3^+^ cell and score as ≤2 or >2 PCM foci. Imaging data were collected from the same number of fields per well. Data were graphed for individual and pooled crRNA species, and group means are given for pH3^+^ and >2 PCM foci percentages. Statistical significance was determined for *KIF18A* versus NTC crRNA groups by unpaired two-tailed *t*-test at a significance level of 0.05 with Welch’s correction as appropriate.

#### Mitotic assays (compound)

Mitotic assays were performed on MDA-MB-157, OVCAR-3 and JIMT-1 cell lines as described previously^28^. Cells were treated for 24 h with DMSO or KIF18A compounds (maximum concentration of 5 or 6 µM) over a 20-point to 17-point concentration range. Data are from at least two independent experiments in duplicate. Plates were imaged with an ArrayScan VTI HCS Reader with a ×10 objective (pH3 alone) or a ×20 objective (pH3 and PCM foci). Imaging data were collected from the same number of fields per well. Data were graphed as a concentration–response profile with corresponding pH3^+^ or >2 PCM foci EC_50_ values. AM-7710-series analog mitotic EC_50_ values were obtained from the Genedata Screener datastore at Amgen (Extended Data Fig. 2a).

#### Multiparametric cell assays (compound)

Human foreskin fibroblasts were seeded in 96-well imaging plates. The next day, cells were treated (in duplicate plates) for 48 h with DMSO or compound ((AM-0277, nutlin 3a, maximum concentration of 10 µM), (AM-1882, BI-2536, ispinesib, paclitaxel, maximum concentration of 1 µM), (palbociclib, GSK923295, maximum concentration of 5 µM)) over a nine-point concentration range. Data are from one experiment. In the first plate, cells were pulsed with BrdU (Invitrogen) for 3 h before fixation and then treated with acid and neutralized in wash buffer. Cells were blocked in wash buffer with horse serum overnight at 4 °C. The first plate was stained with anti-BrdU Alexa Fluor 647 (B35140, Invitrogen) and anti-p21 (2947, Cell Signaling) antibodies for 2 h at room temperature. Cells were washed twice and stained with secondary anti-rabbit IgG Alexa Fluor 488 (A11034, Invitrogen) antibody for 1 h at room temperature. In the second plate, cells were fixed, washed, blocked overnight at 4 °C and stained with anti-cl-PARP (44-6986, Invitrogen) and anti-phospho-γH2AX (serine 139) (05-636, Millipore) antibodies for 2 h at room temperature. Cells were washed twice and stained with secondary antibodies (anti-rabbit IgG Alexa Fluor 647 (A21245, Invitrogen), anti-mouse IgG Alexa Fluor 488 (A11029, Invitrogen)) for 1 h at room temperature. Cells were washed twice and counterstained with Hoechst 33342. Plates were imaged with an ArrayScan VTI HCS Reader with a ×20 objective. Imaging data were collected from the same number of fields per well. Nuclear counts (as described above) and the percentage of BrdU (DNA synthesis)-, cl-PARP (apoptosis)-, phospho-γH2AX (DNA damage)- and p21 (cell arrest)-positive objects was graphed as concentration–response heatmaps.

#### KIF18A and centrin 3 staining (compound)

HeLa cells were seeded in two-well glass chamber slides (Lab-Tek) and cultured for 2 d. Cells were treated for 6 h with DMSO or AM-1882 (0.05 µM). Fixed cells were washed and incubated in 90% ice-cold methanol, washed again and incubated in wash buffer with horse serum overnight at 4 °C. Cells were stained with anti-KIF18A (A301-080A, Bethyl) and anti-centrin 3 (H00001070, Abnova) antibodies for 2 h at room temperature. Cells were washed twice and stained with secondary antibodies (anti-rabbit-IgG Alexa Fluor 568 (A11036, Invitrogen), anti-mouse-IgG Alexa Fluor 488 (A11029, Invitrogen)) for 1 h at room temperature. Cells were washed twice and counterstained with DAPI. ProLong antifade (Invitrogen) was added before mounting the coverslips. Slides were imaged with an Eclipse Ni-E fluorescence microscope with a ×60 objective running Elements software (Nikon). Data are from one experiment. Line scan analysis was performed on three mitotic cells by aligning the line tool end to end on the bi-orientated centrosome centers. Line scan intensity data were collected and graphed for DAPI, KIF18A and centrin 3 channels.

#### Pericentrin and centrin 3 staining (compound)

CAL-51 and MDA-MB-157 cells were seeded in 96-well imaging plates (PerkinElmer). The next day, cells were treated for 24 h with DMSO, AM-0277 (0.5 µM) or AM-1882 (0.05 µM). Fixed cells were washed three times with 0.1% Triton X-100 in PBS, and cells were stained with anti-pericentrin (Ab4448, Abcam) and anti-centrin 3 (H00001070, Abnova) antibodies overnight at 4 °C. Cells were washed twice and stained with secondary antibodies (anti-mouse IgG Alexa Fluor 647 (A-21235, Invitrogen), anti-rabbit IgG Alexa Fluor 488 (A11034, Invitrogen)) for 2 h at room temperature. Cells were washed twice and counterstained with Hoechst 33342. Plates were imaged with a confocal UltraVIEW VoX fluorescence microscope with a ×60 objective running Volocity software (PerkinElmer). Data are from one experiment. Representative maximum projection images were captured for each treatment condition.

#### Pericentrin and α-tubulin staining (compound)

MDA-MB-157 cells were seeded in two-well glass chamber slides (Lab-Tek) and cultured for 2 d. Cells were treated for 24 h with DMSO, AM-1882 (0.2 µM) or AM-5308 (0.5 µM). Fixation, staining and imaging were performed as described previously^28^. Data are from one experiment. Representative images were captured for each treatment condition.

#### Phospho-γH2AX and cGAS staining (compound)

BT-549 cells were seeded in four-well glass chamber slides and cultured for 2 d. Cells were treated for 48 h with DMSO or AM-1882 (0.2 µM). Fixed cells were washed and incubated in wash buffer with horse serum overnight at 4 °C. Cells were stained with anti-phospho-γH2AX (serine 139) (05-636, Millipore) and anti-cGAS (15102, Cell Signaling) antibodies for 2 h at room temperature. Cells were washed twice and stained with secondary antibodies (anti-mouse IgG Alexa Fluor 568 (A-11004, Invitrogen), anti-rabbit IgG Alexa Fluor 488 (A11034, Invitrogen)) for 1 h at room temperature. Cells were washed twice and counterstained with DAPI. ProLong antifade was added before mounting the coverslips. Slides were imaged with an Eclipse Ni-E fluorescence microscope with a ×40 objective running Elements software. Data are from one experiment. Representative images were captured for each treatment condition.

#### Time-lapse mitotic cell fate assay (compound)

HeLa Kyoto cells expressing α-tubulin–EGFP and H2B–mCherry proteins were seeded in six-well plates. Cells were synchronized in G1S by double-thymidine block using 2 mM thymidine (Sigma). Cells were released from the G1S block in growth medium containing DMSO or AM-1882 (0.2 µM). Plates were imaged with an Eclipse TE2000-E fluorescence microscope with a ×20 objective running Elements software (Nikon) in a temperature- and CO_2_-controlled chamber. Time-lapse was initiated after G1S release, and images were captured every 15 min for 48 h for brightfield, EGFP and mCherry channels. Analysis was performed on cells entering first mitosis (*n* = 40 cells per group), and cells were scored for time in mitosis and cell fate (complete cell division or death in mitosis). Cells exiting mitosis were scored for the number of daughter cells and early-interphase cell death. Data are from one experiment. Data are graphed as time in mitosis and cell fate grouping for each treatment condition.

### Sensory neuron differentiation and neurite outgrowth assay

Please see the Nature Portfolio Reporting Summary linked to this article for defined medium details.

#### Sensory neuron differentiation

The neural crest cell-induction protocol was modified from previously described methods^56^. hiPSCs were cultured in E8F defined medium. On days 0 and 1, the medium was changed to SBCHIR defined medium. On day 2, the medium was changed to DMHB defined medium. On day 10, hiPSC-derived neural crest cells were washed twice with PBS and incubated with Accutase (Stemcell Technologies) for 7 min and then cryopreserved. hiPSC-derived neural crest cells were thawed and cultured in NC defined medium with Y-27632 (10 µM). On day 11, the medium was changed to SNP defined medium. On day 13, hiPSC-derived sensory neuronal precursor cells were washed twice with PBS and incubated with Accumax solution (ICT) for 20 min and then cryopreserved. hiPSC-derived sensory neuronal precursors were thawed and seeded in Nunclon Sphera 96-well plates (Thermo Fisher) in sensory neurosphere-induction defined medium with Y-27632 (only on day 0). On day 3, the medium was changed to sensory neurosphere-maintenance defined medium, and sensory neurosphere-maintenance defined medium was carefully changed three times per week until day 14.

#### Neurite outgrowth imaging assay

On day 13, PhenoPlate 96-well plates (PerkinElmer) were coated with poly-l-ornithine (Sigma) for 1 h at 37 °C, washed with sterile water and incubated with Matrigel solution (Corning) in DMEM/F12 medium overnight. On day 14, Matrigel solution was aspirated, and 350 µl BrainPhys medium was added to each well. Sensory neurospheres were carefully transferred to prepared PhenoPlate 96-well plates. After 4 h, the neurospheres stabilized, and 250 µl BrainPhys medium was carefully removed from each well. One hundred microliters of neurite outgrowth defined medium was added to each well, and the plates were incubated overnight at 37 °C. On day 15, 50 µl of medium was removed from each well and replaced with 50 µl of fresh neurite outgrowth defined medium containing DMSO or AM-1882, AM-5308, ispinesib, GSK923295, vincristine and paclitaxel over a five-point concentration range (0.001–10 µM). After 24 h, cells were fixed and washed three times with 0.1% Triton X-100 in PBS and incubated in buffer (PBS, 5% goat serum, 0.1% Tween-20) for 1 h at room temperature. Cells were stained with anti-β3-tubulin antibody (801201, TUJ1, BioLegend) overnight at 4 °C. Cells were washed three times and stained with secondary antibody (anti-mouse IgG Alexa Fluor 488 (115-545-206, Jackson ImmunoResearch)) for 2 h at room temperature. Cells were washed twice and counterstained with DAPI. Plates were imaged with the Opera Phenix HCS System (PerkinElmer). Plates were scanned with a ×20 objective to quantify neurosphere fluorescence signal for β3-tubulin (neurite area) and DAPI (nuclear area). Columbus Image Analysis software (PerkinElmer) was used to measure β3-tubulin- and DAPI-positive areas. The β3-tubulin-positive area was calculated as (β3-tubulin-positive neurite area = total β3-tubulin area − DAPI area). Representative images of neurite outgrowth were captured for a subset of treatment conditions. Data are from two independent experiments in two or three replicates. Data were graphed as total neurite area (µm^2^) per neurosphere for each treatment condition.

### Colony-formation and cell growth assays (compound)

For the single-agent studies, KIF18A-inhibitor-sensitive cell lines were seeded in 100-mm dishes. The next day, cells were treated with DMSO or AM-0277 (0.5 µM) for 6 d. MCF-7 cells were treated with palbociclib (1.0 µM) for 6 d as a cytostatic control. One set of dishes was fixed and stained with crystal violet dye (Sigma), washed repeatedly with water and imaged with a digital scanner (Hewlett Packard). Cells from a second set of dishes were collected and counted with a Vi-CELL XR Analyzer (Beckman Coulter), and data were graphed as cell counts. An equal number of cells from each treatment were seeded in new dishes in drug-free growth medium and cultured until cells previously treated with DMSO reached confluence (7–9 d). Cells in dishes were fixed and stained with crystal violet dye as described above. Data are from one experiment.

For the combination studies, HCC-1937 and OVCAR-8 cells were seeded in 12-well plates. The next day, HCC-1937 cells were treated for 6 d with DMSO, AM-1882 (0.007, 0.01, 0.015 µM) or olaparib (8.0, 25 µM) in a 3 × 2 combination matrix. OVCAR-8 cells were treated for 4 d with DMSO, AM-1882 (0.02, 0.045, 0.07 µM) or olaparib (1.5, 5.0 µM) in a 3 × 2 combination matrix. After treatment, cells were collected in 1 ml of trypsin–EDTA medium (Thermo Fisher), and 0.1 ml of cells was replated in drug-free growth medium and cultured until cells previously treated with DMSO reached confluence (8 d for HCC-1937, 4 d for OVCAR-8). Data are from one experiment. Plates were stained and imaged as described above.

### Flow cytometry assays

#### Cell cycle and cell growth assays (compound)

Human bone marrow mononuclear cells from four normal donors were expanded for 8 d as described previously^57^. Cells were seeded in 24-well plates and treated with DMSO or KIF18A compounds (1 µM), ispinesib (0.05 µM), paclitaxel (0.1 µM) and palbociclib (1 µM). AM-5308 and AM-9022 were assessed in two donors; the other test compounds were assessed in four donors in independent experiments. Separate plates were collected at 48 h (cell cycle) or 96 h (cell growth). The first set of plates was pulsed with BrdU for 2 h and processed as described previously^57^. Cells were analyzed with a BD LSRFortessa flow cytometer running FACSDiva software, and post-acquisition analysis was performed with FSC Express software. Cells were collected and counted from the second set of plates with the Vi-CELL XR Analyzer. Data were graphed for cell cycle (BrdU, sub-G1) and cell growth (count). Statistical significance was determined for each group relative to the DMSO control by one-way ANOVA at a significance level of 0.05, followed by Dunnett’s multiplicity adjustment.

#### BrdU-incorporation analysis (compound)

HMECs were seeded in six-well plates. The next day, cells were treated for 48 h with DMSO, AM-0277 (maximum concentration of 10 µM), palbociclib (maximum concentration of 10 µM) or ispinesib (maximum concentration of 1 µM) over a six-point concentration range. Data are from two independent experiments. Cells were pulsed with BrdU for 3 h and processed as described previously^58^. Cells were analyzed with a BD LSRFortessa flow cytometer running FACSDiva software, and post-acquisition analysis was performed using FSC Express software. Data were graphed as the BrdU-incorporation percentage for each treatment condition.

#### P-glycoprotein analysis

An asynchronous culture of OVCAR-8 parental and OVCAR-8 ADR^RES^ cells was collected, washed with PBS with 1% BSA and stained with anti-human CD243 (P-gp) APC (348608, BioLegend) or isotype APC control (400220, BioLegend) antibody for 45 min on ice. Cells were washed twice and stained with PO-PRO-1 Iodide (Invitrogen) to exclude dead cells. Cells were analyzed with a BD LSRFortessa flow cytometer running FACSDiva software, and post-acquisition analysis was performed using FSC Express software.

### Western blot analysis

Please see the Nature Portfolio Reporting Summary linked to this article for antibody information.

#### Cell lysate preparation and protein detection

Cell lysates were prepared from a single experiment by combining non-adherent and adherent cell fractions using RIPA buffer (Sigma) or the Minute Total Protein Extraction Kit (Invent Biotech), supplemented with protease and phosphatase inhibitors (Roche). Protein concentrations were determined using the Bradford assay (Bio-Rad). Proteins were resolved on Tris–glycine polyacrylamide gels (Invitrogen) and transferred to PVDF membranes (Bio-Rad). Membranes were incubated in blocking buffer (PBS, 0.5% Tween-20, 5% dry milk) with horse or goat serum (Vector Labs) for 60 min at room temperature on a shaker. Antibodies were added to blocking buffer, and samples were incubated for 2 h at room temperature or overnight at 4 °C on a shaker. Membranes were washed three times, followed by secondary antibody incubation using the VECTASTAIN ABC Kit (PK-4002 (mouse), PK-4001 (rabbit), Vector Labs). Immunoblot protein detection was performed with the Western Lightning Chemiluminescence reagent (PerkinElmer), and blue autoradiography film was developed (USA Scientific). Immunoblot films were scanned with a digital scanner (Hewlett Packard). β-actin or GAPDH loading controls were included in every experiment. Immunoblot proteins were quantified with the volume-measurement rectangle tool by Image Lab 6.1 software (Bio-Rad). The volume intensity measurement for each protein band of interest was normalized to that of the loading control (Supplementary Table 7).

#### Motor knockdown western blot analysis (small interfering RNA)

Cell lines were seeded in six-well plates. The next day, cells were treated for 48 h with 10 nM individual *KIF18A*, *EG5* or NTC siRNA species (details are in Supplementary Table 1) and 5 µl Lipofectamine RNAiMax according to the manufacturer’s protocol. Cell lysates were prepared from duplicate wells using RIPA buffer and processed for WBA as described above. HeLa cells were treated for 16 h with nocodazole (0.1 µg ml^−1^) as a mitotic control (Extended Data Fig. 1a). Antibodies included those specific for KIF18A, EG5, cl-PARP, MCL-1, cyclin B1 and β-actin.

#### Baseline western blot analysis

Cancer cell lines (*n* = 10) were seeded in six-well plates. Cell lysate was prepared at ∼70% confluency from duplicate wells using RIPA buffer and processed for WBA as described above. Antibodies included those specific for securin, cyclin B1, KIF18A, cyclin E1, total Rb, phospho-Rb, p16, p21 and GAPDH.

#### Apoptosis western blot analysis (compound)

Cancer cell lines (*n* = 12) were seeded in six-well plates. The next day, cells were treated for 48 h with DMSO, AM-0277 (0.5 µM) or AM-1882 (0.1 µM). Cell lysates were prepared from duplicate wells using RIPA buffer and processed for WBA as described above. Cell lysates were prepared from HCC-1806 cells treated with ispinesib (0.05 µM) and included on each immunoblot as a control. For PARP-inhibitor combination studies, HCC-1937 and OVCAR-8 cells were seeded in six-well plates. The next day, HCC-1937 cells were treated with DMSO, AM-1882 (0.01 µM), olaparib (20 µM) or in combination and OVCAR-8 parental cells were treated with DMSO, AM-1882 (0.03 µM), olaparib (5.0 µM) or in combination. After 48 h, cell lysates were prepared from duplicate wells using RIPA buffer and processed for WBA as described above. Antibodies included those specific for cl-PARP and GAPDH.

#### G1S block-release western blot analysis (compound)

OVCAR-3 cells were seeded in 100-mm dishes. Cells were synchronized in G1S by double-thymidine block using 2 mM thymidine. Cells were released from the G1S block in growth medium containing DMSO or AM-0277 (0.5 µM). Cells were collected at 4, 8, 10, 12, 14 and 24 h after G1S release. As a control, asynchronous OVCAR-3 cells were treated with DMSO or AM-0277 (0.5 µM) for 24 h. Cell lysates were prepared using RIPA buffer and processed for WBA as described above. Antibodies included those specific for cl-PARP, cyclin B1, BUBR1, KIF18A, MCL-1, cyclin E1 and β-actin.

#### Phospho-γH2AX western blot analysis (compound)

BT-549 cells were seeded in six-well plates. Plated cells were positioned on the IncuCyte ZOOM imager (Sartorius) in an incubator to monitor live-cell growth; images were captured with a ×10 objective every 4 h. The next day, cells were treated for 48 h with DMSO, AM-0277 (0.5 µM), AM-1882 (0.1 µM) or ispinesib (0.05 µM). Plates were removed from the imager, and cell lysates were prepared using the Minute Total Protein Extraction Kit and processed for WBA as described above. Data were graphed as mean confluency percentage versus time in triplicate. Antibodies included those specific for phospho-γH2AX and GAPDH.

#### BRCA1 western blot analysis

Cancer cell lines (*n* = 5) were seeded in six-well plates. Cell lysates were prepared at ∼70% confluency from duplicate wells using RIPA buffer. As a control, CAL-51 cells were treated for 48 h with 50 nM pooled *BRCA1* or NTC siRNA species (details are in Supplementary Table 1) and 5 µl Lipofectamine RNAiMax according to the manufacturer’s protocol. Cell lysates were prepared using RIPA buffer and processed for WBA as described above. Antibodies included those specific for BRCA1 (N terminal and C terminal) and GAPDH.

### Analysis of 3H-thymidine incorporation

Labeled CD3 microbeads were used to isolate human CD3^+^ T cells from two normal donors according to the manufacturer’s protocol (Miltenyi). Purified T cells were stimulated with anti-CD3 and anti-CD28 antibody-coated Dynabeads (Invitrogen) according to the manufacturer’s protocol and incubated for 24 h at 37 °C. Stimulated T cells were treated for 48 h with DMSO or AM-1882, AM-0277, ispinesib or palbociclib over a ten-point concentration range in triplicate. Cells were pulsed with 1 µCi of 3H-thymidine per well for 6 h at 37 °C. Labeled cells were transferred to glass fiber filters, and 3H-thymidine incorporation was measured with the MicroBeta plate reader (PerkinElmer). The 3H-thymidine-incorporation-counts-per-minute values were normalized to those of the DMSO control (POC). Data were graphed as concentration–response profiles with corresponding 3H-thymidine-incorporation EC_50_ values.

### Screen for profiling relative inhibition simultaneously in mixtures

AM-1882 was screened in the PRISM DNA-barcoded cancer cell line collection established by the Broad Institute^40,41^. Cell line pools were treated for 5 d with AM-1882 over an eight-point concentration range (maximum concentration of 2.5 µM) in triplicate. Curve fitting and AUC value determination were performed for AM-1882 as described previously^40,41^. Cell lines were selected with successful curve fits (*n* = 631) for association analysis performed using Cancer Dependency Map Consortium (DMC) custom tools (dataset release 20Q2+). Kuramochi cells were excluded from our analysis due to disparate AM-1882 effects on cell viability between DNA-barcoded and nonbarcoded screens. Cell lines with AM-1882 AUC values ≤ 0.65 (representing the lower quartile) were scored as sensitive to KIF18A inhibitor (Supplementary Table 3). Pan-cancer data were graphed as an AM-1882 concentration–response heatmap or as AM-1882 AUC versus tumor type (*n* = 24) in a violin plot. Pan-cancer AM-1882 AUC scores were correlated (Pearson) with gene dependency scores using RNAi KD or CRISPR KO datasets. Correlation values and associated *P* values and/or *q* values for gene dependencies were graphed in a volcano plot. Pan-cancer AM-1882 AUC values were correlated with gene mutation status; *TP53* gene status was classified as a hotspot mutation (missense, frameshift, nonsense, splice site) or other (WT, null). Analysis was performed specifically on breast and ovarian cancer cell lines (*n* = 58). AM-1882 AUC values were correlated with WGD status, DNA ploidy (≤2.1 or >2.1), AS (≤8 or >8), breast cancer subtypes (ER^−^HER2^−^, ER^−^HER2^+^, ER^+^HER2^+^, ER^+^HER2^−^), ovarian cancer subtypes (clear cell, endometrioid, HGSOC, other), DNA gene alterations (*TP53*, *CCNE1*, *RB1*, *BRCA1*), promoter methylation (*BRCA1*) and RNA expression (Supplementary Tables 3–5). Data were graphed for AM-1882 AUC values and the above-mentioned cell features. Statistical significance was determined for *TP53* status, CIN features and *BRCA1* status by unpaired two-tailed *t*-test at a significance level of 0.05 with Welch’s correction as appropriate.

### In vivo pharmacology

Female mice (athymic nude, CB.17 SCID) were housed in sterilized filter-capped cages and maintained under aseptic and pathogen-free conditions. Mice were 6–8 weeks old at tumor implantation. OVCAR-3, CAL-51 and OVCAR-8 CDX studies were conducted with athymic nude mice at Amgen. The JIMT-1 CDX study was conducted with CB.17 SCID mice at Charles River Laboratories. Four TNBC PDX studies were conducted with athymic nude mice at Champions Oncology. KIF18A compounds were formulated in 2% hydroxypropyl methylcellulose and 1% Tween-80 at pH 2.2 and stirred overnight before i.p. or oral (p.o.) administration. Docetaxel and gemcitabine were formulated in saline solution before i.p. administration.

Tumor volume was calculated as (length × width × height) and expressed in mm^3^. Tumor volume and body weight were measured twice per week using a digital caliper and an analytical laboratory scale, respectively. Tumor efficacy data were expressed as mean tumor volume ± s.e.m. for each group plotted as a function of time (d). Mice with no measurable tumor were classified as tumor free. Percent TGI was calculated as the difference between the mean change of tumor volume of a test group and the control group, using the formula:

$$\begin{array}{l}\textrm{Percent}\,{\textrm{TGI}}=\\100-\left(\frac{\left({\textrm{treated}}\,{\textrm{final}}\,{\textrm{volume}}-{\textrm{treated}}\,{\textrm{initial}}\,{\textrm{volume}}\right)}{\left({\textrm{control}}\,{\textrm{final}}\,{\textrm{volume}}-{\textrm{control}}\,{\textrm{initial}}\,{\textrm{volume}}\right)}\right)\times 100\end{array}$$

Statistical analysis was performed to evaluate the effect of treatment on tumor size over time relative to the vehicle control using a linear mixed-effect model implemented within the custom application IVEA using the R CRAN package. Dunnett’s correction was applied for multiplicity. Statistical significance was reported with *P* value < 0.05, otherwise considered not significant. Percent TR was calculated using the mean of initial and final tumor volumes within a test group, using the formula:

$$\textrm{Percent}\,{\textrm{regression}}=100-\left(\frac{{\textrm{treated}}\,{\textrm{final}}\,{\textrm{volume}}}{{\textrm{treated}}\,{\textrm{initial}}\,{\textrm{volume}}}\times 100\right)$$

#### OVCAR-3 tumor PD (pH3 immunoassay)

Tumor PD assays were performed as described previously^28^. Animals were randomized into treatment groups (*n* = 3 mice per group) based on similar tumor size and dosed with vehicle (i.p. or p.o.), AM-1882 (100 mg per kg, i.p.), AM-5308 (50 mg per kg, i.p.) or AM-9022 (30 mg per kg, p.o.). Tumor and blood plasma were collected 24 h after treatment and processed for PD (pH3) or PK (plasma, tumor) analysis. Data were graphed for tumor PD, plasma PK and tumor PK. Statistical significance was determined for AM-1882 and AM-5308 relative to vehicle by one-way ANOVA at a significance level of 0.05 with Dunnett’s multiplicity adjustment and for AM-9022 relative to vehicle by two-tailed *t*-test at a significance level of 0.05 with Welch’s correction.

#### OVCAR-3 tumor PD (pH3 imaging)

Mice were injected with OVCAR-3 cells (5.0 × 10^6^) subcutaneously in the right flank. Animals were randomized into treatment groups (*n* = 3 mice per group) based on similar tumor size and dosed with vehicle or AM-5308 (25 mg per kg, i.p.) for 2 consecutive days. Tumors were collected 24 h after treatment and processed for tumor imaging analysis. Formalin-fixed paraffin-embedded tumors were sectioned onto glass slides and deparaffinized, rehydrated and treated with citrate buffer and heat for antigen retrieval (Reveal Decloaker, Biocare Medical). Slides were blocked and stained in wash buffer with anti-α-tubulin (T6199, Sigma) and anti-pH3 (06-570, Millipore) antibodies overnight at 4 °C. Slides were washed twice and stained with secondary antibodies (anti-mouse IgG Alexa Fluor 488 (A11029, Invitrogen), anti-rabbit IgG Alexa Fluor 647 (A-21244, Invitrogen)) for 2 h at room temperature. Slides were washed twice and counterstained with DAPI. ProLong antifade was added before mounting the coverslips. Slides were imaged with a confocal UltraVIEW VoX fluorescence microscope running Volocity software (PerkinElmer). A primary scan was performed with a ×20 objective to select three regions of interest per tumor followed by enumerating pH3^+^ counts per area for each region. Representative maximum projection images were captured with a ×60 objective for DNA, α-tubulin and pH3 channels. Data were graphed for tumor PD. Statistical significance was determined for AM-5308 relative to the vehicle by unpaired two-tailed *t*-test at a significance level of 0.05 with Welch’s correction.

#### Cell line-derived xenograft tumor model efficacy (intraperitoneal dosing)

Mice were injected with OVCAR-3 cells (5.0 × 10^6^) subcutaneously in the right flank. Animals were randomized into treatment groups (*n* = 10 mice per group) based on equivalent tumor size and dosed i.p. with vehicle, AM-1882 (100 mg per kg) or AM-5308 (25 mg per kg) daily for 18 consecutive days or weekly with docetaxel (20 mg per kg). Plasma PK analysis was performed at 2, 4, 8, 16 and 24 h (*n* = 2 mice per time point). After the final dose on day 42, samples were collected for mouse blood count analysis (*n* = 6 mice per treatment group) by IDEXX BioResearch; platelet counts were not reported due to technical processing issues. Data were graphed for mouse blood counts (neutrophils, reticulocytes, red blood cells, lymphocytes and white blood cells). Statistical significance was determined for treatment groups relative to the vehicle by one-way ANOVA at a significance level of 0.05 with Dunnett’s multiplicity adjustment. Mice were injected with CAL-51 cells (5.0 × 10^6^) subcutaneously in the right flank. Animals were randomized into treatment groups (*n* = 10 mice per group) based on equivalent tumor size and dosed i.p. with vehicle, AM-1882 (100 mg per kg) or AM-5308 (25 mg per kg) daily for 18 consecutive days or twice weekly with gemcitabine (120 mg per kg). After the final dose on day 36, plasma PK analysis was performed as described above. Mice were injected with OVCAR-8 cells (5.0 × 10^6^) subcutaneously in the right flank. Animals were randomized into treatment groups (*n* = 10 mice per group) based on equivalent tumor size and dosed i.p. with vehicle, AM-1882 (50 or 100 mg per kg) or AM-5308 (25 or 50 mg per kg) daily for 18 consecutive days. After the final dose on day 46, blood was obtained by the retro-orbital method, and plasma PK analysis was performed as described above. After treatment cessation, tumor volumes and body weights were recorded until day 81. Mice with no measurable tumor on day 81 were classified as tumor free.

#### Cell line-derived xenograft tumor model efficacy (p.o. dosing)

Mice were injected with OVCAR-3 cells (5.0 × 10^6^) subcutaneously in the right flank. Animals were randomized into treatment groups (*n* = 10 mice per group) based on equivalent tumor size and dosed p.o. with vehicle or AM-9022 at 30 mg per kg daily for 18 consecutive days. After the final dose on day 45, plasma PK analysis was performed as described above. Mice with no measurable tumor on day 45 were classified as tumor free. Mice were injected with JIMT-1 cells (1.0 × 10^7^) subcutaneously in the right flank. Animals were randomized into treatment groups (*n* = 10 mice per group) based on equivalent tumor size and dosed p.o. with vehicle or AM-9022 (30 or 100 mg per kg) daily for 21 consecutive days. After the final dose on day 40, plasma PK analysis was performed as described above. Mice with no measurable tumor on day 40 were classified as tumor free.

#### Patient-derived xenograft tumor model efficacy (p.o. dosing)

Mice were implanted with low-passage PDX tumor fragments from each TNBC model (CTG-0017, CTG-0437, CTG-0888 and CTG-1019; details are in Supplementary Table 6). After tumor size reached 1,000–1,500 mm^3^, tumors were collected and tumor fragments were implanted subcutaneously in the left flank. Animals were assigned into treatment groups (*n* = 10 mice per group) based on equivalent tumor size and dosed p.o. with vehicle or AM-9022 (60 mg per kg) daily for ≥27 consecutive days. Study termination was set on a mean tumor size of 1,500 mm^3^ for the control group; dosing continued beyond day 28 if the tumor size threshold was not reached. CTG-0017 study dosing terminated on day 27, followed by a drug-free observation phase to day 58. CTG-0437 study dosing terminated on day 27, followed by a drug-free observation phase to day 34. Dosing on CTG-0888 and CTG-1019 terminated on day 43 (last measurement on day 41) and on day 52 (last measurement on day 51), respectively. Mice with no measurable tumor were classified as tumor free.

### Reporting summary

Further information on research design is available in the Nature Portfolio Reporting Summary linked to this article.

### Supplementary information

Reporting Summary

Supplementary Tables Supplementary Tables 1–7.

Supplementary Video 1 Time-lapse microscopy video of Kyoto HeLa cells treated with DMSO (file size, 19.9 MB).

Supplementary Video 2 Time-lapse microscopy video of Kyoto HeLa cells treated with AM-1882 (file size, 19.9 MB).

### Source data

Source Data Figs. 1 and 2 and Extended Data Figs. 1, 4 and 6 PDF file contains antibody information, protein ladder marker information, protein size information and uncropped film scans of western blots with the dashed rectangle indicating the cropped region.

Source Data Fig. 1 Source data Excel file with worksheet tabs for Fig. 1a,b,d.

Source Data Fig. 2 Source data Excel file with worksheet tabs for Fig. 2b,d,e.

Source Data Fig. 3 Source data Excel file with worksheet tabs for Fig. 3b,c,e.

Source Data Fig. 4 Source data Excel file with worksheet tabs for Fig. 4b–f.

Source Data Fig. 5 Source data Excel file with worksheet tabs for Fig. 5a–f.

Source Data Fig. 6 Source data Excel file with worksheet tabs for Fig. 6a–d.

Source Data Extended Data Fig. 1 Source data Excel file with worksheet tabs for Extended Data Fig. 1c,d.

Source Data Extended Data Fig. 2 Source data Excel file with worksheet tabs for Extended Data Fig. 2a–d.

Source Data Extended Data Fig. 3 Source data Excel file with worksheet tabs for Extended Data Fig. 3a,b,d–f.

Source Data Extended Data Fig. 4 Source data Excel file with worksheet tabs for Extended Data Fig. 4a,c,f,g.

Source Data Extended Data Fig. 5 Source data Excel file with worksheet tabs for Extended Data Fig. 5a–c.

Source Data Extended Data Fig. 6 Source data Excel file with worksheet tabs for Extended Data Fig. 6a–d.

Source Data Extended Data Fig. 7 Source data Excel file with worksheet tabs for Extended Data Fig. 7a,c–e.

Source Data Extended Data Fig. 8 Source data Excel file with worksheet tabs for Extended Data Fig. 8a–e.

## Data Availability

Access to the full Cancer DMC datasets requires DMC membership with the Broad Institute. Cancer cell line feature information is from public sources (https://cellmodelpassports.sanger.ac.uk, https://depmap.org/portal, https://tp53.isb-cgc.org/, https://www.cbioportal.org), the DMC and published reports^50–53^. Additional data supporting this study are available from the corresponding author upon reasonable request. Source data are provided with this paper.

## Code availability

The linear mixed-effect model is implemented as a custom application called IVEA and is Amgen’s proprietary code for statistical analysis and is not available for external use.
