# Supplementary material for: Small-molecule inhibition of kinesin KIF18A reveals a mitotic vulnerability enriched in chromosomally unstable cancers
Source: Nat Cancer. 2023 Dec 27;5(1):66–84. doi: 10.1038/s43018-023-00699-5 (PMC10824666; doi:10.1038/s43018-023-00699-5)
Supplement: Supplementary file 1 — Reporting Summary [file 43018_2023_699_MOESM1_ESM.pdf]

nature portfolio

Corresponding author(s): Marc Payton

Last updated by author(s): Nov 28, 2023

Reporting Summary

Nature Portfolio wishes to improve the reproducibility of the work that we publish. This form provides structure for consistency and transparency in reporting. For further information on Nature Portfolio policies, see our Editorial Policies and the Editorial Policy Checklist.

Statistics

For all statistical analyses, confirm that the following items are present in the figure legend, table legend, main text, or Methods section.

n/a	Confirmed	
☐	☒	The exact sample size (*n*) for each experimental group/condition, given as a discrete number and unit of measurement
☐	☒	A statement on whether measurements were taken from distinct samples or whether the same sample was measured repeatedly
☐	☒	The statistical test(s) used AND whether they are one- or two-sided<br>*Only common tests should be described solely by name; describe more complex techniques in the Methods section.*
☒	☐	A description of all covariates tested
☐	☒	A description of any assumptions or corrections, such as tests of normality and adjustment for multiple comparisons
☐	☒	A full description of the statistical parameters including central tendency (e.g. means) or other basic estimates (e.g. regression coefficient) AND variation (e.g. standard deviation) or associated estimates of uncertainty (e.g. confidence intervals)
☐	☒	For null hypothesis testing, the test statistic (e.g. F, t, r) with confidence intervals, effect sizes, degrees of freedom and P value noted<br>*Give P values as exact values whenever suitable.*
☒	☐	For Bayesian analysis, information on the choice of priors and Markov chain Monte Carlo settings
☒	☐	For hierarchical and complex designs, identification of the appropriate level for tests and full reporting of outcomes
☒	☐	Estimates of effect sizes (e.g. Cohen's d, Pearson's r), indicating how they were calculated

Our web collection on statistics for biologists contains articles on many of the points above.

Software and code

Policy information about availability of computer code

Data collection	Please see Methods section for data collection details. Data collected on EnVision Manager plate reader V1.13+ software, SpectraMax M2 plate reader software, ArrayScan VTi HCS reader with Studio software, iCys LSC with iGeneration V7 software, Nikon Elements V5 software, UltraView VoX with Volocity software, Opera Phenix HSC system software, Vi-CELL XR cell viability analyzer software, Incucyte software, HP digital scanner software, BD FACSDiva V8+ software, MicroBeta plate reader software, MSD Sector Imager S16000 plate reader software, StudyDirector v3.1 software, and Microsoft Excel for Office 365 software. PRISM screen data collection performed by Broad Institute. Kinome binding assay data collection performed by Eurofins DiscoverX. ELIPA data collection performed by Cytoskeleton Inc. A subset of in vivo efficacy data collection performed by Charles River Laboratories and Champions Oncology. Mouse blood count data collection/analysis performed by IDEXX Bioresearch.
Data analysis	Please see Methods section for data analysis details. Data analysis software included Microsoft Excel for Office 365 software, GraphPad Prism V7.05+ software, Genedata Screener software, ArrayScan VTi HCS with Studio software, iCys LSC with iGeneration V7 software, Nikon Elements V5 software, UltraView VoX with Volocity software, Columbus image analysis software, FSC-Express V6+ software, Incucyte software, and Image Lab 6.1 software. Access to the full Cancer Dependency Map Consortium (DMC) analysis tools require a DMC membership with the Broad Institute. PRISM screen primary data analysis performed by Broad Institute. Kinome binding assay data analysis performed by Eurofins DiscoverX. ELIPA data analysis performed by Cytoskeleton Inc. A subset of in vivo efficacy data analysis performed by Charles River Laboratories and Champions Oncology. Statistical data analysis for tumor efficacy studies performed using Linear Mixed Effects Models with IVEA application using R-CRAN software package at Amgen. The custom IVEA application is Amgen's proprietary code for statistical analysis and is not available for external use.

For manuscripts utilizing custom algorithms or software that are central to the research but not yet described in published literature, software must be made available to editors and reviewers. We strongly encourage code deposition in a community repository (e.g. GitHub). See the Nature Portfolio guidelines for submitting code & software for further information.

Data

Policy information about availability of data

All manuscripts must include a data availability statement. This statement should provide the following information, where applicable:

- Accession codes, unique identifiers, or web links for publicly available datasets
- A description of any restrictions on data availability
- For clinical datasets or third party data, please ensure that the statement adheres to our policy

Source data provided for this study. The PRISM screen cancer cell line ID and AM-1882 AUC values provided in Supplementary Tables and Source data. Access to the full Cancer Dependency Map Consortium (DMC) datasets require a DMC membership with the Broad Institute. Cancer cell line feature information obtained from public database resources (https://depmap.org/portal, https://cellmodelpassports.sanger.ac.uk, https://tp53.isb-cgc.org/, https://www.cbioportal.org), DMC, and published reports (references 50-53). TNBC PDX tumor model information provided as a Supplementary Table. All other data information supporting this study are available from the corresponding author upon a reasonable request.

Human research participants

Policy information about studies involving human research participants and Sex and Gender in Research.

Reporting on sex and gender	*Use the terms sex (biological attribute) and gender (shaped by social and cultural circumstances) carefully in order to avoid confusing both terms. Indicate if findings apply to only one sex or gender; describe whether sex and gender were considered in study design whether sex and/or gender was determined based on self-reporting or assigned and methods used. Provide in the source data disaggregated sex and gender data where this information has been collected, and consent has been obtained for sharing of individual-level data; provide overall numbers in this Reporting Summary. Please state if this information has not been collected. Report sex- and gender-based analyses where performed, justify reasons for lack of sex- and gender-based analysis.*
Population characteristics	*Describe the covariate-relevant population characteristics of the human research participants (e.g. age, genotypic information, past and current diagnosis and treatment categories). If you filled out the behavioural & social sciences study design questions and have nothing to add here, write "See above."*
Recruitment	*Describe how participants were recruited. Outline any potential self-selection bias or other biases that may be present and how these are likely to impact results.*
Ethics oversight	*Identify the organization(s) that approved the study protocol.*

Note that full information on the approval of the study protocol must also be provided in the manuscript.

Field-specific reporting

Please select the one below that is the best fit for your research. If you are not sure, read the appropriate sections before making your selection.

☒ Life sciences ☐ Behavioural & social sciences ☐ Ecological, evolutionary & environmental sciences

For a reference copy of the document with all sections, see nature.com/documents/nr-reporting-summary-flat.pdf

Life sciences study design

All studies must disclose on these points even when the disclosure is negative.

Sample size	Please see Methods section for experimental sample size details. For in vitro and in vivo studies, no statistical tests were used to predetermine sample sizes. The experimental sample size varied across siRNA and small molecule inhibitor studies. PRISM screen sample size was based on the cancer cell lines with reported AM-1882 AUC values. For in vivo tumor efficacy and PD/PK studies, mice with established tumors were randomized into treatment groups (n = 10 mice per group) and (n = 3 mice per group), respectively.
Data exclusions	In the Methods section, we state the PRISM screen Kuramochi cell data was excluded from our analysis. We cropped the Western immunoblot film scans and removed some of the lanes not pertinent to this study. All the uncropped immunoblot film scans are provided in a single PDF source file. On occasion, we excluded data points during curve-fitting QC, excluded data points are noted in Source data files.
Replication	Please see Methods section and Figure legends for replication details. Source data provided for this study. We provide the number of replicates and independent experiments performed for each in vitro enzyme and cellular assay. A subset of in vitro experiments were reported once (e.g. Western blot analysis). All in vivo studies were performed once.
Randomization	Sample randomization is not relevant to the in vitro studies presented. For in vivo efficacy studies, mice with established tumors were randomized into treatment groups (n = 10 mice per group) based on similar mean and SEM tumor volumes at the start of the study.
Blinding	Blinding was not performed for in vitro and in vivo studies conducted by Amgen. Studies conducted and analyzed by contract research

Blinding

organizations (Cytoskeleton Inc, Eurofins DiscoverX, Charles Rivers Laboratories, Champions Oncology, IDEXX Bioresearch) and by the Broad Institute were provided only a compound ID, molecular weight, test concentration (s), formulation, stability, and storage information.

Reporting for specific materials, systems and methods

We require information from authors about some types of materials, experimental systems and methods used in many studies. Here, indicate whether each material, system or method listed is relevant to your study. If you are not sure if a list item applies to your research, read the appropriate section before selecting a response.

Materials & experimental systems

n/a	Involved in the study
☐	☒ Antibodies
☐	☒ Eukaryotic cell lines
☒	☐ Palaeontology and archaeology
☐	☒ Animals and other organisms
☒	☐ Clinical data
☒	☐ Dual use research of concern

Methods

n/a	Involved in the study
☒	☐ ChIP-seq
☐	☒ Flow cytometry
☒	☐ MRI-based neuroimaging

Antibodies

Antibodies used

Antibodies (Western blotting).
1. Anti cleaved-PARP (cl-PARP) (552597, BD Pharmingen, mouse, 1: 500)
2. Anti-cyclin B1 (554179, BD Pharmingen, mouse, 1: 500)
3. Anti-β-actin (A5441, Sigma, mouse, 1: 5000)
4. Anti-GAPDH (2118, Cell Signaling, rabbit, 1: 10,000)
5. Anti-phospho-γH2AX (serine-139) (γH2AX, 05-636, Millipore, mouse, 1: 2000)
6. Anti-MCL-1 (5453, Cell Signaling, rabbit, 1: 500)
7. Anti-cyclin E1 (MS-870-P, HE12, NeoMarkers, mouse, 1: 2000)
8. Anti-BubR1 (612503, BD Pharmingen, mouse, 1: 5000)
9. Anti-KIF18A (HPA039484, Sigma, rabbit, 1: 2000)
10. Anti-Eg5 (ab51976, Abcam, mouse, 1: 2000)
11. Anti-phospho-Histone H3 (serine-10) (pH3, 06-570, Millipore, rabbit, 1: 2000)
12. Anti-p21 (2947, Cell Signaling, rabbit, 1: 1000)
13. Anti-total-RB (554136, BD Pharmingen, mouse, 1: 300)
14. Anti-phospho-RB (serine-807/811) (9308, Cell Signaling, rabbit, 1: 1000)
15. Anti-securin (ab79546, Abcam, rabbit, 1: 500)
16. Anti-p16 (554079, BD Pharmingen, mouse, 1: 500)
17. Anti-BRCA1 (N-term, 9010S, Cell Signaling, rabbit, 1: 1000)
18. Anti-BRCA1 (C-term, 07-434, Millipore, rabbit, 1: 2000).

Antibodies (immunofluorescence, imaging).
1. Anti-phospho-Histone H3 (serine-10) (pH3, 05-806, Millipore, mouse, 1: 2000)
2. Anti-phospho-Histone H3 (serine-10) (pH3, 06-570, Millipore, rabbit, 5 μg per mL)
3. Anti-pericentrin (Ab4448, Abcam, rabbit, 1: 2000)
4. Anti-BrdU-AlexaFluor-647 (B35140, Invitrogen, mouse, 3 μg per mL)
5. Anti-p21 (12D1) (2947, Cell Signaling, rabbit, 1: 400)
6. Anti-cleaved-PARP (214/215) (44-6986, Invitrogen, rabbit, 1: 1500)
7. Anti-phospho-γH2AX (serine-139) (05-636, Millipore, mouse, 1: 1000)
8. Anti-KIF18A (A301-080A, Bethyl, rabbit, 1: 3000)
9. Anti-centrin-3 (H00001070, Abnova, mouse, 1: 2000)
10. Anti-α-tubulin (T6199, Sigma, mouse, 1: 3000 or 5 μg per mL)
11. Anti-cGAS (15102, Cell Signaling, rabbit, 1: 500)
12. Anti-tubulin β3 (clone TUJ1, 801201, Biolegend, mouse, 1: 500)
13. Anti-mouse-IgG-AlexaFluor-568 (A11004, Invitrogen, 1: 2000)
14. Anti-rabbit-IgG- AlexaFluor-488 (A11034, Invitrogen, 1: 1000 to 2000)
15. Anti-mouse-IgG-AlexaFluor-647 (A21235, Invitrogen, 1: 1000 to 2000)
16. Anti-rabbit-IgG-AlexaFluor-647 (A21245, Invitrogen, 1: 1000 to 2000)
17. Anti-mouse-IgG-AlexaFluor-488 (A11029, Invitrogen, 1: 1000 to 2000)
18. Anti-rabbit-IgG- AlexaFluor-568 (A11036, Invitrogen, 1: 2000)
19. Anti-mouse-IgG-AlexaFluor-488 (115-545-206, Jackson ImmunoResearch, 1: 500)

Antibodies (immunofluorescence, flow cytometry).
1. Anti-BrdU-AlexaFluor-647 (B35140, Invitrogen, mouse, 3 μg per mL).
2. Anti-human CD243 (P-gp)-APC (348608, BioLegend, mouse, 40 μg/mL).
3. Isotype-APC control (400220, BioLegend, mouse, 40 μg/mL).

Validation

All antibodies were validated by the manufacturer and citation information noted on manufactures websites. Please refer to the manufacturers' websites with the catalog information listed above. Additional antibody validation by Amgen was performed for Western and IF applications. Methods used to validate antibodies included antibody titration, siRNA directed KD of target protein, +/-

treatment with agents that modulate target protein expression, profiling target protein expression in amplified or deleted cell lines, and cell organelle protein localization.

Western (W), IFI (IF imaging), IFF (IF flow cytometry).

Anti cleaved-PARP (cl-PARP), validation by +/- treatment with chemotherapeutic agents (W, IFI)
Anti-phospho-γH2AX (serine-139), validation by +/- treatment with chemotherapeutic agents (W, IFI)
Anti-phospho-Histone H3 (serine-10), validation by +/- treatment with nocodazole (W, IFI)
Anti-cyclin B1, validation by +/- treatment with nocodazole (W)
Anti-cyclin E1, validation by profiling CCNE1 wild-type and CCNE1 amplified cell lines (W)
Anti-BubR1, validation by +/- treatment with nocodazole (W)
Anti-securin, validation by +/- treatment with nocodazole (W)
Anti-KIF18A, validation by +/- treatment with nocodazole (W), gene knockdown with siRNAs (W, IFI)
Anti-Eg5, validation by +/- treatment with nocodazole (W), gene knockdown with siRNAs (W, IFI)
Anti-cGAS, validation by gene knockdown with siRNAs (IFI)
Anti-MCL-1, validation by +/- treatment with chemotherapeutic agents and MCL-1 inhibitor (W)
Anti-p21, validation by +/- treatment with MDM2 inhibitor in TP53 wild-type cell line (W, IFI)
Anti-total-RB, validation by profiling RB1 wild-type and RB1 deleted cell lines (W)
Anti-phospho-RB, validation by +/- treatment with CDK4/6 inhibitor in RB1 wild-type cell line (W)
Anti-BRCA1 (N-term and C-term), validation by gene knockdown with siRNAs (W)
Anti-BrdU, validation by +/- treatment with BrdU and chemotherapeutic agents (IFI, IFF)
Anti-centrin-3, anti-pericentrin, anti-α-tubulin, and anti-β3-tubulin, validation by cell organelle protein localization (IFI)

Eukaryotic cell lines

Policy information about cell lines and Sex and Gender in Research

Cell line source(s)

Cancer cell lines were sourced from ATCC (American Type Culture Collection) and DSMZ (Deutsche Sammlung von Mikroorganismen und Zellkulturen), unless otherwise specified. Cell lines submitted to ATCC for authentication by short tandem repeat (STR) DNA analysis. Seventeen STR loci plus the gender determining locus, Amelogenin, were amplified using the commercially available PowerPlex® 18D Kit from Promega. The cell line sample was processed using the ABI Prism® 3500xl Genetic Analyzer. Data were analyzed using GeneMapper® ID-X v1.2 software (Applied Biosystems).

HCC-1806 (sourced from ATCC, CRL-2335, last STR testing April-2019)
BT-549 (sourced from ATCC, HTB-122, last STR testing April-2019)
MDA-MB-157 (sourced from ATCC, HTB-24, last STR testing June-2018)
OVCAR-3 (sourced from ATCC, HTB-161, aka NIHOVCAR-3 by Amgen, last STR testing July-2019)
MCF-7 (sourced from ATCC, HTB-22, last STR testing April-2019)
MDA-MB-453 (sourced from ATCC, HTB-131, last STR testing July-2019)
ZR-75-1 (sourced from ATCC, CRL-1500, last STR testing June-2018)
HCC-1937 (sourced from ATCC, CRL-2336, last STR testing January-2019)
HeLa (sourced from ATCC, CCL-2, last STR testing October-2018)
CAL-51 (sourced from DSMZ, ACC 302, last STR testing March-2017)
JIMT-1 (sourced from DSMZ, ACC 589, STR testing performed prior to cell line procurement)
OVCAR-5 (originally sourced from the National Cancer Institute, last STR testing July-2019)
OVCAR-8 (originally sourced from the National Cancer Institute, last STR testing August-2019)
OVCAR-8 ADRRES (originally sourced from the National Cancer Institute, aka NCI-ADR-RES, last STR testing July-2018)
MDA-MB-157 Cas9 (sourced from Cellecta, custom MDA-MB-157 cells expressing Cas9, no STR information provided)
HeLa Kyoto α-tubulin-EGFP/H2B-mCherry (sourced from Creative Bioarray Inc., CSC-C8812H, no STR information provided)
DNA-barcoded cancer cell line collection established by the Broad Institute (see www.theprismlab.org).

Primary Human Normal Cell Line Source Information

Human Bone Marrow Mononuclear Cells (Lonza Inc., 2S-101D, four donors (#37612, #37534, #36223, #45133))
Human Mammary Epithelial Cells (Lonza Inc., CC-2551, one donor, 2 vials)
Human Foreskin Fibroblast Cells (ATCC, CRL-2091, one donor)
Human CD3+ T-Cells (HemaCare/CRL Inc., two donors (#107136, #155092))
Human Induced Pluripotent Stem Cells, hiPSC (Thermo Fisher Inc., A18945)

Source Information for culture medium and components to derive hiPSC sensory neurospheres.

Compound (source): CHIR99021 (Tocris), DAPT (Tocris), DMH1 (Tocris), SB431542 (Tocris), SU-5402 (abcam), Y-27632 (Tocris).
Company Abbreviations: ThermoFisher (TF), Stem Cell Technologies (SCT).

- E8F-defined medium (Essential 8-Flex, P/S, 10 µM Y-27632)
- SBCHIR-defined medium (Essential 6, P/S, 10 µM SB431542, 0.6 µM CHIR99021)
- DMHB-defined medium (Essential 6, P/S, 10 µM SB431542, 0.6 µM CHIR99021, 1µM DMH1, 15 ng/mL BMP4 (TF)).
- NC-defined medium (DMEMF/12, N-2, B-27, NEAA, GlutaMax, P/S, Laminin, 0.6 µM CHIR99021, 10 ng/mL FGF2 (TF))
- SNP-defined medium (DMEMF/12, N-2, B-27, NEAA, GlutaMax, P/S, Laminin, 0.6 µM CHIR99021, 5 µM SU-5402, and 10 µM DAPT)
- SNI-defined medium (Brainphys (SCT), N-2, B-27, P/S, laminin, growth factors (25 ng/mL NGF, 10 ng/mL BDNF, 25 ng/mL GDNF, 10 ng/mL NT-3 (Peprotech)), CultureOne (TF), retinoic acid (Tocris), 2 µM DAPT)
- SNM-defined medium (Brainphys, N-2, B-27, P/S, NGF, BDNF, GDNF, NT-3, CultureOne, retinoic acid)
- NO-defined medium (Brainphys, N-2, B-27, P/S, NGF, BDNF, GDNF, NT-3, retinoic acid, and laminin)

Authentication	Please see cell line source section above for authentication information for each cancer cell line. DNA-barcoded cancer cell line collection was authenticated by short tandem repeat (STR) DNA analysis performed by the Broad Institute (see www.theprismlab.org).
Mycoplasma contamination	All cancer cell lines used for in vivo studies were confirmed to be negative for mycoplasma contamination and a panel of murine vial pathogens. Cell lines used for in vitro studies were mycoplasma negative upon purchase. Repeated mycoplasma testing was not performed on cell lines after initial thaw for in vitro expansion. The PRISM DNA-barcoded cancer cell line collection tested mycoplasma negative.
Commonly misidentified lines (See ICLAC register)	In vitro experiments were performed in OVCAR-8 variant MDR "NCI-ADR-RES" (also known as ADRRES) cell line derived from parental cells by selection with adriamycin (aka doxorubicin). NCI-ADR-RES cells STR DNA testing score was 97.3% match with OVCAR-8 parental cells. Please see Cellosaurus (https://www.cellosaurus.org/CVCL_1452).

Animals and other research organisms

Policy information about studies involving animals; ARRIVE guidelines recommended for reporting animal research, and Sex and Gender in Research

Laboratory animals	Female mice (athymic nude, CB.17 SCID). Mice 6- to 8-weeks old at tumor implantation.
Wild animals	Our studies did not use wild animals
Reporting on sex	In vivo tumor efficacy and PD studies conducted in female mice.
Field-collected samples	This study did not involve field-collection
Ethics oversight	All animal experimental protocols were approved by the Amgen Animal Care and Use Committee (AACUC) and were conducted in accordance with the guidelines set by the Association for Assessment and Accreditation of Laboratory Animal Care. Mice were housed in an environmentally controlled room (temperature 23 ± 2°C, relative humidity 50 ± 20%) on a 12-hour light/dark cycle. Mice were offered commercial rodent chow and water ad libitum. Mice with a measurable tumor size exceeding 2000 mm^3 were removed from the study and euthanized. Source files provided for individual animal tumor size and body weights.

Note that full information on the approval of the study protocol must also be provided in the manuscript.

Flow Cytometry

Plots

Confirm that:

☒ The axis labels state the marker and fluorochrome used (e.g. CD4-FITC).

☒ The axis scales are clearly visible. Include numbers along axes only for bottom left plot of group (a 'group' is an analysis of identical markers).

☒ All plots are contour plots with outliers or pseudocolor plots.

☒ A numerical value for number of cells or percentage (with statistics) is provided.

Methodology

Sample preparation	Sample preparation described in the Methods section for BrdU, cell cycle, and P-gp cell surface analysis by flow cytometry.
Instrument	BD LSRFortessa
Software	Analysis with BD FACSDiva software, and post-acquisition data analysis was performed using FSC-Express software.
Cell population abundance	Cell abundance was sufficient for flow cytometry analysis.
Gating strategy	BrdU analysis - double discrimination primary gate based on DNA content with DAPI stain. Gate on BrdU+ cells.<br>P-gp cell surface analysis - viability_size primary gate to exclude dead cells with PO-PRO-1 Iodide DNA dye. Gate on P-gp + cells. We provide representative flow cytometry plots for the gating schemes in Source files.

☒ Tick this box to confirm that a figure exemplifying the gating strategy is provided in the Supplementary Information.
