# Supplementary material for: Small-molecule inhibition of kinesin KIF18A reveals a mitotic vulnerability enriched in chromosomally unstable cancers
Source: Nat Cancer. 2023 Dec 27;5(1):66–84. doi: 10.1038/s43018-023-00699-5 (PMC10824666; doi:10.1038/s43018-023-00699-5)
Supplement: Supplementary file 5 — PDF file contains antibody information, protein ladder marker information, protein size information and uncropped film scans of western blots with the dashed rectangle indicating the cropped region. [file 43018_2023_699_MOESM5_ESM.pdf]

Western blot analysis: Antibody Information

Antibody Information	Protein band (s) size and information
anti-KIF18A (HPA039484, Sigma, rabbit, 1: 2000)	Single or doublet (Nocodazole treatment) bands at ~110 kDa
anti-Eg5 (ab51976, Abcam, mouse, 1: 2000)	Single band at ~120 kDa
anti-cleaved-PARP (cl-PARP) (552597, BD Pharmingen, mouse, 1: 500)	Single band at ~89 kDa
anti-MCL-1 (5453, Cell Signaling, rabbit, 1: 500)	Single band at ~37 kDa
anti-cyclin B1 (554179, BD Pharmingen, mouse, 1: 500)	Single band at ~62 kDa
anti-β-actin (A5441, Sigma, mouse, 1: 5000)	Single band at ~42 kDa
anti-securin (ab79546, Abcam, rabbit, 1: 500)	Single band at ~25 kDa
anti-cyclin E1 (MS-870-P, HE12, NeoMarkers, mouse, 1: 2000)	Full length band at ~50 kDa, Lower MW bands ~45 to ~35 kDa
anti-total-RB (554136, BD Pharmingen, mouse, 1: 300)	Broad band at ~115 kDa, total Rb has multiple bands due to its phosphorylation status
anti-phospho-RB (serine-807/811) (9308, Cell Signaling, rabbit, 1: 1000)	Doublet band at ~115 kDa
anti-p16 (554079, BD Pharmingen, mouse, 1: 500)	Single band at ~16 kDa
anti-p21 (2947, Cell Signaling, rabbit, 1: 1000)	Single band at ~21 kDa
anti-GAPDH (2118, Cell Signaling, rabbit, 1: 10,000)	Single band at ~37 kDa
anti-BubR1 (612503, BD Pharmingen, mouse, 1: 5000)	Single or doublet (Nocodazole treatment) ~ 125 kDa
anti-γHistone H2AX (γH2AX, 05-636, Millipore, mouse, 1: 2000)	Single band at ~17 kDa
anti-p-Histone H3 (serine-10) (pH3, 06-570, Millipore, rabbit, 1: 2000)	Single band at ~17 kDa
anti-BRCA1 (C-term, 07-434, Millipore, rabbit, 1: 2000)	Multiple bands at ~ 220 kDa, used BRCA1 siRNA KD to determined specific protein band
anti-BRCA1 (C-term, 07-434, Millipore, rabbit, 1: 2000)	Multiple bands at ~ 220 kDa, used BRCA1 siRNA KD to determined specific protein band

Western blot analysis: Immunoblot molecular weight marker information

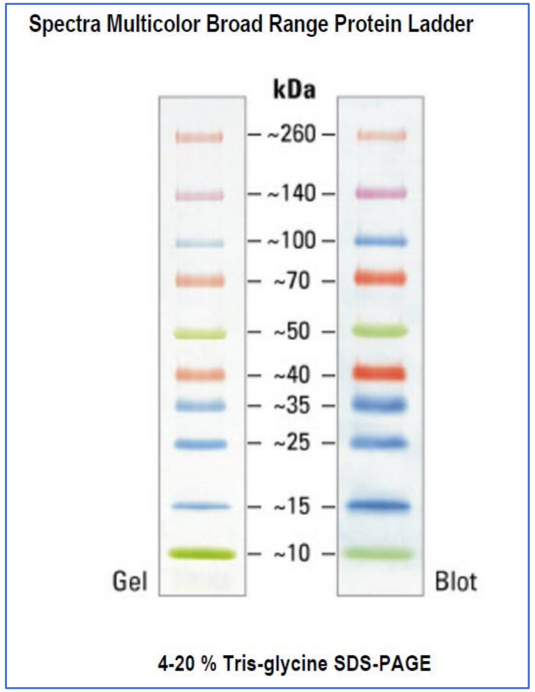

Please note Spectra Multicolor Broad Range Protein Ladder was included on every Western immunoblot. Multiple film exposures were captured on film for each immunoblot. A subset of the film scans are missing the Sharpie pen ladder marks, an arrow indicates the appropriate size protein band of interest.

Figure 1e

Western Analysis

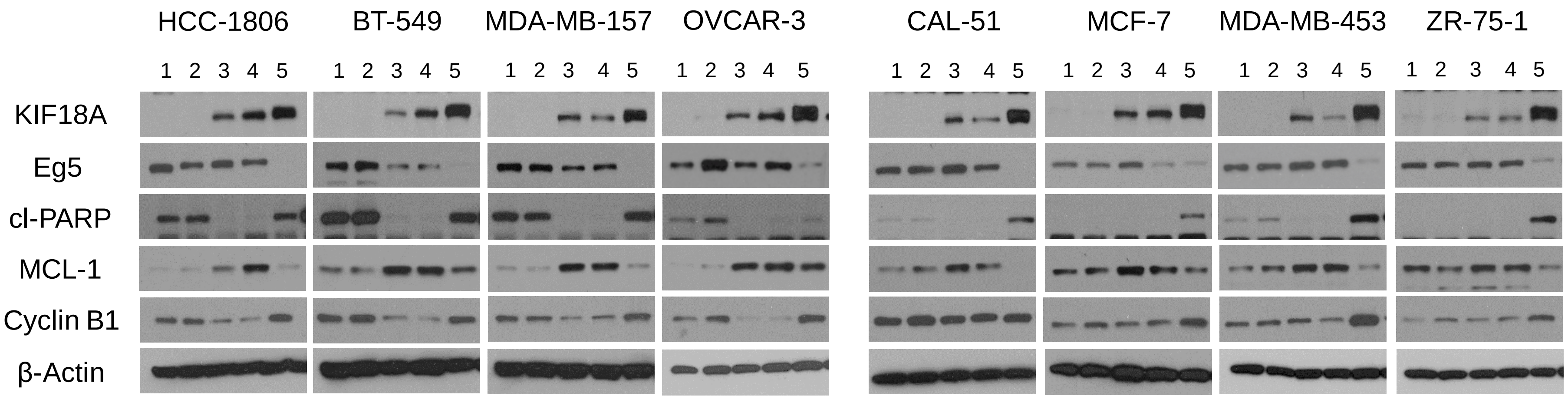

ID	siRNA
1	KIF18A^{2}
2	KIF18A^{4}
3	NTC^{1}
4	NTC^{2}
5	Eg5^{1}

Adjusted brightness equally

HCC-1806 Immunoblots, uncropped scans of film

Figure 1e

Anti-KIF18A

Rabbit anti-KIF18A antibody
(1:3000; Sigma, Cat# HPA 039484)

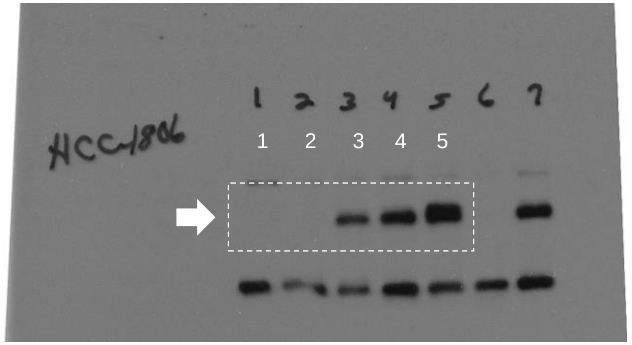

KIF18A band at ~110 kDa

Anti-Eg5

Mouse anti-Eg5 antibody
(1:2000; Abcam, Cat# ab51976)

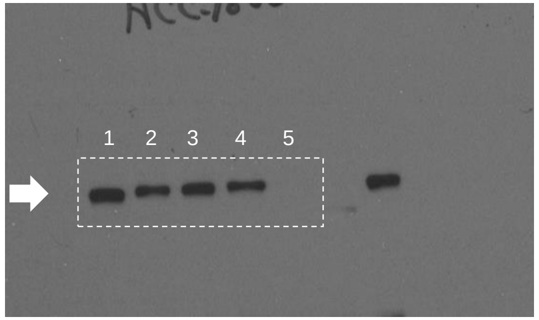

Eg5 band at ~120 kDa

Anti-cleaved PARP

Mouse anti-cl-PARP antibody
(1:500; BD Pharmingen, Cat# 51-900017)

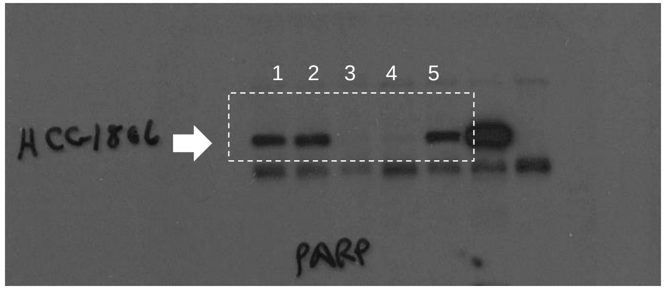

Cl-PARP band at ~89 kDa

Anti-MCL-1

Rabbit anti-MCL-1 antibody
(1:500; Cell Signaling, Cat# 5453)

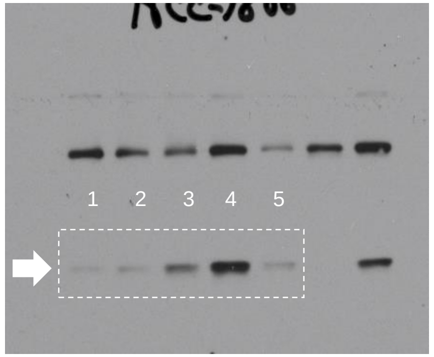

MCL-1 band at ~37 kDa

Anti-Cyclin B1

Mouse anti-Cyclin B1 antibody
(1:500; BD Pharmingen, Cat# 554179)

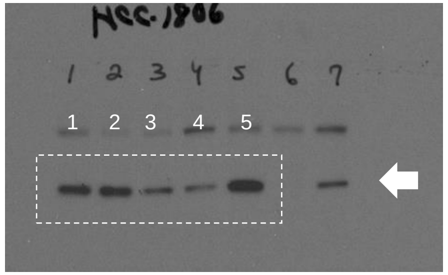

Cyclin B1 band at ~62 kDa

Anti-β-Actin

Mouse anti-β-Actin antibody
(1:5000; Sigma, Cat# A5441)

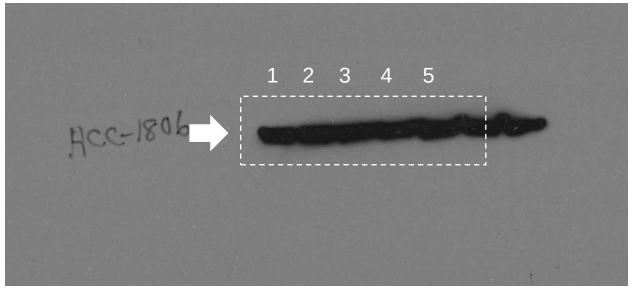

β-Actin band at ~42 kDa

Lane ID	siRNA
1	KIF18A^{2}
2	KIF18A^{4}
3	NTC^{1}
4	NTC^{2}
5	Eg5^{1}
6	1 µM Staurosporine Control
7	Untreated Control

Lanes 6 & 7 cropped out

- Dashed rectangle box = cropped region
- Adjusted brightness equally (+20)

BT-549 Immunoblots, uncropped scans of film

Figure 1e

Anti-KIF18A

Rabbit anti-KIF18A antibody
(1:3000; Sigma, Cat# HPA 039484)

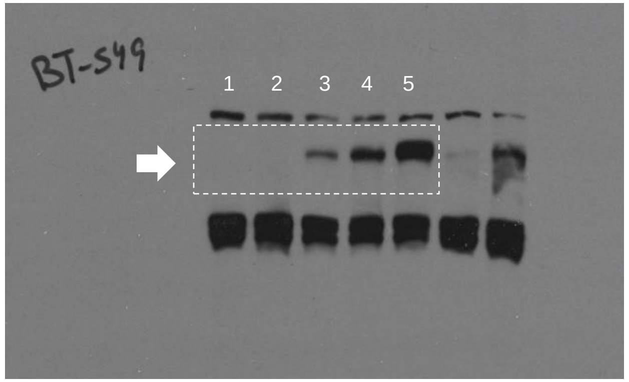

KIF18A band at ~110 kDa

Anti-Eg5

Mouse anti-Eg5 antibody
(1:2000; Abcam, Cat# ab51976)

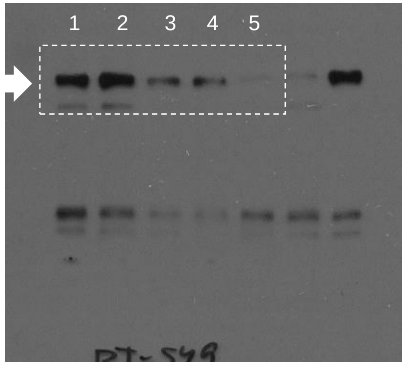

Eg5 band at ~120 kDa

Anti-cleaved PARP

Mouse anti-cl-PARP antibody
(1:500; BD Pharmingen, Cat# 552597)

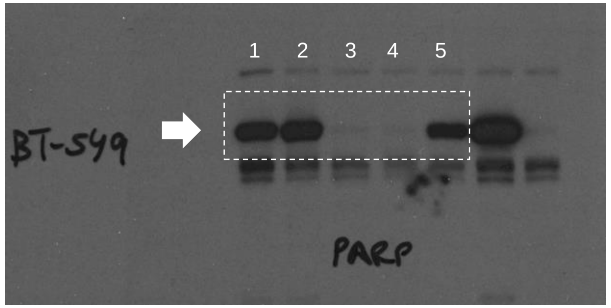

Cl-PARP band at ~89 kDa

Anti-MCL-1

Rabbit anti-MCL-1 antibody
(1:500; Cell Signaling, Cat# 5453)

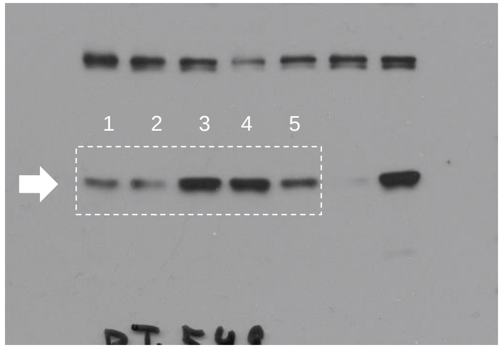

MCL-1 band at ~37 kDa

Anti-Cyclin B1

Mouse anti-Cyclin B1 antibody
(1:500; BD Pharmingen, Cat# 554179)

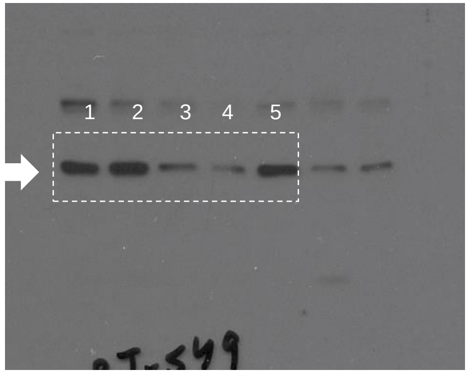

Cyclin B1 band at ~62 kDa

Anti-β-Actin

Mouse anti-β-Actin antibody
(1:5000; Sigma, Cat# A5441)

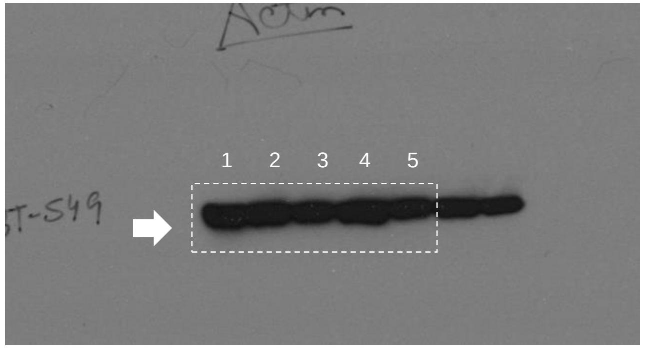

β-Actin band at ~42 kDa

Lane ID	siRNA
1	KIF18A[2]
2	KIF18A[4]
3	NTC[1]
4	NTC[2]
5	Eg5[1]
6	1 µM Staurosporine Control
7	Untreated Control

Lanes 6 & 7 cropped out

- Dashed rectangle box = cropped region
- Adjusted brightness equally (+20)

MDA-MB-157 Immunoblots, uncropped scans of film

Figure 1e

Anti-KIF18A

Rabbit anti-KIF18A antibody
(1:3000; Sigma, Cat# HPA 039484)

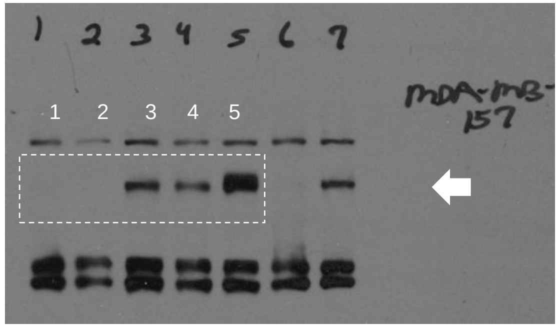

KIF18A band at ~110 kDa

Anti-Eg5

Mouse anti-Eg5 antibody
(1:2000; Abcam, Cat# ab51976)

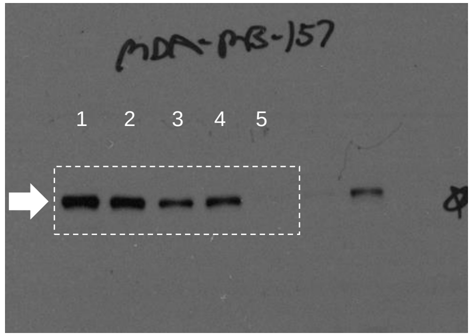

Eg5 band at ~120 kDa

Anti-cleaved PARP

Mouse anti-cl-PARP antibody
(1:500; BD Pharmingen, Cat# 552597)

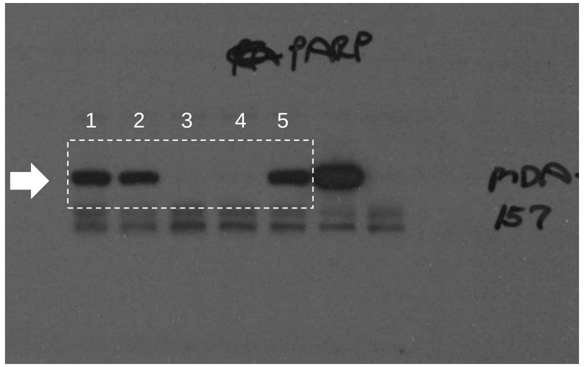

Cl-PARP band at ~89 kDa

Anti-MCL-1

Rabbit anti-MCL-1 antibody
(1:500; Cell Signaling, Cat# 5453)

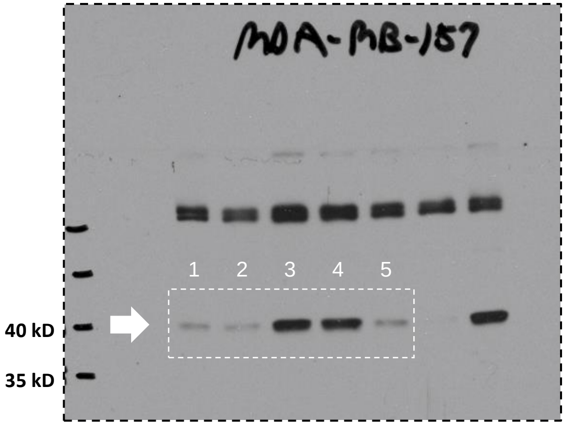

MCL-1 band at ~37 kDa

Anti-Cyclin B1

Mouse anti-Cyclin B1 antibody
(1:500; BD Pharmingen, Cat# 554179)

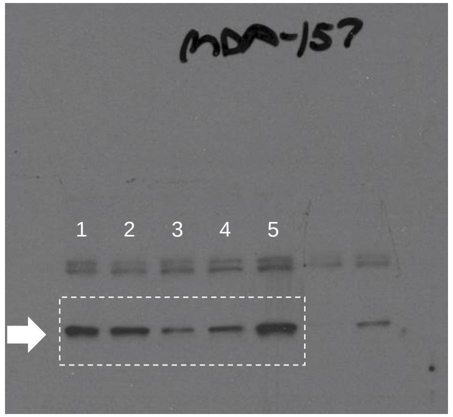

Cyclin B1 band at ~62 kDa

Anti-β-Actin

Mouse anti-β-Actin antibody
(1:5000; Sigma, Cat# A5441)

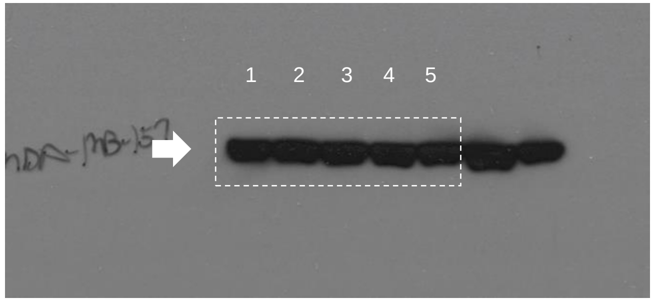

β-Actin band at ~42 kDa

Lane ID	siRNA
1	KIF18A[2]
2	KIF18A[4]
3	NTC[1]
4	NTC[2]
5	Eg5[1]
6	1 µM Staurosporine Control
7	Untreated Control

Land 6 & 7 cropped out

- Dashed rectangle box = cropped region
- Adjusted brightness equally (+20)

OVCAR-3 Immunoblots, uncropped scans of film

Figure 1e

Anti-KIF18A

Rabbit anti-KIF18A antibody
(1:3000; Sigma, Cat# HPA 039484)

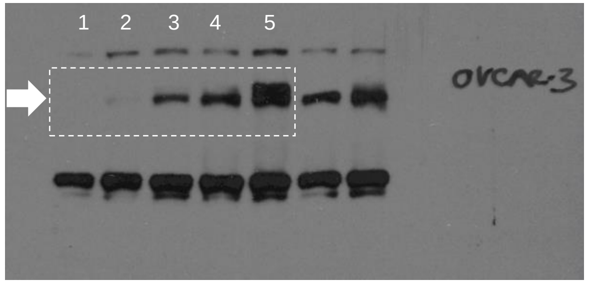

KIF18A band at ~110 kDa

Anti-Eg5

Mouse anti-Eg5 antibody
(1:2000; Abcam, Cat# ab51976)

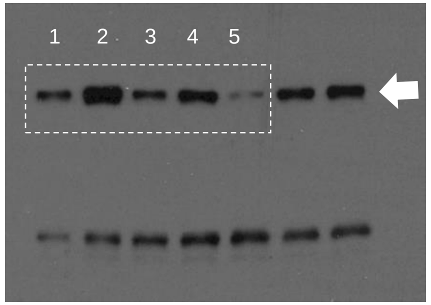

Eg5 band at ~120 kDa

Anti-cleaved PARP

Mouse anti-cl-PARP antibody
(1:500; BD Pharmingen, Cat# 552597)

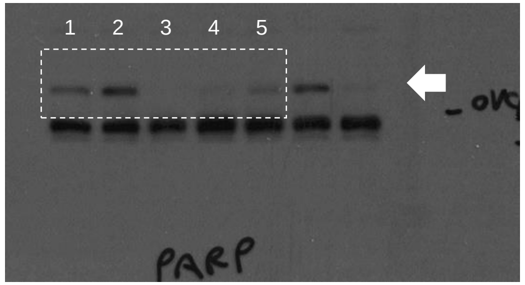

Cl-PARP band at ~89 kDa

Anti-MCL-1

Rabbit anti-MCL-1 antibody
(1:500; Cell Signaling, Cat# 5453)

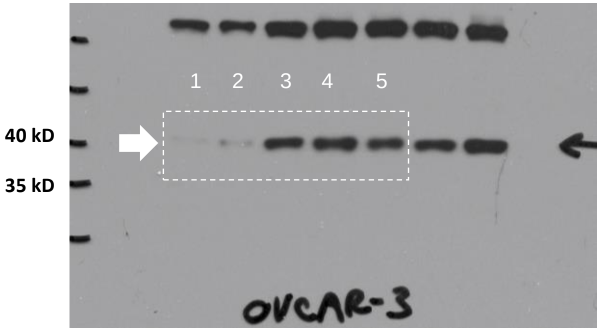

MCL-1 band at ~37 kDa

Anti-Cyclin B1

Mouse anti-Cyclin B1 antibody
(1:500; BD Pharmingen, Cat# 554179)

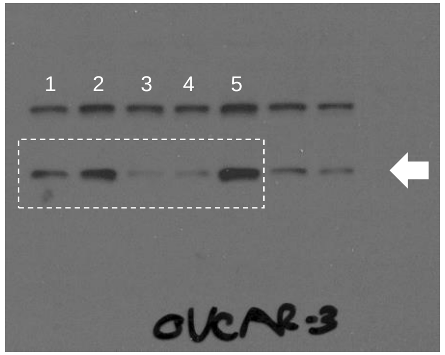

Cyclin B1 band at ~62 kDa

Anti-β-Actin

Mouse anti-β-Actin antibody
(1:5000; Sigma, Cat# A5441)

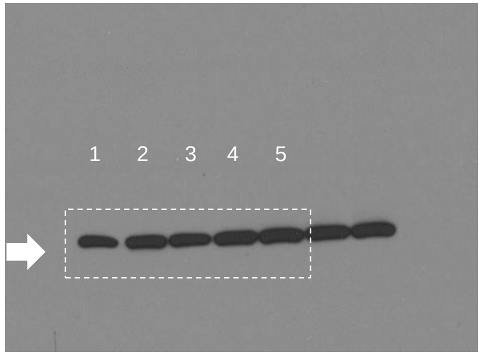

β-Actin band at ~42 kDa

Lane ID	siRNA
1	KIF18A[2]
2	KIF18A[4]
3	NTC[1]
4	NTC[2]
5	Eg5[1]
6	1 µM Staurosporine Control
7	Untreated Control

Lanes 6 & 7 cropped out

- Dashed rectangle box = cropped region
- Adjusted brightness equally (+20)

CAL-51 Immunoblots, uncropped scans of film

Figure 1e

Anti-KIF18A

Rabbit anti-KIF18A antibody
(1:3000; Sigma, Cat# HPA 039484)

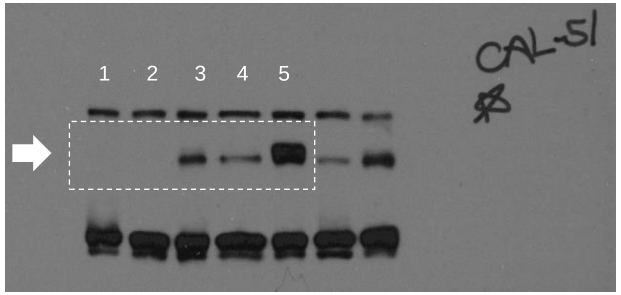

KIF18A band at ~110 kDa

Anti-Eg5

Mouse anti-Eg5 antibody
(1:2000; Abcam, Cat# ab51976)

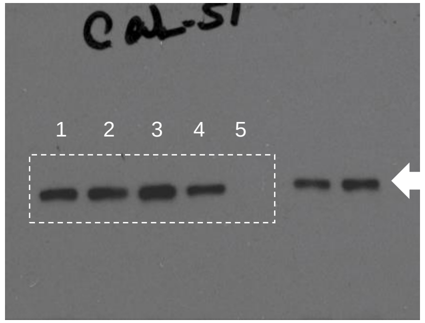

Eg5 band at ~120 kDa

Anti-cleaved PARP

Mouse anti-cl-PARP antibody
(1:500; BD Pharmingen, Cat# 552597)

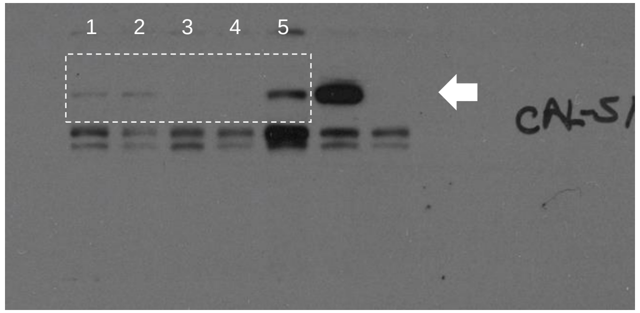

Cl-PARP band at ~89 kDa

Anti-MCL-1

Rabbit anti-MCL-1 antibody
(1:500; Cell Signaling, Cat# 5453)

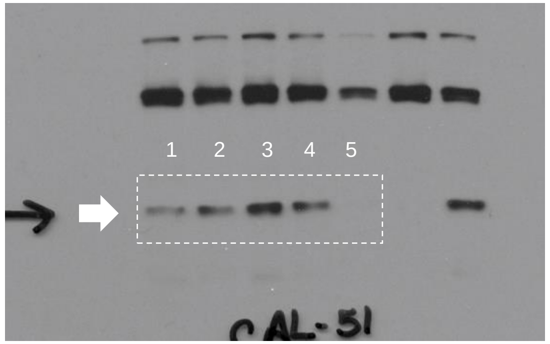

MCL-1 band at ~37 kDa

Anti-Cyclin B1

Mouse anti-Cyclin B1 antibody
(1:500; BD Pharmingen, Cat# 554179)

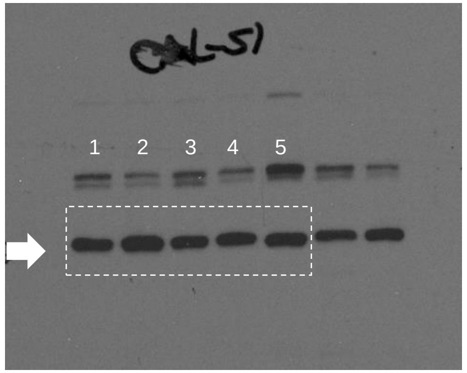

Cyclin B1 band at ~62 kDa

Anti-β-Actin

Mouse anti-β-Actin antibody
(1:5000; Sigma, Cat# A5441)

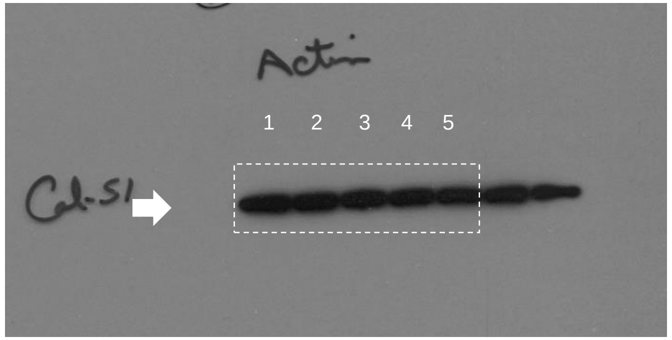

β-Actin band at ~42 kDa

Lane ID	siRNA
1	KIF18A[2]
2	KIF18A[4]
3	NTC[1]
4	NTC[2]
5	Eg5[1]
6	1 µM Staurosporine Control
7	Untreated Control

Lanes 6 & 7 cropped out

- Dashed rectangle box = cropped region
- Adjusted brightness equally (+20)

MCF-7 Immunoblots, uncropped scans of film

Figure 1e

Anti-KIF18A

Rabbit anti-KIF18A antibody
(1:3000; Sigma, Cat# HPA 039484)

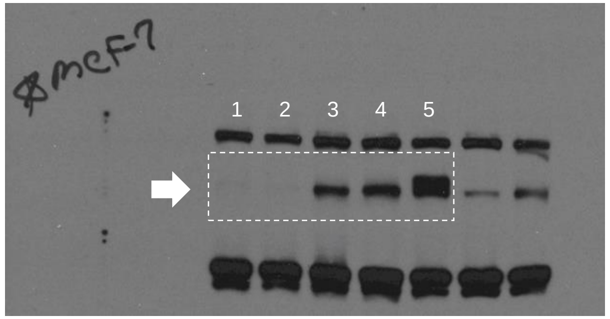

KIF18A band at ~110 kDa

Anti-Eg5

Mouse anti-Eg5 antibody
(1:2000; Abcam, Cat# ab51976)

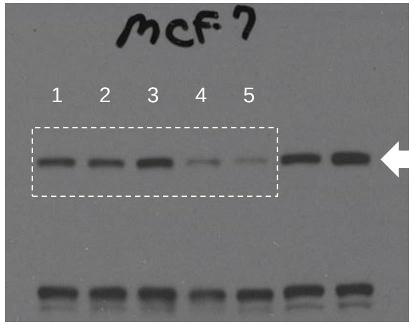

Eg5 band at ~120 kDa

Anti-cleaved PARP

Mouse anti-cl-PARP antibody
(1:500; BD Pharmingen, Cat# 552597)

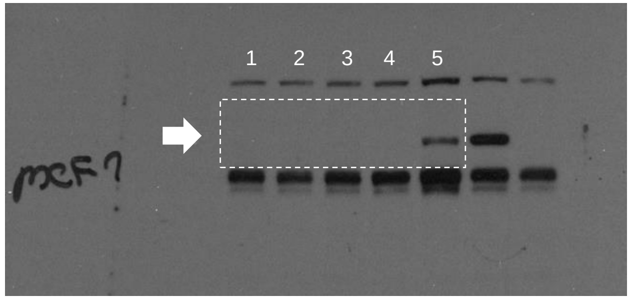

Cl-PARP band at ~89 kDa

Anti-MCL-1

Rabbit anti-MCL-1 antibody
(1:500; Cell Signaling, Cat# 5453)

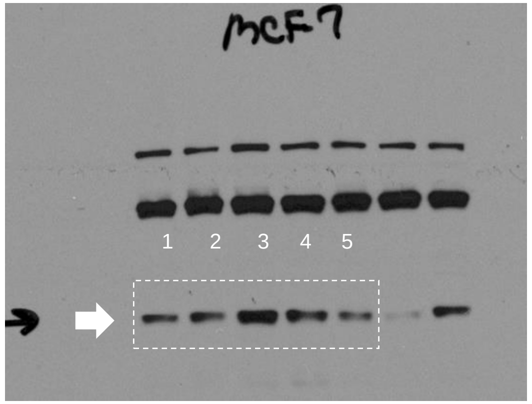

MCL-1 band at ~37 kDa

Anti-Cyclin B1

Mouse anti-Cyclin B1 antibody
(1:500; BD Pharmingen, Cat# 554179)

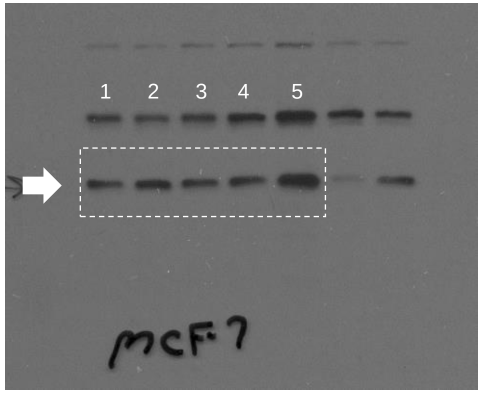

Cyclin B1 band at ~62 kDa

Anti-β-Actin

Mouse anti-β-Actin antibody
(1:5000; Sigma, Cat# A5441)

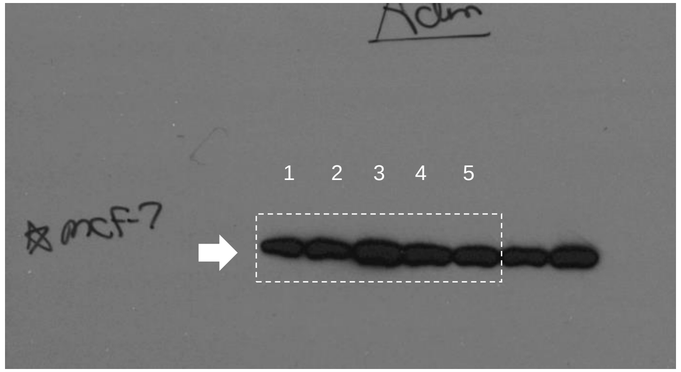

β-Actin band at ~42 kDa

Lane ID	siRNA
1	KIF18A[2]
2	KIF18A[4]
3	NTC[1]
4	NTC[2]
5	Eg5[1]
6	1 μM Staurosporine Control
7	Untreated Control

Lanes 6 & 7 cropped out

- Dashed rectangle box = cropped region
- Adjusted brightness equally (+20)

MDA-MB-453 Immunoblots, uncropped scans of film

Figure 1e

Anti-KIF18A

Rabbit anti-KIF18A antibody
(1:3000; Sigma, Cat# HPA 039484)

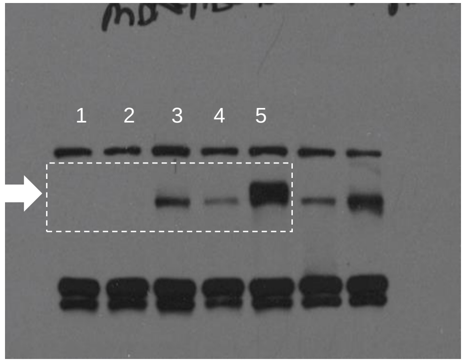

KIF18A band at ~110 kDa

Anti-Eg5

Mouse anti-Eg5 antibody
(1:2000; Abcam, Cat# ab51976)

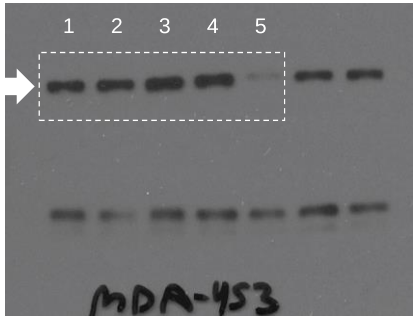

Eg5 band at ~120 kDa

Anti-cleaved PARP

Mouse anti-cl-PARP antibody
(1:500; BD Pharmingen, Cat# 552597)

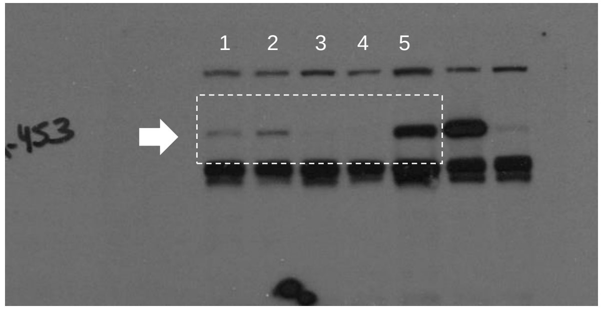

Cl-PARP band at ~89 kDa

Anti-MCL-1

Rabbit anti-MCL-1 antibody
(1:500; Cell Signaling, Cat# 5453)

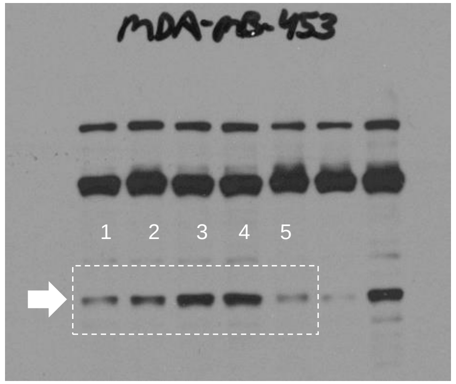

MCL-1 band at ~37 kDa

Anti-Cyclin B1

Mouse anti-Cyclin B1 antibody
(1:500; BD Pharmingen, Cat# 554179)

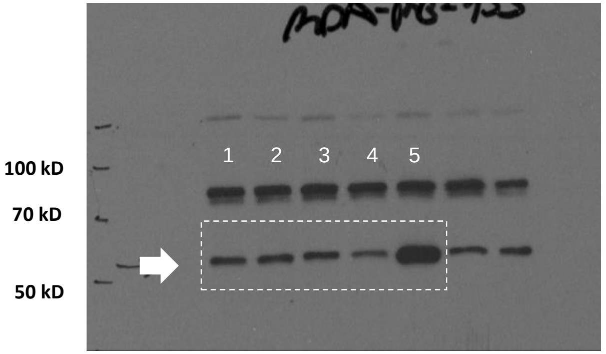

Cyclin B1 band at ~62 kDa

Anti-β-Actin

Mouse anti-β-Actin antibody
(1:5000; Sigma, Cat# A5441)

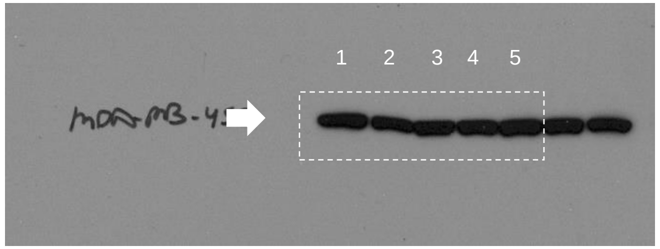

β-Actin band at ~42 kDa

Lane ID	siRNA
1	KIF18A[2]
2	KIF18A[4]
3	NTC[1]
4	NTC[2]
5	Eg5[1]
6	1 µM Staurosporine Control
7	Untreated Control

Lanes 6 & 7 cropped out

- Dashed rectangle box = cropped region
- Adjusted brightness equally (+20)

ZR-75-1 Immunoblots, uncropped scans of film

Figure 1e

Anti-KIF18A

Rabbit anti-KIF18A antibody
(1:3000; Sigma, Cat# HPA 039484)

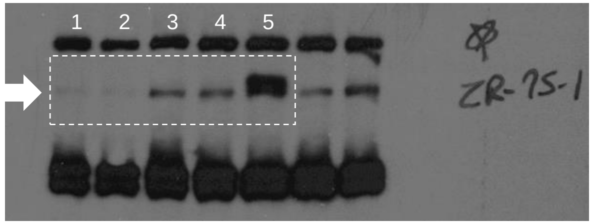

KIF18A band at ~110 kDa

Anti-Eg5

Mouse anti-Eg5 antibody
(1:2000; Abcam, Cat# ab51976)

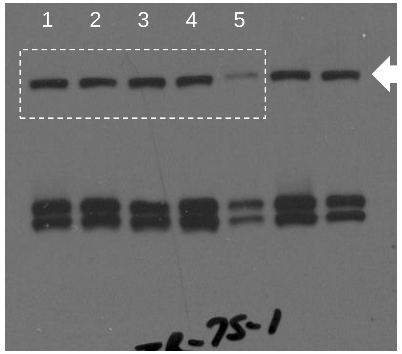

Eg5 band at ~120 kDa

Anti-cleaved PARP

Mouse anti-cl-PARP antibody
(1:500; BD Pharmingen, Cat# 552597)

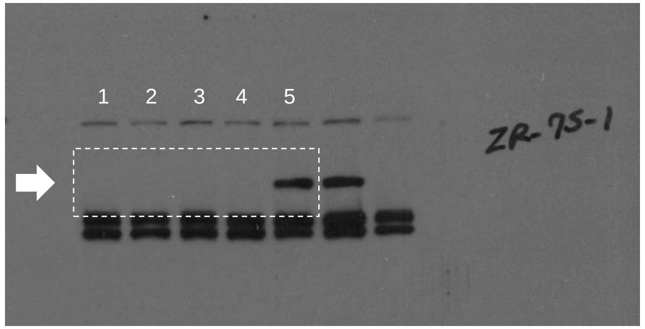

Cl-PARP band at ~89 kDa

Anti-MCL-1

Rabbit anti-MCL-1 antibody
(1:500; Cell Signaling, Cat# 5453)

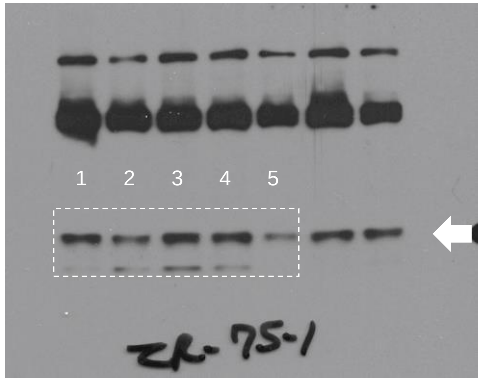

MCL-1 band at ~37 kDa

Anti-Cyclin B1

Mouse anti-Cyclin B1 antibody
(1:500; BD Pharmingen, Cat# 554179)

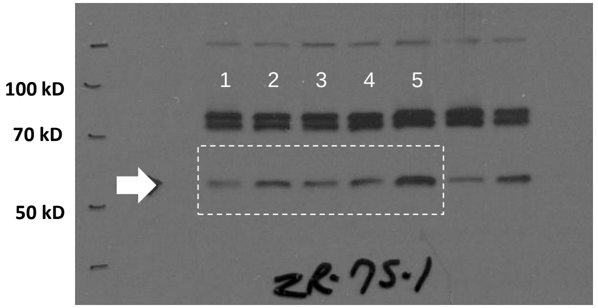

Cyclin B1 band at ~62 kDa

Anti-β-Actin

Mouse anti-β-Actin antibody
(1:5000; Sigma, Cat# A5441)

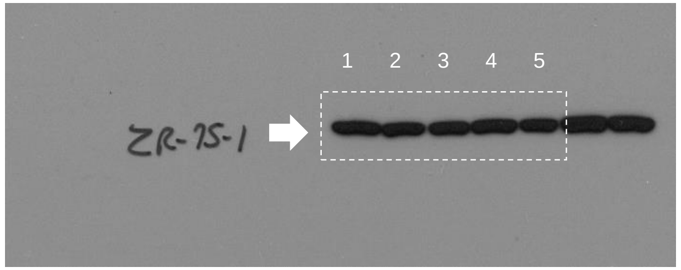

β-Actin band at ~42 kDa

Lane ID	siRNA
1	KIF18A[2]
2	KIF18A[4]
3	NTC[1]
4	NTC[2]
5	Eg5[1]
6	1 µM Staurosporine Control
7	Untreated Control

Lanes 6 & 7 cropped out

- Dashed rectangle box = cropped region
- Adjusted brightness equally (+20)

Figure 2f

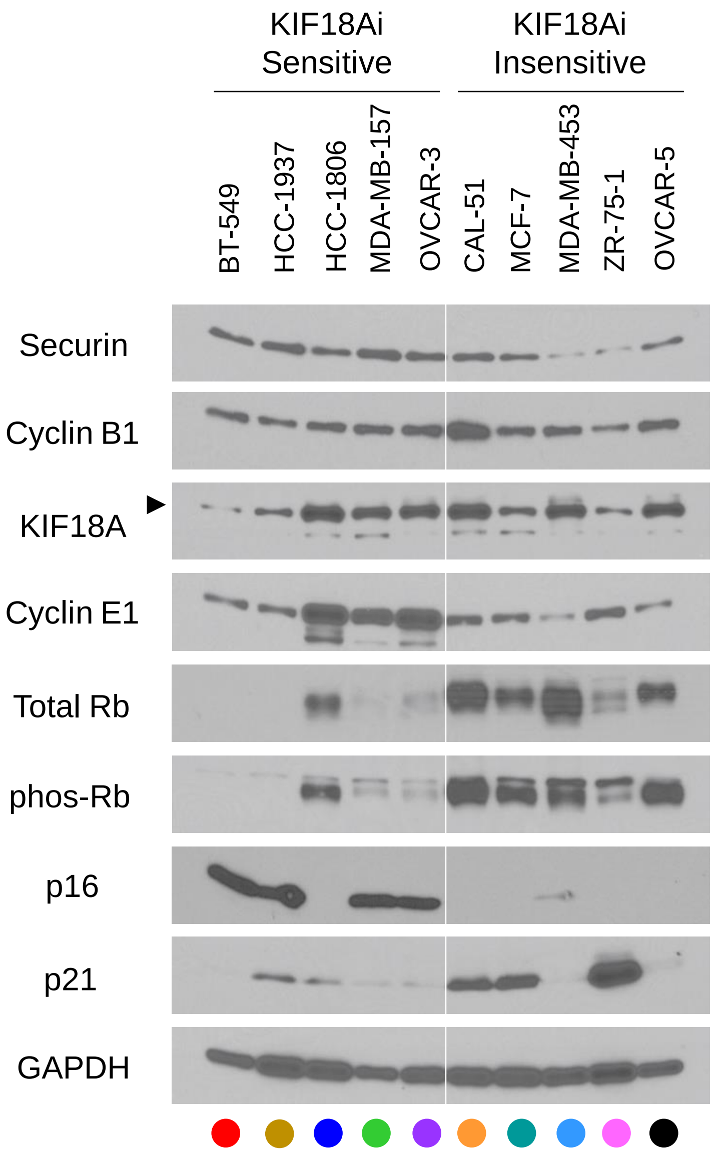

Adjusted brightness equally (+20)

Immunoblots, uncropped scans of film (Cell line panel, baseline expression)

Figure 2f

Anti-Securin

Rabbit anti-Securin antibody
(1:500; Abcam, Cat# ab79546)

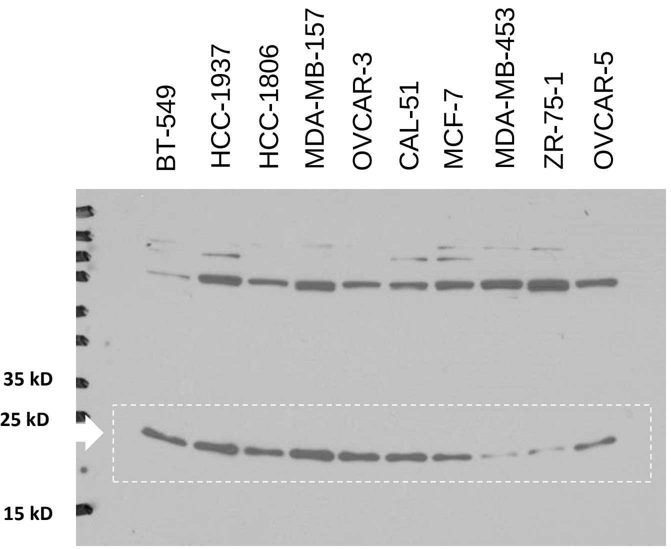

Securin band at ~25 kDa

Anti-Cyclin B1

Mouse anti-Cyclin B1 antibody
(1:500; BD Pharmingen, Cat# 554179)

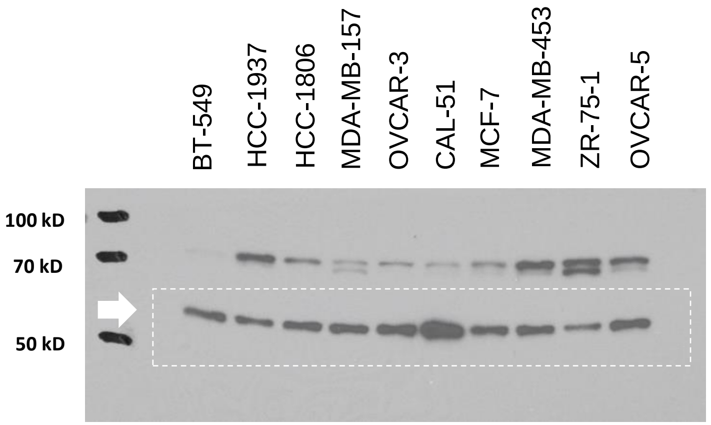

Cyclin B1 band at ~62 kDa

Anti-KIF18A

Rabbit anti-KIF18A antibody
(1:2000; Sigma, Cat# HPA039484)

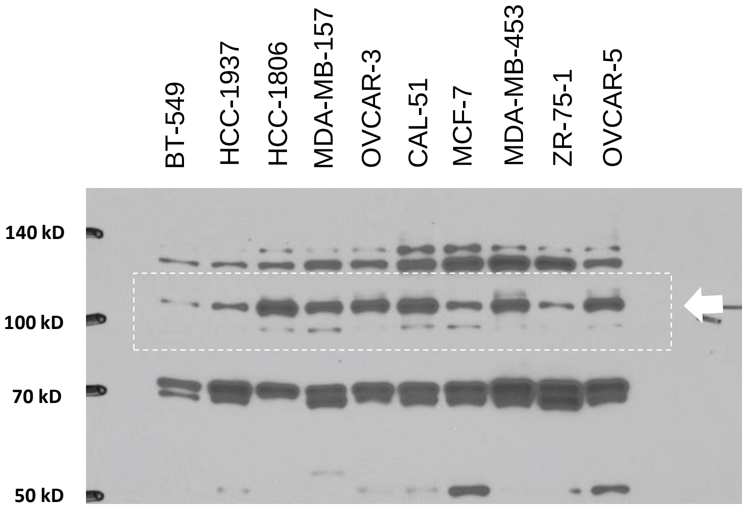

KIF18A band at ~110 kDa

- Dashed rectangle box = cropped region
- Adjusted brightness equally (+20)

Immunoblots, uncropped scans of film (Cell line panel, baseline expression)

Figure 2f

Anti-Cyclin E1

Mouse anti-Cyclin E1 antibody
(1:2000; Neomarkers, Cat# MS-870-P)

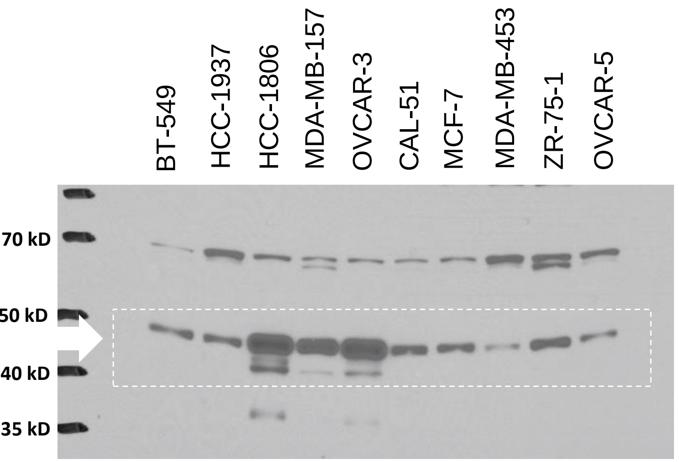

Cyclin E1 FL at ~50 kDa, multiple lower molecular weight (LMW) bands

Anti-Total Rb

Mouse anti-total Rb antibody
(1:300; BD Pharmingen, Cat# 554136)

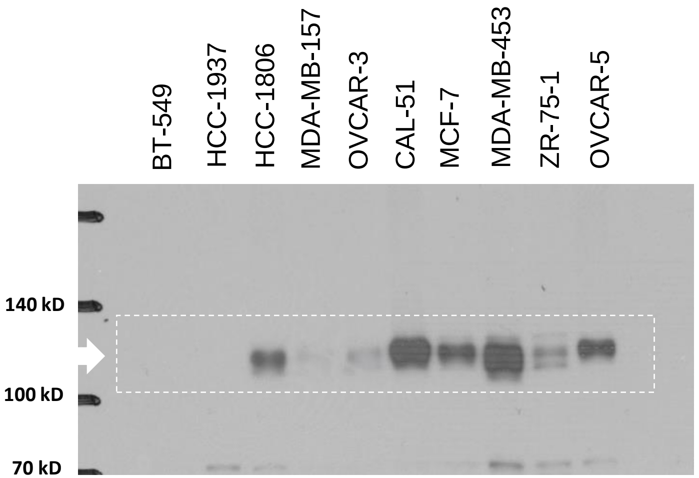

Rb at ~115 kDa, doublet band possible

Anti-phos-Rb

Rabbit anti-phospho-Rb (ser807/811) antibody
(1:1000; Cell Signaling, Cat# 9308s)

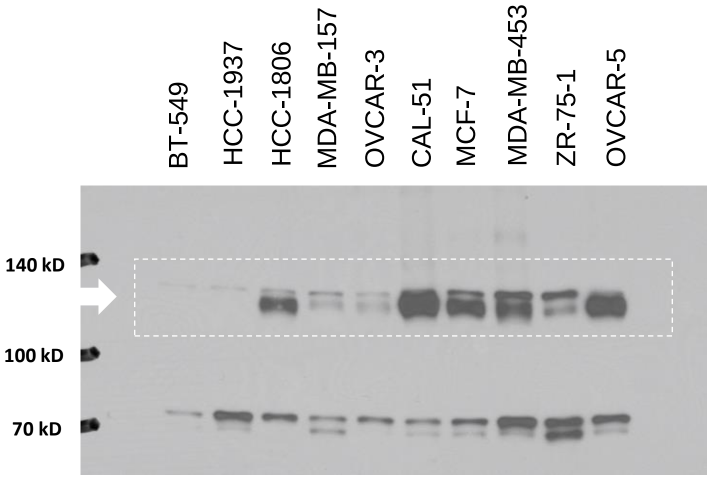

ppRb at ~115 kDa, doublet band possible

- Dashed rectangle box = cropped region
- Adjusted brightness equally (+20)

Immunoblots, uncropped scans of film (Cell line panel, baseline expression)

Figure 2f

Anti-p16

Mouse anti-p16 antibody
(1:500; BD Pharmingen, Cat# 554079)

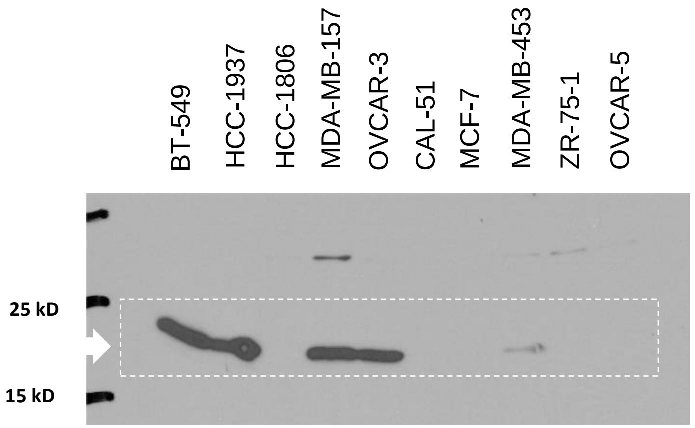

p16 band at ~16 kDa

Anti-p21

Rabbit anti-p21 antibody
(1:500; Cell Signaling, Cat# 2947s)

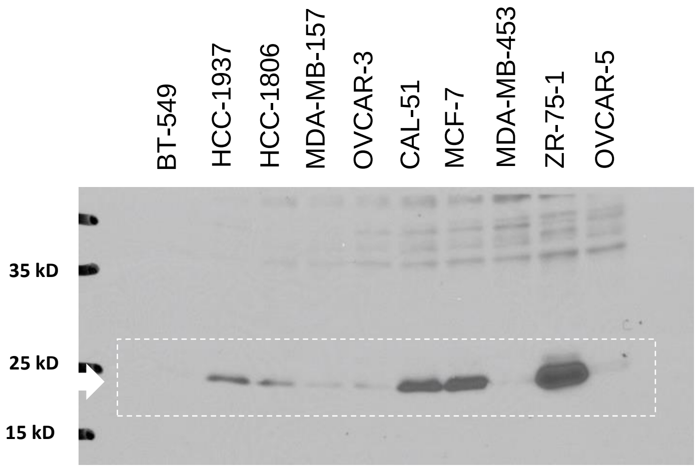

p21 band at ~21 kDa

Anti-GAPDH

Rabbit anti-GAPDH antibody
(1:10,000; Cell Signaling, Cat# 2118s)

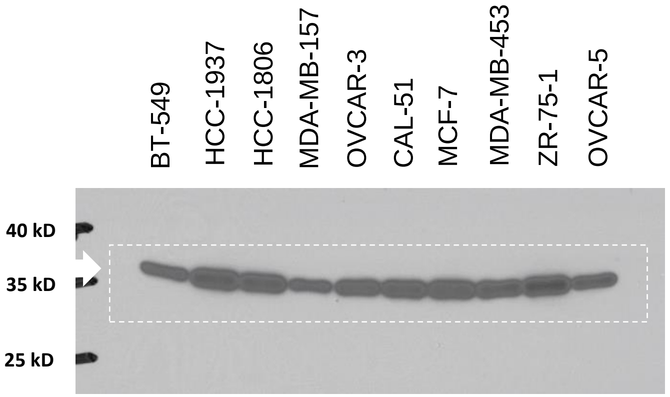

GAPDH band at ~37 kDa

- Dashed rectangle box = cropped region
- Adjusted brightness equally (+20)

Figure 2h

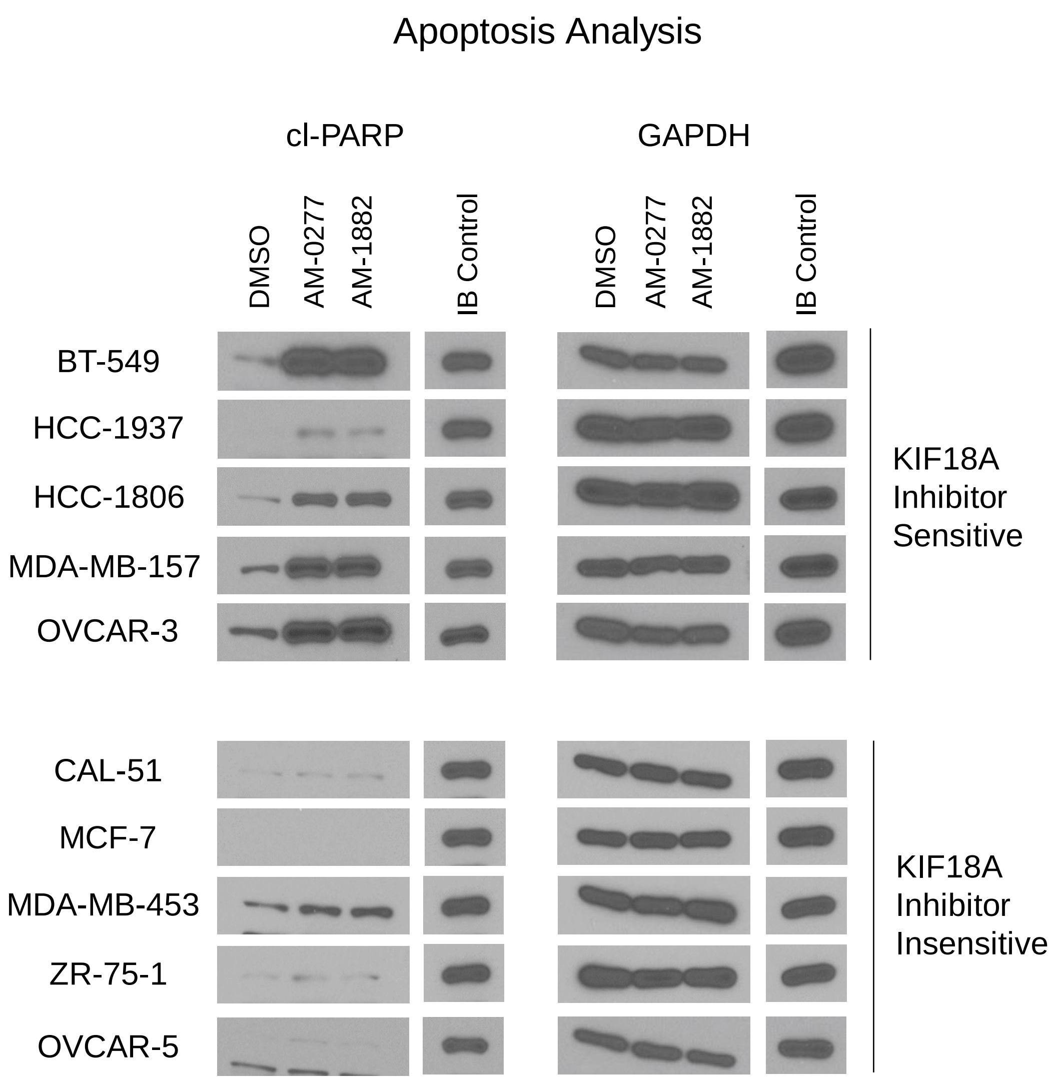

Adjusted brightness equally (+20)

BT-549 and HCC-1937 Immunoblots, uncropped scans of film AM-0277 and AM-1882

Figure 2h

Anti-cleaved PARP

Mouse anti-cl-PARP antibody
(1:500; BD Pharmingen, Cat# 552597)

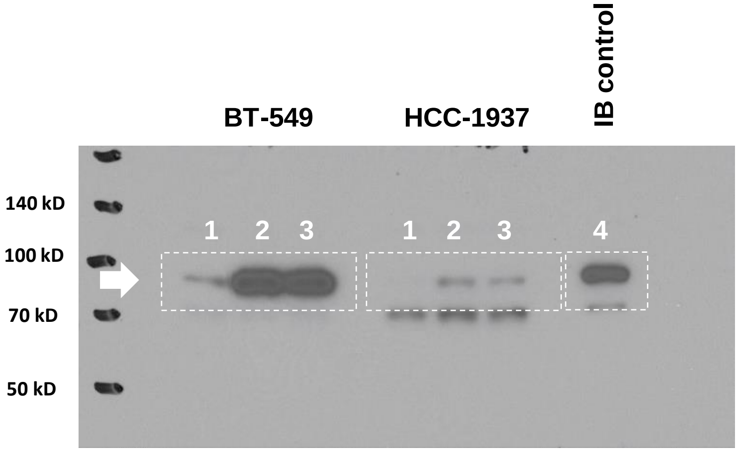

Cl-PARP band at ~89 kDa

Anti-GAPDH

Rabbit anti-GAPDH antibody
(1:10,000; Cell Signaling, 2118S)

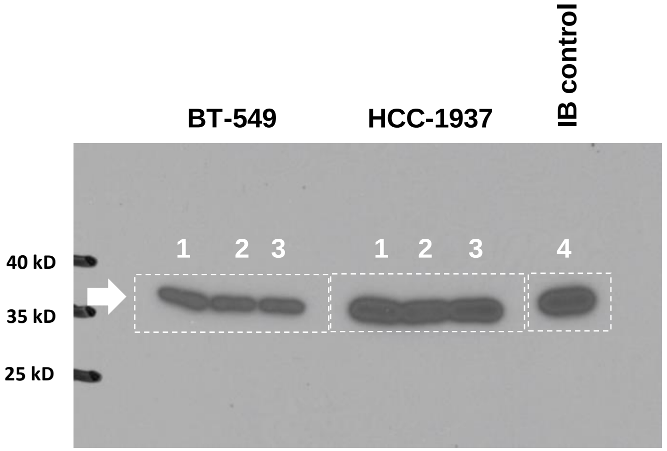

GAPDH band at ~37 kDa

Lane ID	BT-549 and HCC-1937 cells treated for 48 hours (lanes 1-3)
1	DMSO
2	AM-0277 (0.5 µM)
3	AM-1882 (0.1 µM)
	IB control, HCC-1806 cells treated for 48 hours
4	Ispinesib (0.05 µM)

IB = Immunoblot

- Dashed rectangle box = cropped region
- Adjusted brightness equally (+20)

HCC-1806 and MDA-MB-157 Immunoblots, uncropped scans of film AM-0277 and AM-1882

Figure 2h

Anti-cleaved PARP

Mouse anti-cl-PARP antibody
(1:500; BD Pharmingen, Cat# 552597)

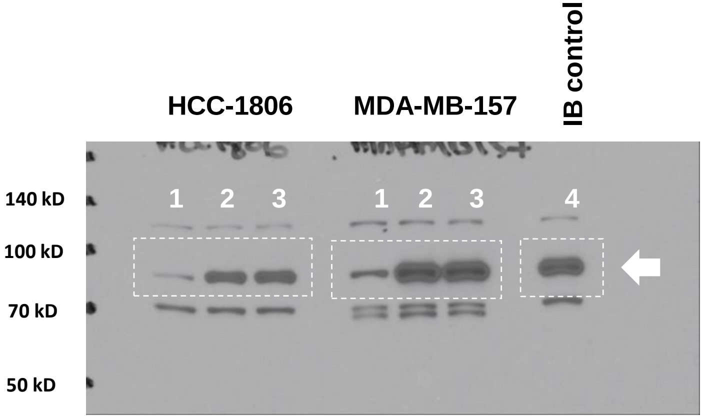

Cl-PARP band at ~89 kDa

Anti-GAPDH

Rabbit anti-GAPDH antibody
(1:10,000; Cell Signaling, 2118S)

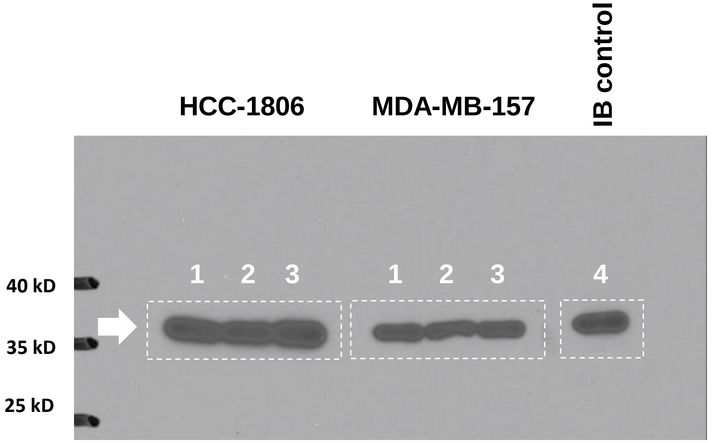

GAPDH band at ~37 kDa

Lane ID	HCC-1806 and MDA-MB-157 cells treated for 48 hours (lanes 1-3)
1	DMSO
2	AM-0277 (0.5 µM)
3	AM-1882 (0.1 µM)
	IB control, HCC-1806 cells treated for 48 hours
4	Ispinesib (0.05 µM)

IB = Immunoblot

- Dashed rectangle box = cropped region
- Adjusted brightness equally (+20)

OVCAR-3 Immunoblots, uncropped scans of film AM-0277 and AM-1882

Figure 2h

Anti-cleaved PARP

Mouse anti-cl-PARP antibody
(1:500; BD Pharmingen, Cat# 552597)

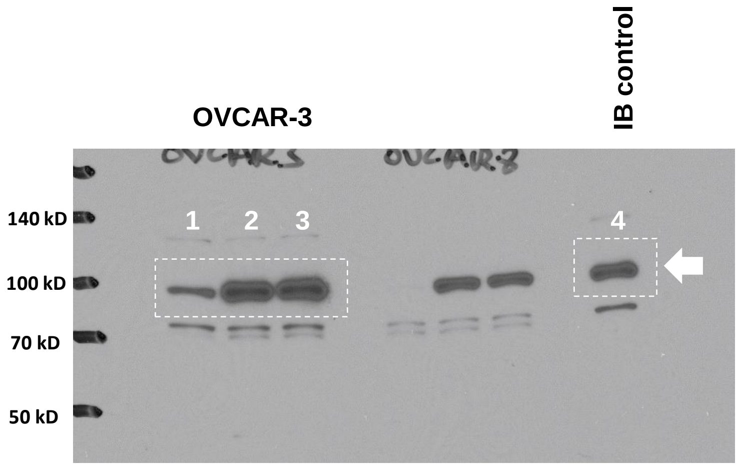

Cl-PARP band at ~89 kDa

Anti-GAPDH

Rabbit anti-GAPDH antibody
(1:10,000; Cell Signaling, 2118S)

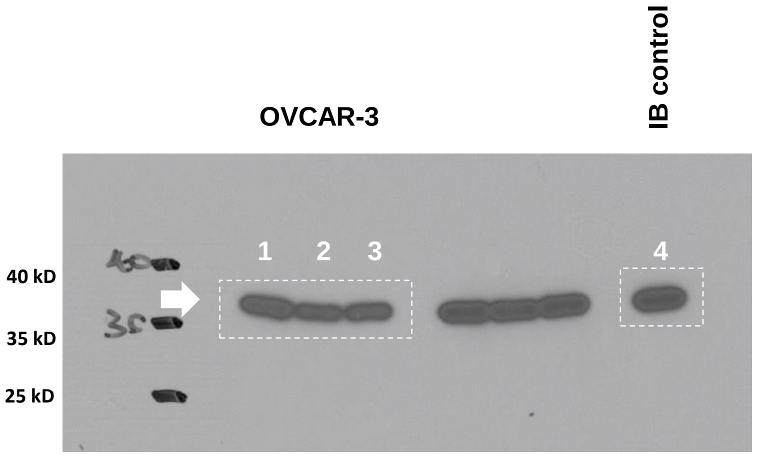

GAPDH band at ~37 kDa

Lane ID	OVCAR-3 treated for 48 hours (lanes 1-3)
1	DMSO
2	AM-0277 (0.5 µM)
3	AM-1882 (0.1 µM)
	IB control, HCC-1806 cells treated for 48 hours
4	Ispinesib (0.05 µM)

IB = Immunoblot

- Dashed rectangle box = cropped region
- Adjusted brightness equally (+20)

CAL-51 and MCF-7 Immunoblots, uncropped scans of film AM-0277 and AM-1882

Figure 2h

Anti-cleaved PARP

Mouse anti-cl-PARP antibody
(1:500; BD Pharmingen, Cat# 552597)

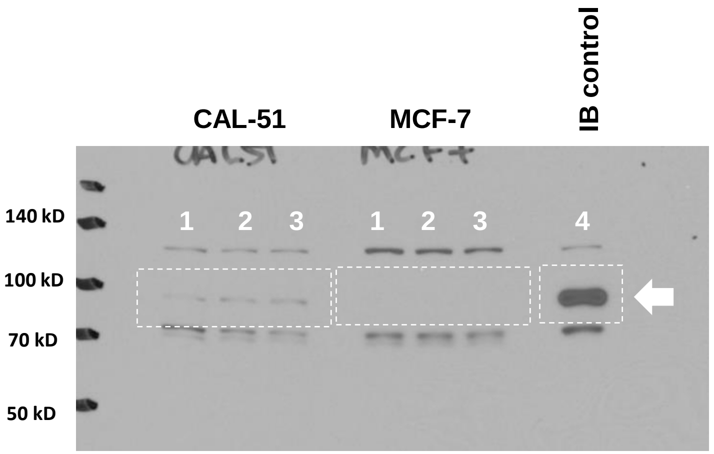

Cl-PARP band at ~89 kDa

Anti-GAPDH

Rabbit anti-GAPDH antibody
(1:10,000; Cell Signaling, 2118S)

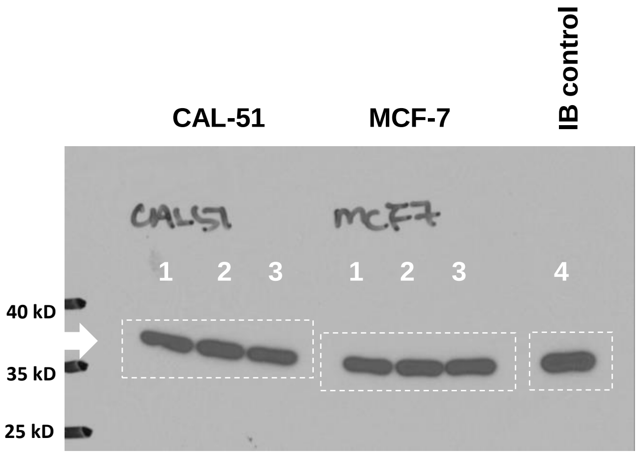

GAPDH band at ~37 kDa

Lane ID	CAL-51 and MCF-7 cells treated for 48 hours (lanes 1-3)
1	DMSO
2	AM-0277 (0.5 µM)
3	AM-1882 (0.1 µM)
	IB control, HCC-1806 cells treated for 48 hours
4	Ispinesib (0.05 µM)

IB = Immunoblot

- Dashed rectangle box = cropped region
- Adjusted brightness equally (+20)

MDA-MB-453 and ZR-75-1 Immunoblots, uncropped scans of film AM-0277 and AM-1882

Figure 2h

Anti-cleaved PARP

Mouse anti-cl-PARP antibody
(1:500; BD Pharmingen, Cat# 552597)

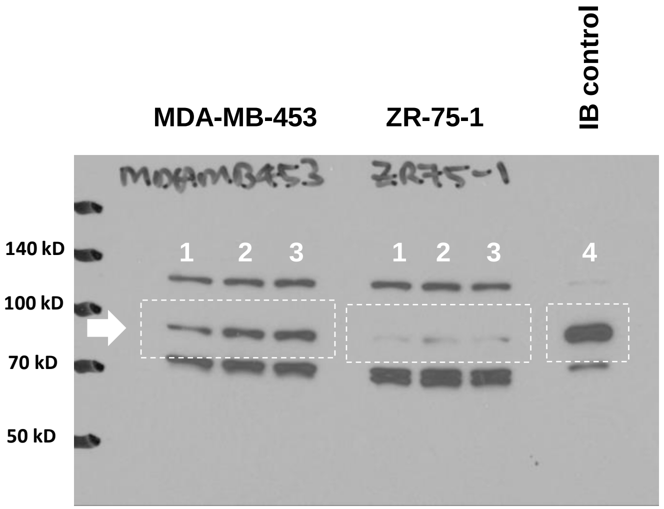

Cl-PARP band at ~89 kDa

Anti-GAPDH

Rabbit anti-GAPDH antibody
(1:10,000; Cell Signaling, 2118S)

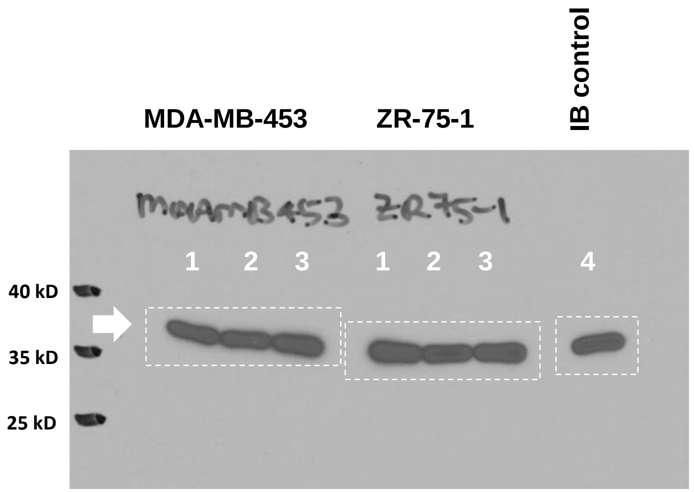

GAPDH band at ~37 kDa

Lane ID	MDA-MB-453 and ZR-75-1 cells treated for 48 hours (lanes 1-3)
1	DMSO
2	AM-0277 (0.5 µM)
3	AM-1882 (0.1 µM)
	IB control, HCC-1806 cells treated for 48 hours
4	Ispinesib (0.05 µM)

IB = Immunoblot

- Dashed rectangle box = cropped region
- Adjusted brightness equally (+20)

OVCAR-5 Immunoblots, uncropped scans of film AM-0277 and AM-1882

Figure 2h

Anti-cleaved PARP

Mouse anti-cl-PARP antibody
(1:500; BD Pharmingen, Cat# 552597)

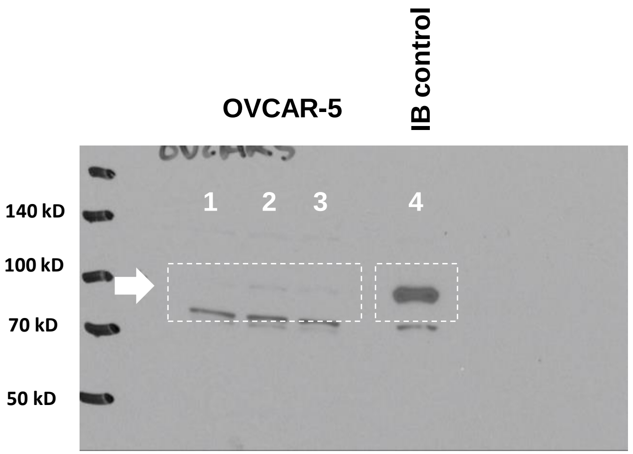

Cl-PARP band at ~89 kDa

Anti-GAPDH

Rabbit anti-GAPDH antibody
(1:10,000; Cell Signaling, 2118S)

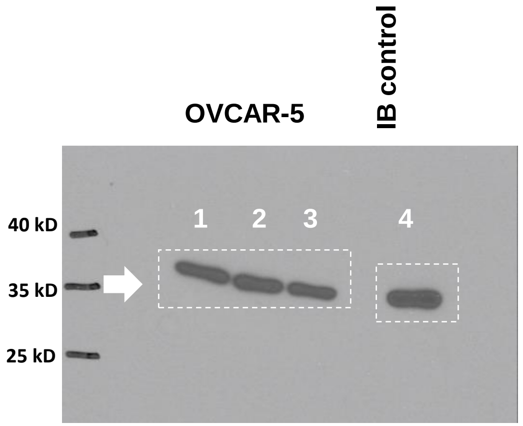

GAPDH band at ~37 kDa

Lane ID	OVCAR-5 cells treated for 48 hours (lanes 1-3)
1	DMSO
2	AM-0277 (0.5 µM)
3	AM-1882 (0.1 µM)
	IB control, HCC-1806 cells treated for 48 hours
4	Ispinesib (0.05 µM)

IB = Immunoblot

- Dashed rectangle box = cropped region
- Adjusted brightness equally (+20)

Extended Data Figure 1a

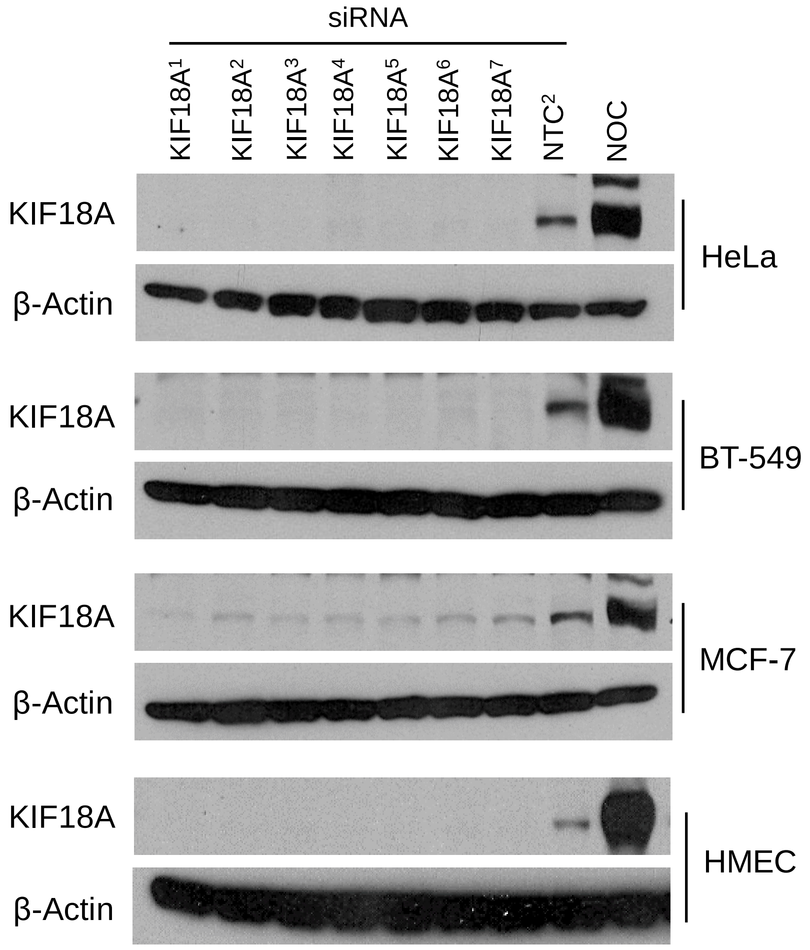

Adjusted brightness equally (+20)

Extended Data Figure 1a

Immunoblots uncropped scans of film HeLa

Anti-KIF18A

Rabbit anti-KIF18A antibody
(1:3000; Sigma, Cat# HPA 039484)

1 2 3 4 5 6 7 8 9

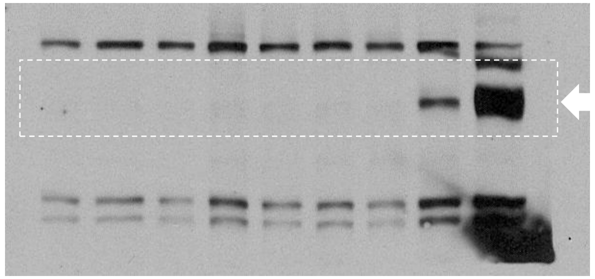

KIF18A band at ~110 kDa

Anti-β-Actin

Mouse anti-β-Actin antibody
(1:5000; Sigma, Cat# A5441)

1 2 3 4 5 6 7 8 9

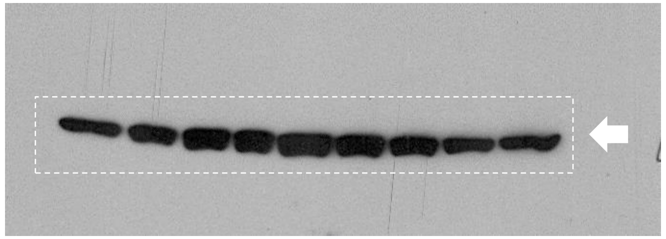

β-Actin band at ~42 kDa

Immunoblots uncropped scans of film BT-549

Anti-KIF18A

Rabbit anti-KIF18A antibody
(1:3000; Sigma, Cat# HPA 039484)

1 2 3 4 5 6 7 8 9

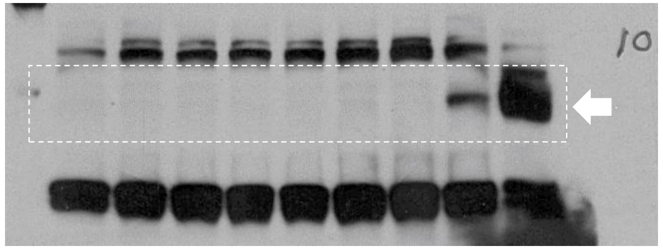

KIF18A band at ~110 kDa

Anti-β-Actin

Mouse anti-β-Actin antibody
(1:5000; Sigma, Cat# A5441)

1 2 3 4 5 6 7 8 9

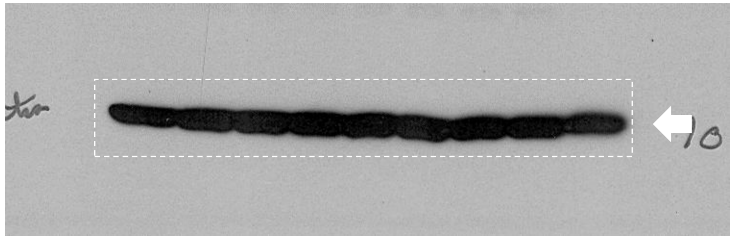

β-Actin band at ~42 kDa

Lane ID	siRNA
1	KIF18A[1]
2	KIF18A[2]
3	KIF18A[3]
4	KIF18A[4]
5	KIF18A[5]
6	KIF18A[6]
7	KIF18A[7]
8	NTC[2]
9	HeLa Nocodazole Control

- Dashed rectangle box = cropped region
- Adjusted brightness equally (+20)

Extended Data Figure 1a

Immunoblots uncropped scans of film MCF-7

Anti-KIF18A

Rabbit anti-KIF18A antibody
(1:3000; Sigma, Cat# HPA 039484)

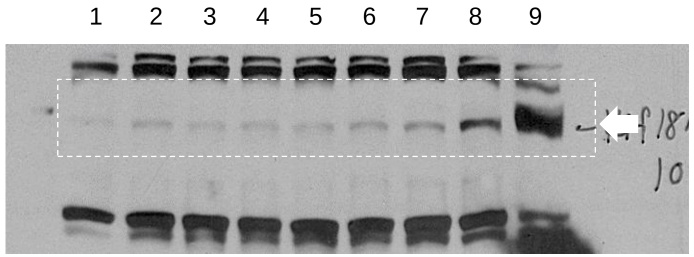

KIF18A band at ~110 kDa

Anti-β-Actin

Mouse anti-β-Actin antibody
(1:5000; Sigma, Cat# A5441)

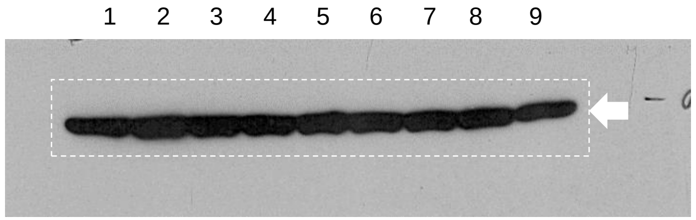

β-Actin band at ~42 kDa

Immunoblots uncropped scans of film HMEC

Anti-KIF18A

Rabbit anti-KIF18A antibody
(1:3000; Sigma, Cat# HPA 039484)

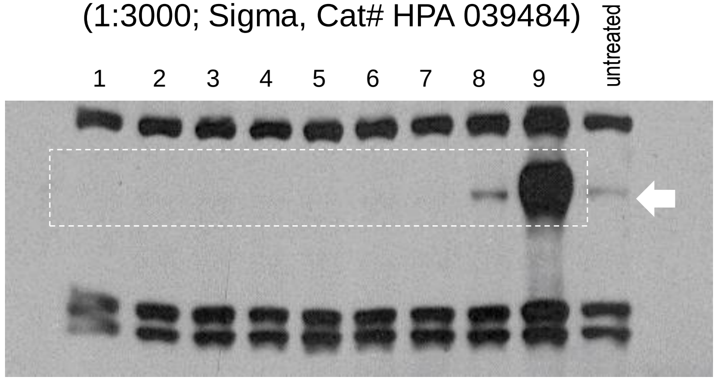

KIF18A band at ~110 kDa

Anti-β-Actin

Mouse anti-β-Actin antibody
(1:5000; Sigma, Cat# A5441)

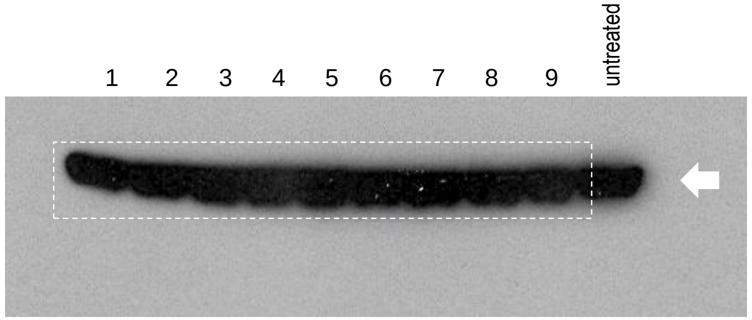

β-Actin band at ~42 kDa

Lane ID	siRNA
1	KIF18A^{1}
2	KIF18A^{2}
3	KIF18A^{3}
4	KIF18A^{4}
5	KIF18A^{5}
6	KIF18A^{6}
7	KIF18A^{7}
8	NTC^{2}
9	HeLa Nocodazole Control

Untreated control included in HMEC experiment

- Dashed rectangle box = cropped region
- Adjusted brightness equally (+20)

Extended Data Figure 4b

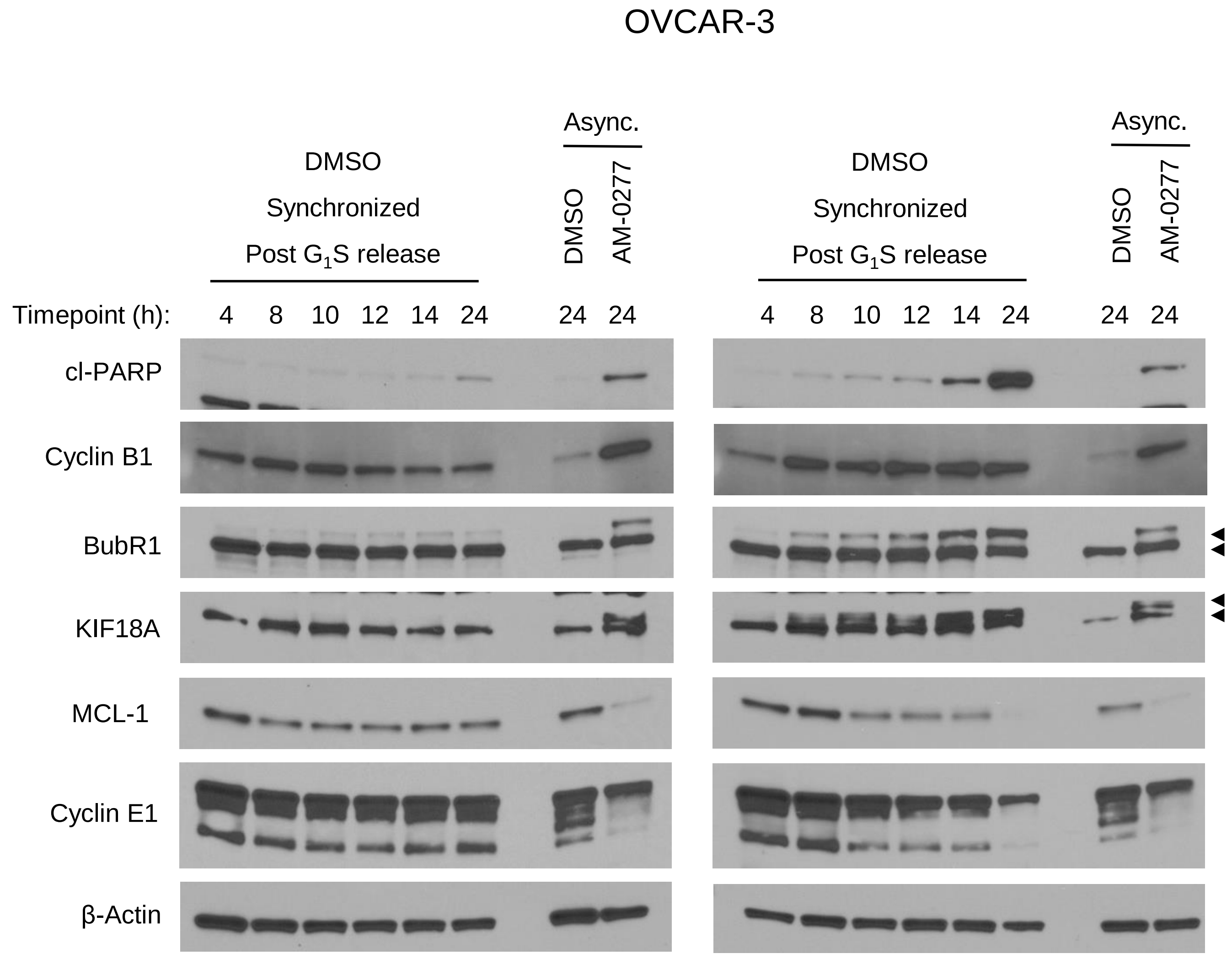

Adjusted brightness equally (+20)

Immunoblots uncropped scans of film, OVCAR-3 Double Thymidine-block and release (Anti-cl-PARP)

Extended Data Figure 4b

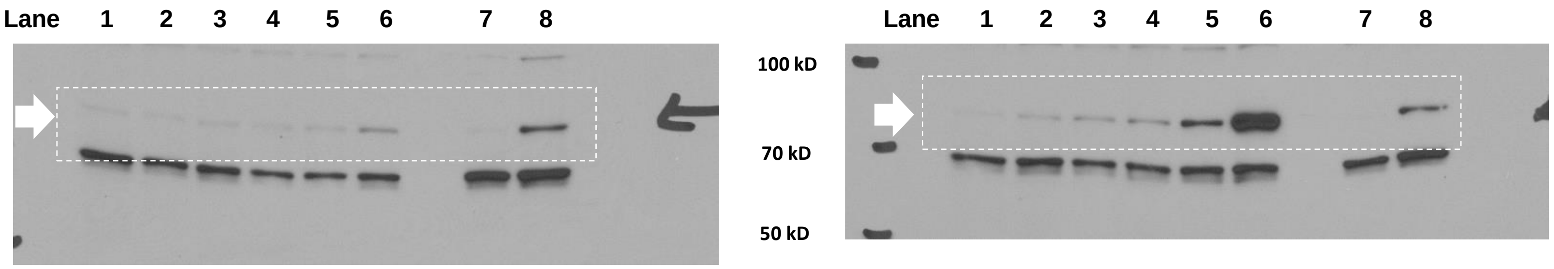

Cl-PARP band at ~89 kDa

Anti-cleaved PARP

Mouse anti-cl-PARP antibody
(1:500; BD Pharmingen, Cat# 552597)

	Treatment	Timepoint
Lane 1	DMSO	4 hours post G1S release
Lane 2	DMSO	8 hours post G1S release
Lane 3	DMSO	10 hours post G1S release
Lane 4	DMSO	12 hours post G1S release
Lane 5	DMSO	14 hours post G1S release
Lane 6	DMSO	24 hours post G1S release
Space		
Lane 7	DMSO	24 hours asynchronous
Lane 8	AM-0277	24 hours asynchronous

	Treatment	Timepoint
Lane 1	AM-0277	4 hours post G1S release
Lane 2	AM-0277	8 hours post G1S release
Lane 3	AM-0277	10 hours post G1S release
Lane 4	AM-0277	12 hours post G1S release
Lane 5	AM-0277	14 hours post G1S release
Lane 6	AM-0277	24 hours post G1S release
Space		
Lane 7	DMSO	24 hours asynchronous
Lane 8	AM-0277	24 hours asynchronous

- Dashed rectangle box = cropped region
- Adjusted brightness equally (+20)

Immunoblots uncropped scans of film, OVCAR-3 Double Thymidine-block and release (Anti-Cyclin B1)

Extended Data Figure 4b

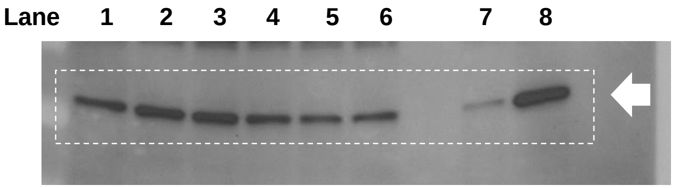

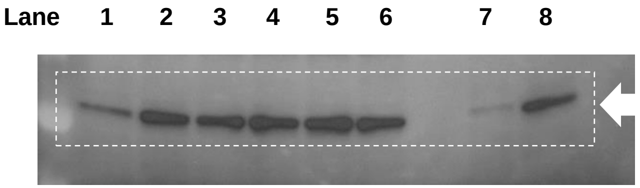

Anti-Cyclin B1

Mouse anti-Cyclin B1 antibody
(1:500; BD Pharmingen, Cat# 554179)

Cyclin B1 band at ~62 kDa

	Treatment	Timepoint
Lane 1	DMSO	4 hours post G1S release
Lane 2	DMSO	8 hours post G1S release
Lane 3	DMSO	10 hours post G1S release
Lane 4	DMSO	12 hours post G1S release
Lane 5	DMSO	14 hours post G1S release
Lane 6	DMSO	24 hours post G1S release
Space		
Lane 7	DMSO	24 hours asynchronous
Lane 8	AM-0277	24 hours asynchronous

	Treatment	Timepoint
Lane 1	AM-0277	4 hours post G1S release
Lane 2	AM-0277	8 hours post G1S release
Lane 3	AM-0277	10 hours post G1S release
Lane 4	AM-0277	12 hours post G1S release
Lane 5	AM-0277	14 hours post G1S release
Lane 6	AM-0277	24 hours post G1S release
Space		
Lane 7	DMSO	24 hours asynchronous
Lane 8	AM-0277	24 hours asynchronous

- Dashed rectangle box = cropped region
- Adjusted brightness equally (+20)

Immunoblots uncropped scans of film, OVCAR-3 Double Thymidine-block and release (Anti-BubR1)

Extended Data Figure 4b

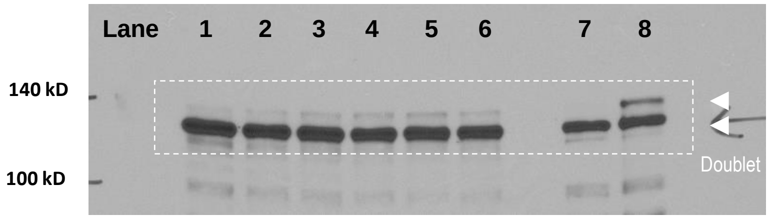

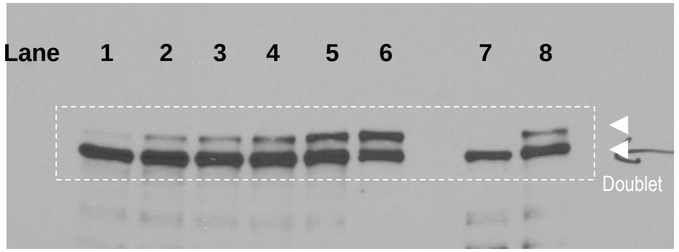

Anti-BubR1

Mouse anti-BubR1 antibody
(1:5000; BD Pharmingen, Cat# 612503)

BubR1 band single or doublet at ~ 125 kDa

	Treatment	Timepoint
Lane 1	DMSO	4 hours post G1S release
Lane 2	DMSO	8 hours post G1S release
Lane 3	DMSO	10 hours post G1S release
Lane 4	DMSO	12 hours post G1S release
Lane 5	DMSO	14 hours post G1S release
Lane 6	DMSO	24 hours post G1S release
Space		
Lane 7	DMSO	24 hours asynchronous
Lane 8	AM-0277	24 hours asynchronous

	Treatment	Timepoint
Lane 1	AM-0277	4 hours post G1S release
Lane 2	AM-0277	8 hours post G1S release
Lane 3	AM-0277	10 hours post G1S release
Lane 4	AM-0277	12 hours post G1S release
Lane 5	AM-0277	14 hours post G1S release
Lane 6	AM-0277	24 hours post G1S release
Space		
Lane 7	DMSO	24 hours asynchronous
Lane 8	AM-0277	24 hours asynchronous

- Dashed rectangle box = cropped region
- Adjusted brightness equally (+20)

Immunoblots uncropped scans of film, OVCAR-3 Double Thymidine-block and release (Anti-KIF18A)

Extended Data Figure 4b

Lane 1 2 3 4 5 6 7 8

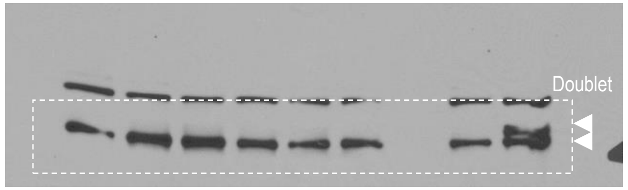

Lane 1 2 3 4 5 6 7 8

140 kD

100 kD

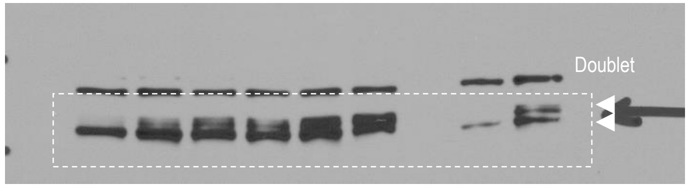

Anti-KIF18A

Rabbit anti-KIF18A antibody
(1:3000; Sigma, Cat# HPA 039484)

KIF18A band single or doublet at ~ 110 kDa

	Treatment	Timepoint
Lane 1	DMSO	4 hours post G1S release
Lane 2	DMSO	8 hours post G1S release
Lane 3	DMSO	10 hours post G1S release
Lane 4	DMSO	12 hours post G1S release
Lane 5	DMSO	14 hours post G1S release
Lane 6	DMSO	24 hours post G1S release
Space		
Lane 7	DMSO	24 hours asynchronous
Lane 8	AM-0277	24 hours asynchronous

	Treatment	Timepoint
Lane 1	AM-0277	4 hours post G1S release
Lane 2	AM-0277	8 hours post G1S release
Lane 3	AM-0277	10 hours post G1S release
Lane 4	AM-0277	12 hours post G1S release
Lane 5	AM-0277	14 hours post G1S release
Lane 6	AM-0277	24 hours post G1S release
Space		
Lane 7	DMSO	24 hours asynchronous
Lane 8	AM-0277	24 hours asynchronous

- Dashed rectangle box = cropped region
- Adjusted brightness equally (+20)

Immunoblots uncropped scans of film, OVCAR-3 Double Thymidine-block and release (Anti-MCL-1)

Extended Data Figure 4b

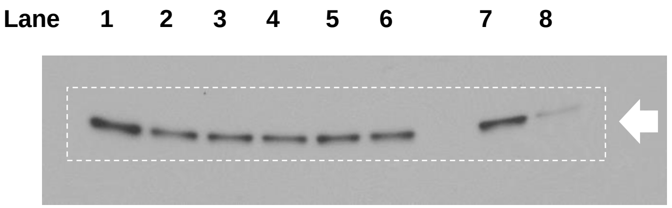

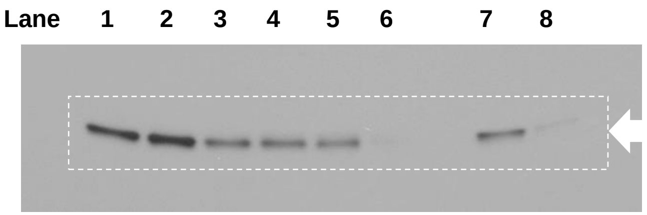

Anti-MCL-1

Rabbit anti-MCL-1 antibody
(1:500; Cell Signaling, Cat# 5453)

MCL-1 band at ~37 kDa

	Treatment	Timepoint
Lane 1	DMSO	4 hours post G1S release
Lane 2	DMSO	8 hours post G1S release
Lane 3	DMSO	10 hours post G1S release
Lane 4	DMSO	12 hours post G1S release
Lane 5	DMSO	14 hours post G1S release
Lane 6	DMSO	24 hours post G1S release
Space		
Lane 7	DMSO	24 hours asynchronous
Lane 8	AM-0277	24 hours asynchronous

	Treatment	Timepoint
Lane 1	AM-0277	4 hours post G1S release
Lane 2	AM-0277	8 hours post G1S release
Lane 3	AM-0277	10 hours post G1S release
Lane 4	AM-0277	12 hours post G1S release
Lane 5	AM-0277	14 hours post G1S release
Lane 6	AM-0277	24 hours post G1S release
Space		
Lane 7	DMSO	24 hours asynchronous
Lane 8	AM-0277	24 hours asynchronous

- Dashed rectangle box = cropped region
- Adjusted brightness equally (+20)

Immunoblots uncropped scans of film, OVCAR-3 Double Thymidine-block and release (Anti-Cyclin E1)

Extended Data Figure 4b

Anti-Cyclin E1

Mouse anti-Cyclin E1 antibody
(1:2000; Neomarkers, Cat# MS-870-P)

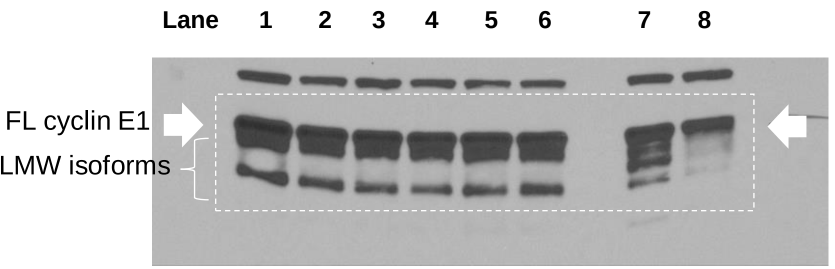

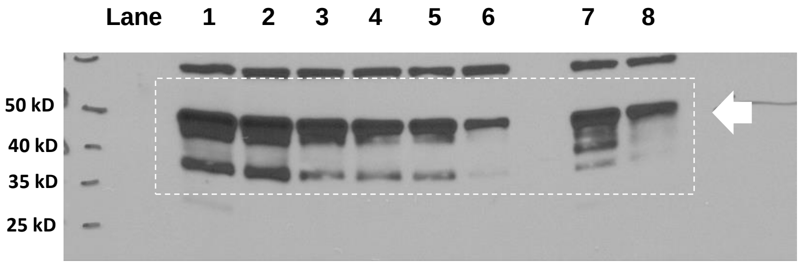

Cyclin E1 FL band at ~50 kDa, multiple lower molecular weight (LMW) isoform bands

	Treatment	Timepoint
Lane 1	DMSO	4 hours post G1S release
Lane 2	DMSO	8 hours post G1S release
Lane 3	DMSO	10 hours post G1S release
Lane 4	DMSO	12 hours post G1S release
Lane 5	DMSO	14 hours post G1S release
Lane 6	DMSO	24 hours post G1S release
Space		
Lane 7	DMSO	24 hours asynchronous
Lane 8	AM-0277	24 hours asynchronous

	Treatment	Timepoint
Lane 1	AM-0277	4 hours post G1S release
Lane 2	AM-0277	8 hours post G1S release
Lane 3	AM-0277	10 hours post G1S release
Lane 4	AM-0277	12 hours post G1S release
Lane 5	AM-0277	14 hours post G1S release
Lane 6	AM-0277	24 hours post G1S release
Space		
Lane 7	DMSO	24 hours asynchronous
Lane 8	AM-0277	24 hours asynchronous

- Dashed rectangle box = cropped region
- Adjusted brightness equally (+20)

Immunoblots uncropped scans of film, OVCAR-3 Double Thymidine-block and release (Anti-β-Actin, loading control)

Extended Data Figure 4b

Lane 1 2 3 4 5 6 7 8

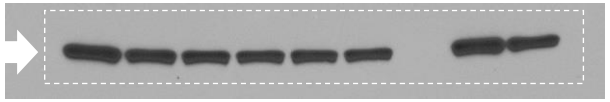

Lane 1 2 3 4 5 6 7 8

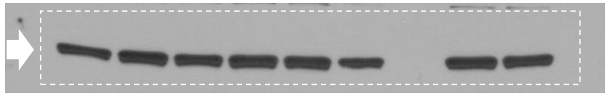

Anti-β-Actin

Mouse anti-β-Actin antibody (1:5000; Sigma, Cat# A5441)

β-Actin band at ~42 kDa

	Treatment	Timepoint
Lane 1	DMSO	4 hours post G1S release
Lane 2	DMSO	8 hours post G1S release
Lane 3	DMSO	10 hours post G1S release
Lane 4	DMSO	12 hours post G1S release
Lane 5	DMSO	14 hours post G1S release
Lane 6	DMSO	24 hours post G1S release
Space		
Lane 7	DMSO	24 hours asynchronous
Lane 8	AM-0277	24 hours asynchronous

	Treatment	Timepoint
Lane 1	AM-0277	4 hours post G1S release
Lane 2	AM-0277	8 hours post G1S release
Lane 3	AM-0277	10 hours post G1S release
Lane 4	AM-0277	12 hours post G1S release
Lane 5	AM-0277	14 hours post G1S release
Lane 6	AM-0277	24 hours post G1S release
Space		
Lane 7	DMSO	24 hours asynchronous
Lane 8	AM-0277	24 hours asynchronous

- Dashed rectangle box = cropped region
- Adjusted brightness equally (+20)

Extended Data Figure 4d

BT-549 Immunoblots uncropped scans of film AM-0277 and AM-1882

BT-549

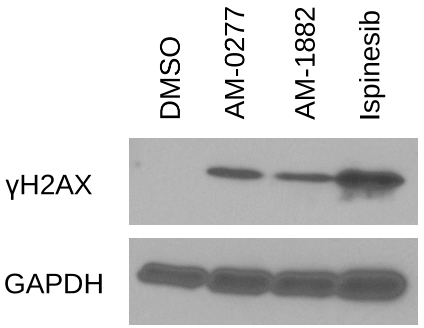

Lane ID	BT-549<br>treated for 48 hours (lanes 1-4)
1	DMSO
2	AM-0277 (0.5 µM)
3	AM-1882 (0.1 µM)
4	Ispinesib (0.05 µM)
5	Nocodazole (0.1 ug/mL)

Anti-γH2AX

Mouse Anti-Histone H2A.X
(phospho Ser-139), antibody
(1:2000; Millipore, Cat# 05-636)

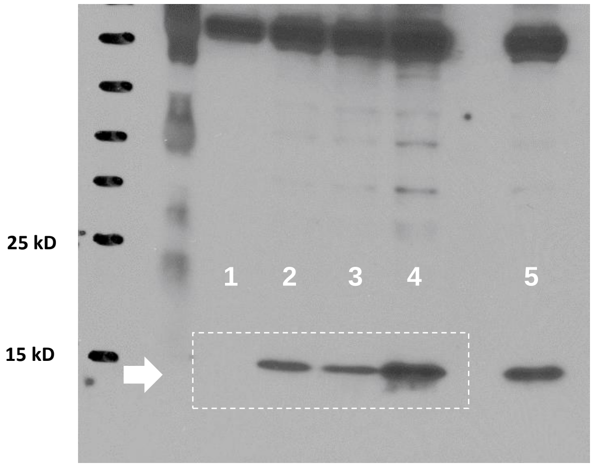

Phospho-γH2AX band at ~17 kDa

Anti-GAPDH

Rabbit anti-GAPDH antibody
(1:10,000; Cell Signaling, 2118S)

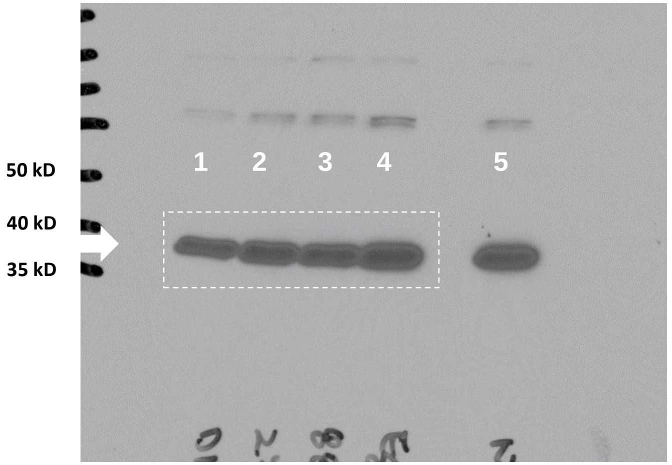

GAPDH band at ~37 kDa

- Dashed rectangle box = cropped region
- Adjusted brightness equally (+20)

Extended Data Figure 4h

OVCAR-8 paired lines Immunoblots, uncropped scans of film AM-0277 and AM-1882

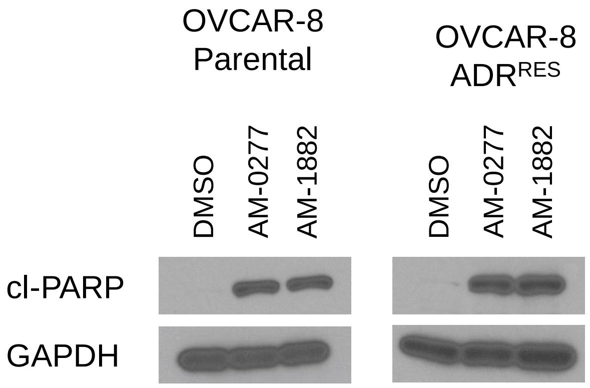

Lane ID	OVCAR-8 and OVCAR-8 cells treated for 48 hours (lanes 1-3)
1	DMSO
2	AM-0277 (0.5 µM)
3	AM-1882 (0.1 µM)
	IB control, HCC-1806 cells treated for 48 hours
4	Ispinesib (0.05 µM)

IB = Immunoblot

Anti-cleaved PARP

Mouse anti-cl-PARP antibody
(1:500; BD Pharmingen, Cat# 552597)

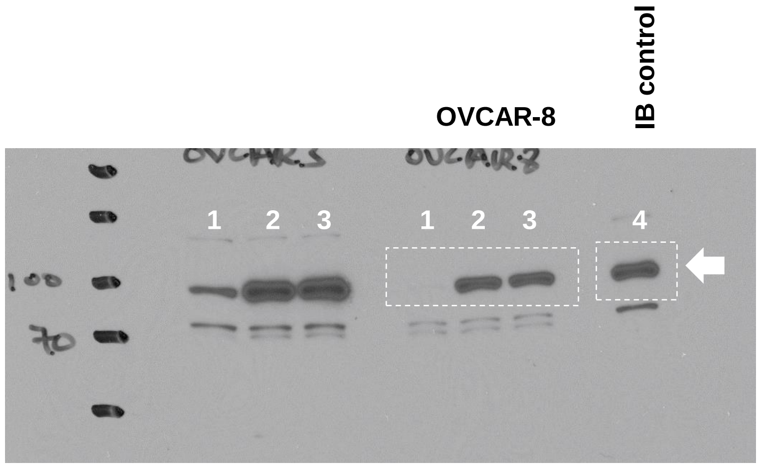

Cl-PARP band at ~89 kDa

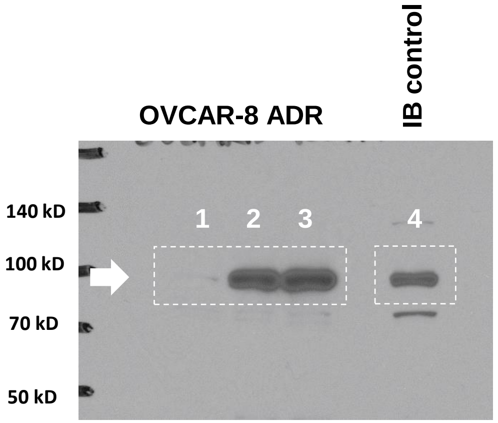

Cl-PARP band at ~89 kDa

Anti-GAPDH

Rabbit anti-GAPDH antibody
(1:10,000; Cell Signaling, 2118S)

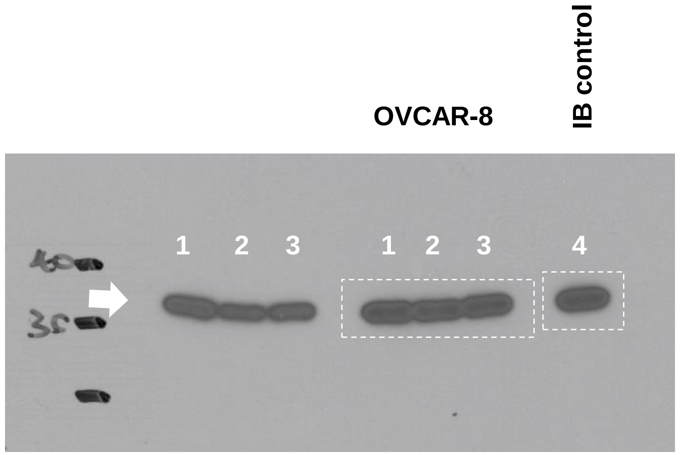

GAPDH band at ~37 kDa

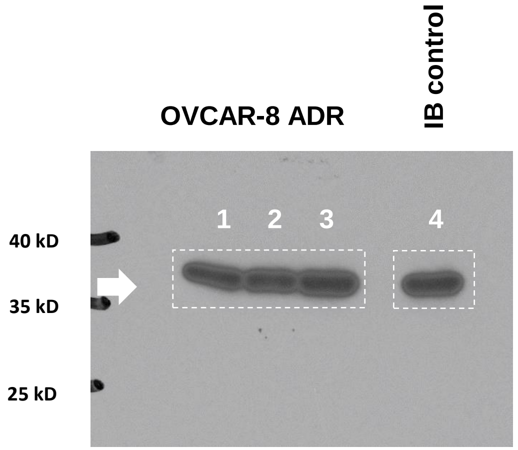

GAPDH band at ~37 kDa

- Dashed rectangle box = cropped region
- Adjusted brightness equally (+20)

Extended Data Figure 6e

BRCA1 Expression

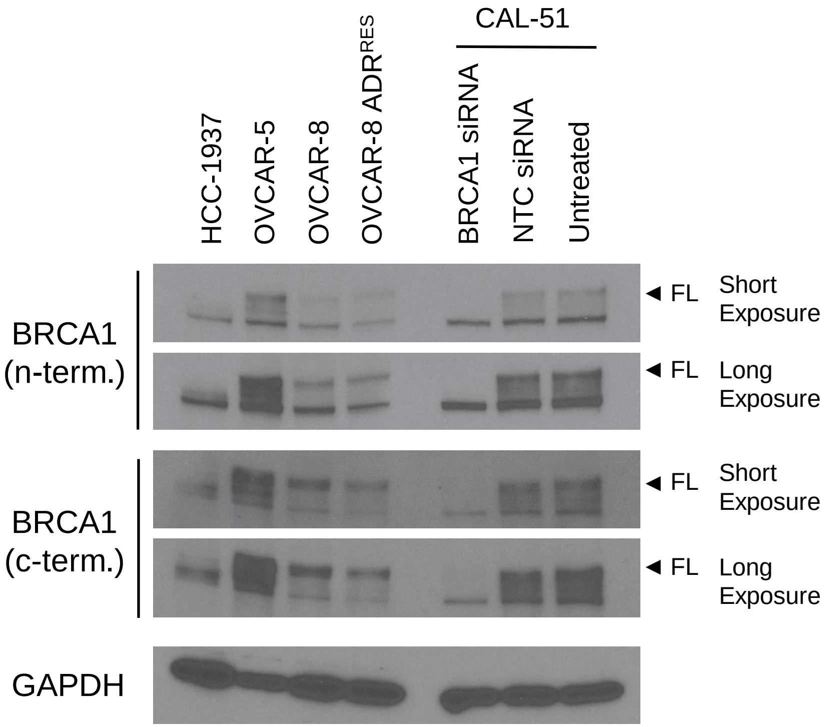

OVCAR-5 and CAL-51 (*BRCA1* wild-type)
HCC-1937 (*BRCA1* mutant)
OVCAR-8 (*BRCA1* promoter methylated)

Adjusted brightness equally (+20)

Immunoblots, uncropped scans of film (BRCA1 expression)

Extended Data Figure 6e

Anti-BRCA1 (n-term)

Rabbit anti-BRCA1 antibody
(1:1000; Cell Signaling, 9010S)

Short exposure

Lane 1 2 3 4 5 6 7

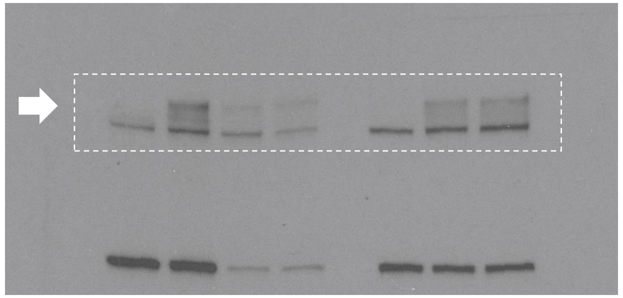

Long exposure

Lane 1 2 3 4 5 6 7

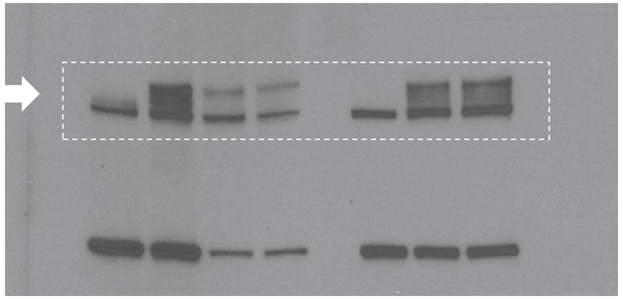

Anti-BRCA1 (c-term)

Rabbit anti-BRCA1 antibody
(1:2000; Millipore, 07-434)

Short exposure

Lane 1 2 3 4 5 6 7

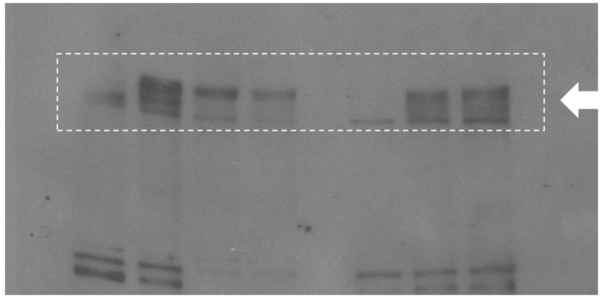

Long exposure

Lane 1 2 3 4 5 6 7

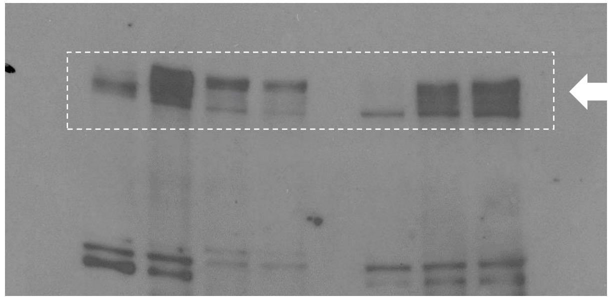

Full length BRCA1 band at ~ 220 kDa
(lower band is nonspecific (e.g. protein band is not depleted with BRCA1 siRNA in lane 5)

Anti-GAPDH

Rabbit anti-GAPDH antibody
(1:10,000; Cell Signaling, 2118S)

Lane 1 2 3 4 5 6 7

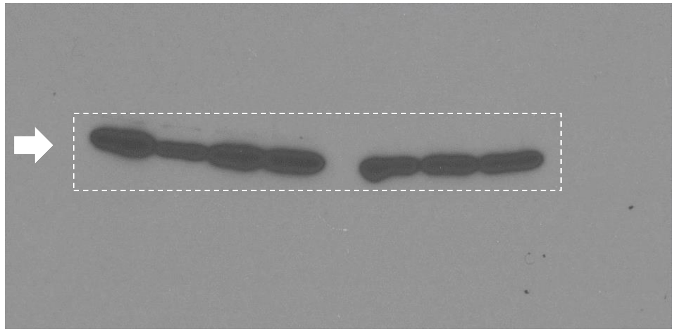

GAPDH band at ~37 kDa

Lane ID	Cell line Information
1	HCC-1937
2	OVCAR-5
3	OVCAR-8
4	OVCAR-8_ADR
	Blank well
5	CAL-51, BRCA1 siRNAs
6	CAL-51, NTC siRNAs
7	CAL-51, Untreated

- Dashed rectangle box = cropped region
- Adjusted brightness equally (+20)

Extended Data Figure 6f

BRCA1 altered HCC-1937 and OVCAR-8 Immunoblots, uncropped scans of film

Apoptosis Analysis

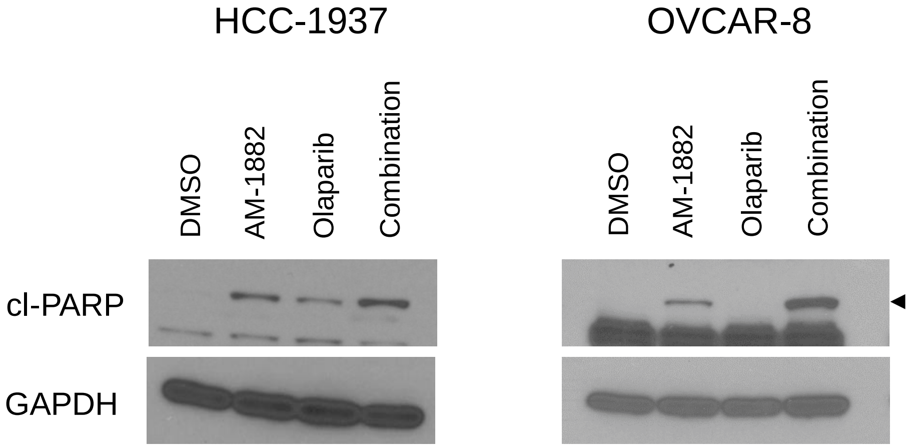

Lane ID	HCC-1937 cells treated for 48 hours
1	DMSO
2	AM-1882 (0.01 µM)
3	Olaparib (20 µM)
4	Combination

Lane ID	OVCAR-8 cells treated for 48 hours
1	DMSO
2	AM-1882 (0.03 µM)
3	Olaparib (5 µM)
4	Combination

Anti-cleaved PARP

Mouse anti-cl-PARP antibody
(1:500; BD Pharmingen, Cat# 552597)

HCC-1937

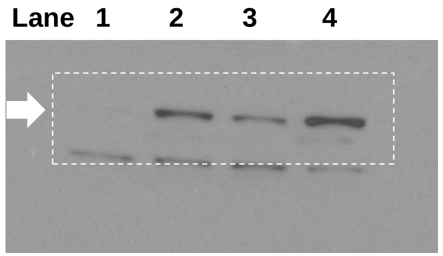

Cl-PARP band at ~89 kDa

OVCAR-8

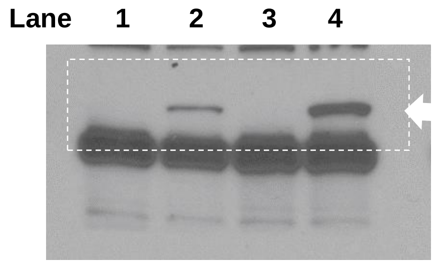

Cl-PARP band at ~89 kDa

Anti-GAPDH

Rabbit anti-GAPDH antibody
(1:10,000; Cell Signaling, 2118S)

HCC-1937

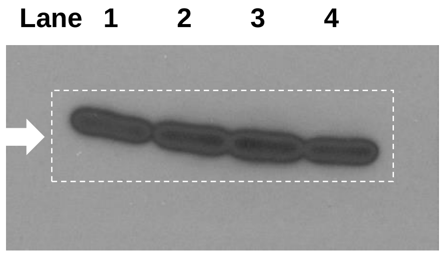

GAPDH band at ~37 kDa

OVCAR-8

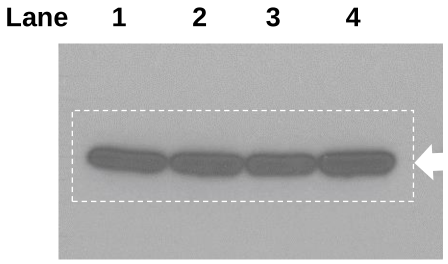

GAPDH band at ~37 kDa

- Dashed rectangle box = cropped region
- Adjusted brightness equally (+20)
